# Parallel and Intertwining Threads of Domestication in Allopolyploid Cotton

**DOI:** 10.1002/advs.202003634

**Published:** 2021-03-15

**Authors:** Daojun Yuan, Corrinne E. Grover, Guanjing Hu, Mengqiao Pan, Emma R. Miller, Justin L. Conover, Spencer P. Hunt, Joshua A. Udall, Jonathan F. Wendel

**Affiliations:** ^1^ Department of Ecology Evolution, and Organismal Biology (EEOB) Bessey Hall Iowa State University Ames IA 50011 USA; ^2^ College of Plant Science and Technology Huazhong Agricultural University Wuhan Hubei 430070 China; ^3^ State Key Laboratory of Crop Genetics and Germplasm Enhancement Cotton Hybrid R & D Engineering Center Nanjing Agricultural University Nanjing 210095 China; ^4^ BioFire Inc. 515 Colorow Dr. Salt Lake City UT 84108 USA; ^5^ Crop Germplasm Research Unit USDA‐ARS College Station TX 77845 USA

**Keywords:** domestication, genome evolution, introgression, selective sweeps, whole genome resequencing

## Abstract

The two cultivated allopolyploid cottons, *Gossypium hirsutum* and *Gossypium barbadense*, represent a remarkable example of parallel independent domestication, both involving dramatic morphological transformations under selection from wild perennial plants to annualized row crops. Deep resequencing of 643 newly sampled accessions spanning the wild‐to‐domesticated continuum of both species, and their allopolyploid relatives, are combined with existing data to resolve species relationships and elucidate multiple aspects of their parallel domestication. It is confirmed that wild *G. hirsutum* and *G. barbadense* were initially domesticated in the Yucatan Peninsula and NW South America, respectively, and subsequently spread under domestication over 4000–8000 years to encompass most of the American tropics. A robust phylogenomic analysis of infraspecific relationships in each species is presented, quantify genetic diversity in both, and describe genetic bottlenecks associated with domestication and subsequent diffusion. As these species became sympatric over the last several millennia, pervasive genome‐wide bidirectional introgression occurred, often with striking asymmetries involving the two co‐resident genomes of these allopolyploids. Diversity scans revealed genomic regions and genes unknowingly targeted during domestication and additional subgenomic asymmetries. These analyses provide a comprehensive depiction of the origin, divergence, and adaptation of cotton, and serve as a rich resource for cotton improvement.

## Graphical abstract

To elucidate the history of parallel domestication in *Gossypium hirsutum* and *Gossypium barbadense*, a diverse assemblage of plants spanning the wild‐to‐domesticated continuum is resequenced. A robust phylogenomic analysis of infraspecific relationships is presented, quantify patterns of genetic diversity in different gene pools, describe genetic bottlenecks associated with domestication and diffusion, detect pervasive genome‐wide bidirectional introgression and signatures of domestication.

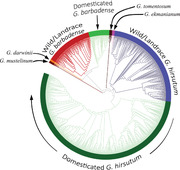

## 1 Introduction

Cotton (*Gossypium* spp.) is one of the most important cash crops in the world, providing the largest source of natural and renewable fiber, as well as edible oil and protein.^[^ ^1^ ^]^ Four cultivated cotton species were domesticated independently by four geographically different civilizations, in each case extending back several thousand years. Two of the domesticated species are A‐genome diploids native to the African and the Arabian Peninsulas (*G*. *herbaceum* L. and *G*. *arboreum* L.). The other two domesticated species are allotetraploids (*Gossypium hirsutum* L. and *Gossypium barbadense* L.) in the Western Hemisphere, each of which contains an A‐genome and a D‐genome from a polyploidization event 1–2 million years ago (MYA).^[^ ^2^ ^]^ Today, more than 97% of the annual fiber production worldwide comes from allotetraploid cottons (*G*. *hirsutum* and *G*. *barbadense*), which were independently domesticated at least 4000 years ago in the Yucatan Peninsula^[^ ^3^, ^4^ ^]^ and northwest South America,^[^ ^5^, ^6^ ^]^ respectively.

Previous surveys of allozyme^[^ ^3^ ^]^ and RFLP^[^ ^4^ ^]^ diversity indicate that there are two genetic diversity centers of *G*. *hirsutum*. One is in southern Mexico‐Guatemala, the primary center of diversity, while the secondary center of diversity that developed as primitive *G*. *hirsutum* cultivars spread throughout the Caribbean and hybridized with *G*. *barbadense*.^[^ ^7^ ^]^ Modern elite *G*. *hirsutum* cultivars derive from the various indigenous Caribbean and Mesoamerican landraces, which were further improved in the southern United States and subsequently dispersed worldwide.

The second domesticated polyploid cotton, *G*. *barbadense*, is indigenous to the coastal areas of Peru.^[^ ^5^, ^6^ ^]^ Following initial domestication west of the Andes, the primary dispersal appears to have been a trans‐Andean expansion into northern South America, which subsequently expanded into Central America, the Caribbean, and the Pacific. Modern elite *G*. *barbadense* cultivars trace their origin to the Sea Island cotton accessions developed on the coastal islands of Georgia and South Carolina USA; these were later improved as Egyptian cotton and Pima cotton.^[^ ^7^ ^]^

Phenotypic variation has been manipulated by humans during crop domestication to make cotton more useful and better adapted to human intervention.^[^ ^8^ ^]^ Cultivated forms of *G*. *hirsutum* and *G*. *barbadense* differ from their wild counterparts in numerous traits, including reduced seed dormancy, increased yield, changes in plant architecture associated with row‐cropping, loss of photoperiod sensitivity, and improved fiber quality.^[^ ^8^, ^9^, ^10^ ^]^ The genetic architecture and developmental mechanisms that underlie this syndrome can be elucidated by comparative genomic analyses between cultivated forms and their wild progenitors.^[^ ^11^, ^12^, ^13^, ^14^ ^]^ Whole‐genome resequencing has been used to decipher the underpinnings of the domestication syndrome in many crops, including rice,^[^ ^15^, ^16^, ^17^ ^]^ maize,^[^ ^18^ ^]^ soybean,^[^ ^19^ ^]^ and others. Recent advances in cotton genome sequencing^[^ ^20^, ^21^, ^22^, ^23^, ^24^, ^25^, ^26^, ^27^, ^28^, ^29^, ^30^, ^31^, ^32^, ^33^ ^]^ have been used to assess the domestication history of *G*. *hirsutum* and *G*. *barbadense*; however, these studies included limited representation of the wild/landrace gene pool from the ancestral geographic centers of origin and/or low coverage sequencing. Accordingly, the present study was designed expressly to assess the effects of domestication and improvement of *G*. *hirsutum* and *G*. *barbadense* on the origin and composition of their modern gene pools.

Here, we employed high‐coverage whole‐genome resequencing for 643 accessions of polyploid cotton, and analyzed a total of 1024 accessions including 795 accessions of *G. hirsutum*, 201 accessions of *G. barbadense*, and 28 solely wild tetraploid species (i.e., *Gossypium mustelinum*, *Gossypium darwinii*, *Gossypium tomentosum*, *Gossypium ekmanianum*, and *Gossypium stephensii*) for the present study (others from).^[^ ^24^, ^25^, ^26^, ^34^ ^]^ Our sampling is distinguished from previous studies by including representatives of all seven^[^ ^2^, ^35^, ^36^ ^]^ tetraploid species and by extensive sampling of *G. barbadense* and *G. hirsutum* populations spanning the wild‐to‐domesticated continuum. We used these data to address the following questions: 1) How much diversity exists in the wild, landrace, and modern cultivated gene pools of the two most agronomically important cotton species? 2) What inferences can be made about the geographic origin of domestication, and how much of the wild diversity has been captured following the multiple genetic bottlenecks accompanying domestication and improvement? 3) How much of the winnowing of variation has been counteracted by historical, human‐mediated interspecific gene flow between the two species, which became sympatric in parts of their indigenous ranges following dispersal from their ancestral homes? In addition, we used these data to detect historically important genomic regions of domestication and of historical interspecific introgression.

## 2 Results

### 2.1 Genomic Variation among Species and Accessions

#### 2.1.1 Sampling

We resequenced 643 accessions selected from the U.S. Cotton Germplasm Collection and our own collections, based on the genetic information of earlier studies,^[^ ^3^, ^4^, ^5^, ^35^, ^36^, ^37^, ^38^, ^39^ ^]^ with a focus on sampling the most diverse and broadly representative collection possible for exploring the specific questions about phylogenetic relationships and apportionment of diversity in wild and domesticated gene pools. Sampling included 442 accessions of *G. hirsutum* (AD_1_; 210 domesticated, 232 accessions spanning the wild‐to‐landrace continuum), 182 accessions of *G. barbadense* (AD_2_; 81 domesticated, 101 accessions spanning the wild‐to‐landrace continuum), and 19 other tetraploid samples (including accessions of *G. tomentosum* (AD_3_)*, G. mustelinum* (AD_4_), *G. darwinii* (AD_5_), *G. ekmanianum* (AD_6_), and *G. stephensii* (AD_7_); Note S1 and Table S1, Supporting Information). Critically, for *G. hirsutum* and *G. barbadense*, accessions were selected to span the range of domestication from fully wild forms to modern elite cultivars, with over half (352 of 641) being wild or landrace, noting that the broad operational category of landrace may include multiple accessions representing feral derivatives that became established following escape from cultivation.^[^ ^3^, ^4^, ^40^ ^]^ We generated an average of 23× coverage for each accession (approximately 42 Terabases for the 2.4 Gigabase tetraploid genome; Note S1 and Table S1, Supporting Information). This high‐quality sequence depth was at least fourfold higher than previous reports,^[^ ^24^, ^25^, ^26^, ^27^, ^32^ ^]^ substantially increasing our ability to quantify and analyze genetic variation (Note S2, Supporting Information). Additional sequencing data from a further 789 tetraploid accessions were compiled from collaborators and previous publications for a total of 1432.^[^ ^24^, ^25^, ^26^, ^34^ ^]^

Accessions with more than 25% missing sites were discarded, resulting in the removal of 408 samples (Note S2, Supporting Information). The remaining 1024 samples were composed of 795 *G. hirsutum*, 201 *G. barbadense*, and 28 samples from the remaining wild tetraploid species (i.e., 7 *G. tomentosum*, 6 *G. mustelinum*, 5 *G. darwinii*, 6 *G. ekmanianum*, 2 *G. stephensii*, and 2 synthetic allotetraploid accessions; **Table** **1**). Approximately 40% of all sequenced accessions were either wild or landrace accessions, significantly increasing the representation of non‐cultivated accessions and providing the necessary foundation for understanding changes in genetic architecture and diversity during cotton domestication.

**Table 1:** Number and distribution of polyploid cotton accessions sequenced and/or analyzed. Numbers in parentheses indicate the number of newly sequenced accessions. The full list of accessions can be found in Note S1 and Table S1, Supporting Information

Species	Newly sequenced^a)^	Previous^b)^	Total	Wild or Landrace^c)^	Domesticated^c)^
*G. hirsutum*	441	354	795	247 (232)	525 (189)
*G. barbadense*	182	19	201	114 (101)	72 (66)
*G. tomentosum*	5	2	7	7(6)	0
*G. mustelinum*	4	2	6	6(4)	0
*G. darwinii*	4	2	6	6(4)	0
Other tetraploids	5	4	9	9(5)	0
Total	641	383	1024	380	597

^a)^ One accession from each *G. tomentosum a*nd *G. darwinii* were removed due to low mapping rates, bringing the number of newly sequenced accessions used in this study down to 641 from 643;

^b)^ The number of previously resequenced accessions passing quality filters;

^c)^ The number in brackets indicates the number of accessions newly sequenced in this project.

#### 2.1.2 SNP Diversity

Read depth for each accession that passed filtering was relatively high, ranging from 13.4 to 45.9‐fold coverage (mean = 22.8; median = 22.6), from which we identified 53.7 million (M) SNPs within and among the seven tetraploid species. When partitioned by the two co‐resident subgenomes of allopolyploid cotton, the A subgenomes (A_T_) contain over 1.7× SNPs relative to the D subgenomes (D_T_), that is, 34.1M vs 19.6M SNPs, respectively (Note S3, Table S1, and Figure S1, Supporting Information); this difference is congruent with the nearly twofold difference in genome size between the allopolyploid A_T_ and D_T_ genomes^[^ ^41^ ^]^ and echoes earlier results.^[^ ^27^ ^]^ Notably, the number of SNPs identified here is three‐to‐15‐fold greater than found in previous studies,^[^ ^24^, ^25^, ^26^, ^27^, ^32^, ^34^ ^]^ due to the greater sequencing depth and breadth of sampling. Despite the fourfold higher representation of *G. hirsutum* versus *G. barbadense* accessions, a similar number of SNPs were found, that is, 23.0M and 26.6M, respectively (Note S3, Table S1, and Figure S1, Supporting Information). The number of SNPs detected between *G. hirsutum* and *G. barbadense* (33.8M) was approximately 1.5× greater than within species, ≈6% (2.1M) of which were found within or adjacent to gene regions (Note S3 and Figure S2, Supporting Information). Although the A_T_‐genomes had nearly twice the number of SNPs (relative to D_T_), the proportion of gene‐associated SNPs detected in the D_T_‐genome was nearly twofold higher, because the gene space occupies a higher fraction of the genome (Note S3 and Figure S1, Supporting Information). When comparing SNPs in gene regions within *G. hirsutum* to the number within *G. barbadense*, a slightly greater proportion of SNPs were detected in the gene regions of *G. hirsutum* (4.29%; 0.99M out of 22.99M SNPs) than in *G. barbadense* (3.65%; 0.97M out of 26.64M SNPs; Note S3, Table S1, and Figure S2, Supporting Information).

#### 2.1.3 Small Indel Diversity

Diversity in small indels (<10 bp; relative to the *G. hirsutum* reference) also contributes variation to the genomes of polyploid cotton, ranging from 0.96–4.59 million indels per species (Note S3 and Table S2, Supporting Information), some of which were shared among accessions and/or lineages. Of the 5.9M indels identified, most (89%; 5.3M indels) were 1–3 bp in length (Note S3 and Table S3, Supporting Information). While over half were located in the A_T_‐genome, this is largely due to the excess of intergenic indels in the twofold larger A_T_‐genome (Note S3 and Table S4, Supporting Information). For indels located within or adjacent to genes, the A_T_‐genome had slightly fewer indels than the D_T_‐genome (Note S3 and Table S4, Supporting Information). Fewer than 30% of all gene‐associated indels were located within the gene region (including UTRs) in either subgenome, most of which were in introns. Interestingly, of the 46 347 indels located in exons, approximately 70% were not in multiples of three, indicating missense potential; this pattern was similar for both subgenomes. More indels (4.6M) were detected in *G. hirsutum* and *G. barbadense* (3.9M) than in the less intensively sampled species, with the fewest in the island endemics *G. stephensii* (0.1M) and *G. tomentosum* (0.5M). In general, indel placement in the genome was consistent among species (Note S3 and Table S2, Supporting Information), with ≈77% of indels (per species) found in intergenic regions, 10% upstream of genes, 6% downstream, and 8% within genes (most in introns).

### 2.2 Population Structure and Diversity within and Between Cotton Species

**Figure 1:**
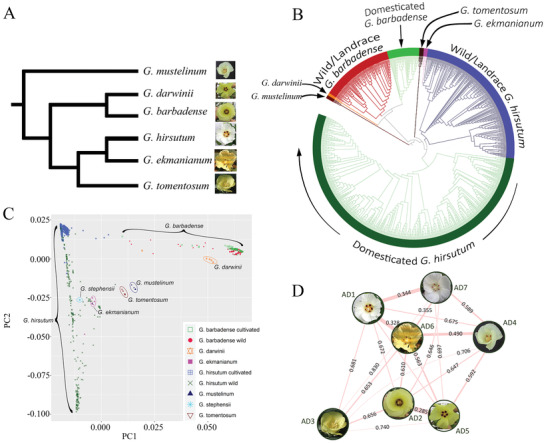
A) Expected species relationships based on previous research.^[^ ^30^, ^36^, ^43^ ^]^ B) Phylogenetic relationships among resequenced accessions (*G. stephensii* not shown). C) PCA of SNP diversity within and among species. As expected *G. ekmanianum* and *G. stephensii* are close to but not included within wild *G. hirsutum*.^[^ ^35^, ^36^, ^43^ ^]^ Individual species are circumscribed/bracketed; however, small numbers of disjunct accessions are found for *G. ekmaninum* (2), *G. mustelinum* (1), *G. darwinii* (1), *G. barbadense* (1), and *G. hirsutum* (2; see Note S3 and Figure S3, Supporting Information). D) Divergence among the 7 allopolyploid cotton species. Weighted *F*_st_ is depicted as lines among species; species are abbreviated as follows: AD_1_ (*G. hirsutum*), AD_2_ (*G. barbadense*), AD_3_ (*G. tomentosum*), AD_4_ (*G. mustelinum*), AD_5_ (*G. darwinii*), AD_6_ (*G. ekmanianum*), and AD_7_ (*G. stephensii*). The width of the lines scale with 1‐*F*_st_.

Maximum‐likelihood (ML) analysis of fourfold degenerate sites was used to evaluate phylogenetic relationships within and between the two domesticated allotetraploid cotton species (**Figure** **1**A,B). Based on previous studies that justify the choice of the allopolyploid species *G. mustelinum* as an outgroup^[^ ^35^, ^36^, ^42^, ^43^ ^]^ to the other allopolyploids, phylogenetic analyses led to the recovery of two main clades: 1) a clade that includes *G. barbadense* and *G. darwinii*, and 2) a second clade that includes *G. hirsutum*, *G. tomentosum*, *G. ekmanianum*, and *G. stephensii*. Both of these results are consistent with previously reported phylogenetic data,^[^ ^30^, ^35^, ^36^, ^43^ ^]^ documenting the early division of *Gossypium* allopolyploids into two groups, the “barbadense” and “hirsutum” groups, following their joint divergence from the lineage that gave rise to modern *G. mustelinum*. These same relationships are generally reflected in the principal component analysis using all of the SNP data (PCA, Figure 1C; PCA1 and PCA2 explain 77.2 and 14.2% of the variance, respectively). The two recently recognized allotetraploid species, that is, *G. ekmanianum* and *G. stephensii*, are closely associated with *G. hirsutum* germplasm, with which they are often confused.^[^ ^35^, ^36^ ^]^

Genetic diversity among accessions of each allotetraploid species generally is low and is evenly distributed between the A_T_ and D_T_‐genomes. The Brazilian *G. mustelinum* exhibited the highest genetic diversity (*π* = 2.91 × 10^–3^), whereas the island endemic *G. stephensii* exhibited the lowest (*π* = 0.51 × 10^–3^). For the two domesticated species (**Table** **2**), overall nucleotide diversity was higher among *G*. *barbadense* accessions (2.05 × 10^–3^) than among *G. hirsutum* accessions (1.65×10^–3^), despite the more abundant and broader sampling of *G. hirsutum* accessions, but also reflecting the higher number of highly similar cultivated samples in the latter species. In general, nucleotide diversity was higher than previously reported,^[^ ^24^, ^25^, ^26^, ^27^, ^34^ ^]^ due to the increased representation of wild accessions and sequencing depth (Note S2, Supporting Information). As expected, most of the diversity was in intergenic regions, with *G. barbadense* exhibiting more diversity (2.4 × 10^–3^) than *G. hirsutum* (2.2 × 10^–3^); this difference, in fact, accounts for the overall greater diversity in *G. barbadense* than *G. hirsutum* (Table 2). Intergenic diversity in *G. barbadense* is similar among landraces and among cultivated accessions, whereas in *G. hirsutum* cultigens exhibited a two‐to‐threefold reduction in diversity compared to landrace or wild accessions (Table 2; Note S3 and Figure S4, Supporting Information). Genic diversity in the two cultivated species was similar (Table 2). Using comparative diversity as a metric for the bottleneck experienced during domestication, diversity was marginally decreased in *G. barbadense* (from 0.0021 to 0.0018) but more than halved in *G. hirsutum* (from 0.0017 to 0.0008; Note S3 and Figure S5, Supporting Information).

**Table 2:** Diversity (*π*) for *G. hirsutum* (Gh) and *G. barbadense* (Gb), partitioned among genomic regions

	*Gossypium hirsutum*						*Gossypium barbadense*		
	All	Wild	Landrace1	Landrace2	Landrace^a)^	Cultivar	All	Landrace^a)^	Cultivar
Intergenic	0.0022	0.0028	0.0024	0.0026	0.0030	0.0011	0.0024	0.0019	0.0019
Upstream	0.0019	0.0022	0.0019	0.0021	0.0024	0.0009	0.0017	0.0014	0.0014
Gene	0.0009	0.0013	0.0011	0.0011	0.0013	0.0005	0.0010	0.0010	0.0009
5′UTR	0.0017	0.0034	0.0025	0.0026	0.0027	0.0015	0.0016	0.0012	0.0012
Exon	0.0009	0.0013	0.0011	0.0012	0.0013	0.0005	0.0010	0.0009	0.0009
Intron	0.0010	0.0014	0.0012	0.0013	0.0014	0.0006	0.0011	0.0011	0.0010
3′UTR	0.0013	0.0023	0.0018	0.0018	0.0020	0.0010	0.0012	0.0009	0.0009
Downstream	0.0015	0.0019	0.0016	0.0017	0.0019	0.0008	0.0015	0.0013	0.0013
Overall	0.0017	0.0025	0.0020	0.0023	0.0052	0.0008	0.0021	0.0025	0.0018
Overall (At)	0.0017	0.0024	0.0020	0.0023	0.0052	0.0008	0.0021	0.0025	0.0018
Overall (Dt)	0.0016	0.0025	0.0022	0.0022	0.0051	0.0008	0.0020	0.0025	0.0018

^a)^ all landrace accessions, from both subpopulations.

Using the same SNP set, we measured relatedness among cotton species for the whole genome (Figure 1C,D), as well as separately for each subgenome using weighted *F*_st_ (Note S3 and Figure S6, Supporting Information). The largest differentiation was found between the island endemic *G. tomentosum* (Hawaiian Islands) and other wild endemics, namely *G. darwinii* (Galapagos Islands), *G. stephensii* (Line Islands), and the outgroup *G. mustelinum* (NE Brazil). Conversely, differentiation between *G. barbadense*‐*G. darwinii* and among *G. ekmanianum*‐*G. hirsutum*‐*G. stephensii* were both remarkably low, as expected from previous analyses.^[^ ^30^, ^36^, ^43^ ^]^ Weighted *F*_st_ between *G. hirsutum* and *G. barbadense* (0.67; Note S3 and Figure S6, Supporting Information) was comparable to that previously reported (0.63–0.65).^[^ ^25^ ^]^

### 2.3 *G. hirsutum* Population Structure, Genetic Diversity, and Domestication

**Figure 2:**
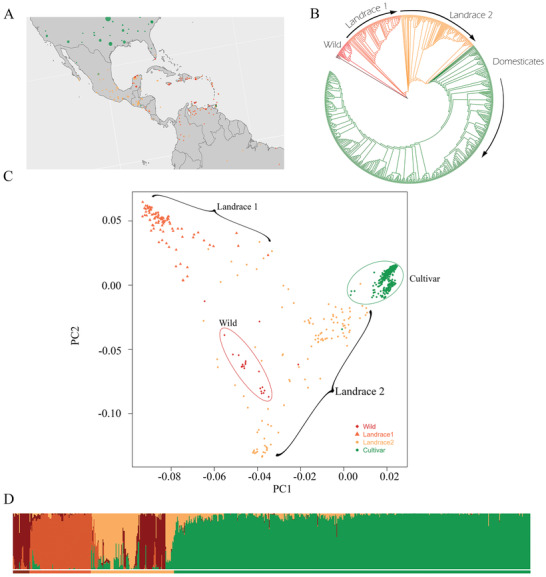
A) The distribution map for *G. hirsutum* accessions, both wild/landrace (red) and modern domesticated (green). High‐resolution maps and location data are available at https://github.com/Wendellab/BYUReseq. B) Phylogenetic relationships among accessions of *G*. *hirsutum* using *G. mustelinum* as an outgroup (in brown). Phylogeny with labeled samples is available in Note S3, Figure S7, Supporting Information, and https://github.com/Wendellab/BYUReseq. C) PCA of SNP diversity among wild (red), landrace 1 (orange), landrace 2 (yellow), and domesticated (green) accessions (PC1 and PC2 account for 64.3 and 20.4% of the variance, respectively). Ovals are drawn to include most representatives of each group. PCA with labeled samples is available in Note S3, Figure S8, Supporting Information, and https://github.com/Wendellab/BYUReseq. D) STRUCTURE‐based population identifications, specifying four populations: wild (red), landrace 1 (orange), landrace 2 (yellow), and domesticated (green). Colored horizontal bars below the STRUCTURE plot indicate inferred population identity.

Four distinct groups were identified from the 795 *G. hirsutum* accessions evaluated (**Figure** **2**A), designated here as 1) Wild, 2) Landrace 1, 3) Landrace 2, and 4) Cultivars. While these partitions were primarily diagnosed via phylogenetic analysis (Figure 2B), these divisions were also supported by PCA and STRUCTURE (Figure 2C,D). Wild *G. hirsutum* (N = 54) was primarily geolocated on the Yucatan peninsula, somewhat between the growing regions of the two landrace groups (Figure 2B). Landrace 1 (N = 85) primarily bordered the Caribbean Sea (e.g., Brazil, Venezuela, Colombia, Haiti, Puerto Rico, Dominican Republic, Jamaica, and other island nations of the Caribbean archipelago; Figure 2A). Landrace 2 (N = 108) was generally composed of accessions originating from Central America (e.g., Mexico, Guatemala, Belize, and Honduras). Phylogenetic analysis supports the hypothesis, advanced more than 25 years ago based on allozyme^[^ ^3^ ^]^ and RFLP^[^ ^4^ ^]^ data, that modern cotton cultivars were derived from a gene pool from the geographic region occupied by the latter accessions (i.e., Landrace 2; Figure 2B). As expected, cultivated accessions cluster tightly in the PCA, reflecting their narrow genetic diversity (Figure 2C). Landraces are clearly distinct from the wild accessions and encompass much greater diversity than the cultivars, as reflected in their spatial breadth in the PCA.

Overall (whole genome) nucleotide diversity (*π*) among *G. hirsutum* cultivars (*π* = 0.75 × 10^–3^) was about one third that found in the other three groups (*π* = 2.47 × 10^–3^, 2.03 × 10^–3^, and 2.26 × 10^–3^, for wild, Landrace 1, and Landrace 2, respectively; Table 2), lending a quantitative perspective to the severity of the bottleneck experienced during modern crop improvement and cultivar development. In contrast, wild accessions contain only slightly more diversity than that found in the two landrace groups, possibly reflecting less intense selection and other population‐level phenomena (such as gene flow) during the long slow process of initial domestication and cotton improvement.^[^ ^44^ ^]^ Except for Landrace 2, nucleotide diversity (*π*) was slightly higher among D_T_‐genomes than A_T_‐genomes for each group (Table 2); however, when all accessions were combined, the overall genetic diversity among D_T_‐genomes was slightly lower than among A_T_. Diversity among gene regions followed the same general pattern for all groups whereby diversity within the gene body was lower than in intergenic or UTR regions (Table 2; Note S3 and Figure S5, Supporting Information). As expected, diversity within intergenic regions was consistently more than twofold greater than in genic regions (“gene”, Table 2). Interestingly, diversity within the 5’‐UTR regions of genes was not only higher than the other transcript regions (per group), but it was also frequently higher than diversity in the upstream/downstream and/or intergenic regions, which are subjected to the less selective constraint (Table 2; Note S3 and Figure S5, Supporting Information). When all accessions were considered together, however, this excess diversity in the 5’‐UTR relative to the upstream and intergenic regions largely disappeared. Interestingly, within coding regions, diversity within fourfold degenerate (4D) sites (except for wild accessions of *G. hirsutum*) was higher than for synonymous and nonsynonymous sites (Note S3 and Figure S4, Supporting Information), and similar diversity between the latter, suggesting that the transitions to landrace and domesticated forms may have included both changes in amino acids and codon usage preferences.

### 2.4 *G. barbadense* Genetic Diversity, Population Structure, and Domestication

**Figure 3:**
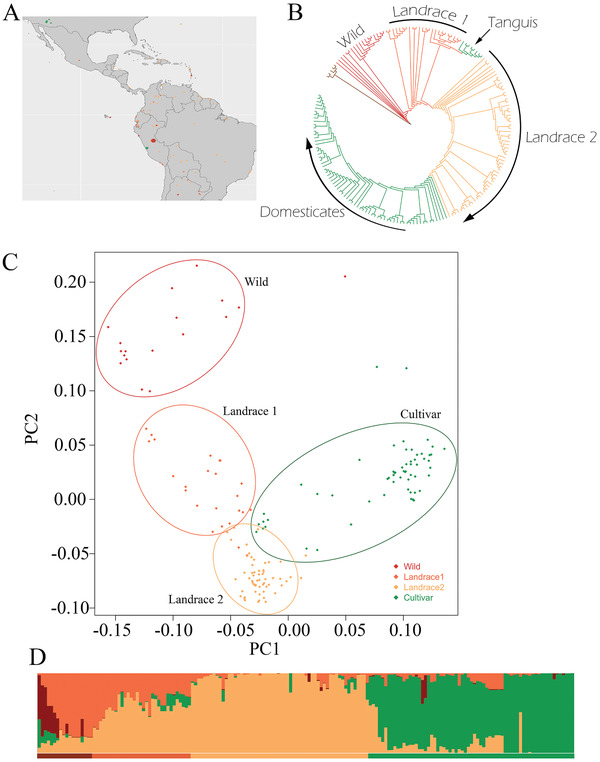
A) Distribution of putatively wild or landrace (red) and cultivated (green) accessions of *G. barbadense*. High‐resolution maps and location data are available at https://github.com/Wendellab/BYUReseq. B) Phylogenetic relationships among *G. barbadense* accessions, using *G. mustelinum* (brown) as an outgroup. Wild accessions are in red, landraces in orange, and domesticated in green. Tanguis cottons are noted in green in landrace 1. Tree with labeled samples is available in Note S3, Figure S9, Supporting Information, and https://github.com/Wendellab/BYUReseq. C) PCA of SNP diversity among wild, landrace, and cultivated accessions of *G. barbadense* (PC1 and PC2 account for 38.9 and 33.2% of the variance, respectively). Ovals are drawn to include most representatives of each group. PCA with labeled samples is available in Note S3, Figure S10, Supporting Information, and https://github.com/Wendellab/BYUReseq. D) STRUCTURE‐based population identification, specifying four populations: wild (red), landrace 1 (dark orange), landrace 2 (light orange), and cultivar (green). Colored horizontal bars below the STRUCTURE plot indicate inferred population identity.

Phylogenetic analysis of 201 *G. barbadense* accessions (**Figure** **3**A) similarly revealed four distinct groups, namely Cultivars, Landrace 1 plus Tanguis, Landrace 2, and Wild; Figure 3B), which was also supported by PCA and STRUCTURE (Figure 3B,C). Wild *G. barbadense* grows mainly in the intermontane regions of the NW Andes, where it was originally domesticated. The two landraces are primarily divided by the Andean mountain range. Landrace 1 plus Tanguis was generally composed of accessions west of the Andes (Peru and Ecuador) and Landrace 2 was generally composed of South American and/or Caribbean accessions originating east of the Andes. Notably, the Peruvian Tanguis cotton accessions appear as part of Landrace 1, despite their status as currently cultivated cotton.^[^ ^45^, ^46^ ^]^ The remaining *G. barbadense* cultivars represent the majority of *G. barbadense* grown in cultivation, and these also cluster into subgroups (i.e., Sea Island, Egyptian, and Pima) based on their history of domestication.

The genetic diversity in *G. barbadense* is low (2.00 × 10^–3^, 1.68 × 10^–3^, 1.53 × 10^–3^, and 1.76 × 10^–3^ for wild, Landrace 1, Landrace 2, and cultivar groups, respectively; Table 2); however, unlike *G. hirsutum*, a dramatic reduction in genetic diversity associated with selection under domestication is not observed in the cultivars (Table 2). While this may be due to the underrepresentation of diversity in the wild and/or landrace accessions sequenced here, it also speaks to the complex and obscure origins of modern cultivated *G. barbadense*, which includes multiple distinct lineages and a known history of intentional introgression^[^ ^8^, ^47^ ^]^ (Figure 3D).

In *G. barbadense*, overall genetic diversity among D_T_‐genomes is slightly less than among A_T_‐genomes; however, this slight bias in genetic diversity between subgenomes was also reflected within groups, in contrast to *G. hirsutum* (Table 2). At the gene level, the patterns of diversity largely mirrored those seen in *G. hirsutum* (Note S3, Table 1, and Figures S5, S6, Supporting Information), although the remarkable diversity seen in 5’‐UTR in *G. hirsutum* was not present among *G. barbadense* accessions (Note S3, Table 1, and Figure S7, Supporting Information). As with *G. hirsutum*, diversity in 4D sites was generally higher than for other synonymous sites, as observed in *G. hirsutum* (Note S3 and Figure S4, Supporting Information).

### 2.5 Signatures of Selection in *G. hirsutum* and *G. barbadense*

**Figure 4:**
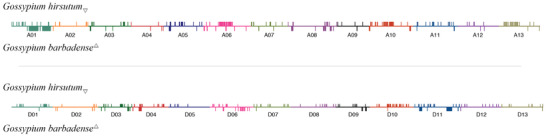
Putative regions of selection on the A_T_ chromosomes (top) and D_T_ chromosomes (bottom). For each set of chromosomes, *G. hirsutum* regions are depicted above each line, whereas *G. barbadense* regions are placed below each line. Although pictured here on identical scales, the A_T_ chromosomes are about twice the length of D_T_ chromosomes.

Because the modern gene pools of cultivated *G. hirsutum* and *G. barbadense* were derived from their respective landrace antecedents, we searched for signatures of selection in the modern cultivars of both species using sliding windows (100‐kilobase (kb) windows, sliding in 20 kb steps) to identify regions of reduced diversity and/or increased differentiation between the modern cultivars and the Landrace 2 of each species (from which they derive). The top 5% of regions with the greatest reduction in overall diversity (Δ*π*) and the top 5% *F*_st_ value provided evidence in support of selection during domestication (Note S4, Table S1, Supporting Information, and Figure 1), although demographic factors during the many millennia of cotton domestication and diffusion might also generate similar patterns of diversity reduction. These metrics identified 438 regions (in 1442 sliding windows) with evidence of reduced genetic diversity (**Figure** **4**) in *G. hirsutum*, representing approximately 3% (65 MB) of the genome. Although only the A_T_ parent possesses spinnable fiber, these regions of selection were nearly evenly distributed between both subgenomes of *G. hirsutum*, both in number (247 A_T_ vs 191 D_T_) and total length (35 MB A_T_ vs 30 MB D_T_) of the putatively selected region. Although both the number and total length of regions in A_T_ was greater, there were approximately one third fewer genes contained within the A_T_ regions (795 in A_T_ and 1226 in D_T_). When compared to a recent QTL analysis evaluating the transition from wild to domesticated *G. hirsutum*,^[^ ^48^ ^]^ nearly two‐thirds of the selective sweep regions were contained within a QTL (65%; Note S4 and Table S1, Supporting Information), including 155 regions containing fiber QTL (551 genes). The overlap with QTL from recent^[^ ^49^ ^]^ and metaQTL^[^ ^50^ ^]^ analyses was also detected, including 6 QTL associated with fiber improvement in the former study (Note S4 and Table S1, Supporting Information). Expression levels for the 2021 genes found within the fiber‐associated putative selective sweep regions suggested that 17.9% (i.e., 362 genes) are preferentially expressed in fibers (Note S4, Table 2, and Figure S2, Supporting Information), and 157 genes (7.8% of total putative selective genes) appear to have expression levels changed by domestication (Note S4, Table S3, and Figure S3, Supporting Information).

Analysis of signatures of selection in *G. barbadense* led to the detection of fewer regions of putative selection (261 vs 438 regions in *G. hirsutum*), although a similar amount of the genome was affected (≈3%; 70 MB vs 65 MB in *G. hirsutum*; Figure 4; Note S4 and Table S4, Supporting Information). In contrast to *G. hirsutum*, the regions of selection detected in the A_T_‐genome are both twice in number and total length relative to D_T_ (168 regions comprising 48.34 MB in A_T_ vs 93 regions comprising 21.54 MB in D_T_; Note S4 and Table S5, Supporting Information). Slightly more genes of *G. barbadense* (2347) are in the putatively selected regions than in *G. hirsutum* (2021). Unlike *G. hirsutum*, the A_T_ genes (1317) are more numerous than D_T_ genes (1010) in selected regions. Overlap of putatively selected regions between the two species is relatively small (22 regions; Figure 4) and includes only 148 genes (6–7% from either species; Note S4 and Table S6, Supporting Information); however, although the length of overlap between species (3.1 Mb) is greater than expected (in 1000 permutations). Expression of genes within all putatively selected regions in *G. barbadense* reveals 287 genes (12.3% of total putative selective genes) preferentially expressed in fiber tissue (relative to other tissues, Note S4, Table S7, and Figure S4, Supporting Information), and 208 genes (8.9% of total putative selective genes) show differential expression between wild and domesticated samples (Note S4, Table S8, and Figure S5, Supporting Information). While GO enrichment of genes putatively under selection suggest that different categories of genes have been under selection in *G. hirsutum* versus *G. barbadense* (Note S4 and Figure S6, Supporting Information), we note that both lists include genes implicated in fiber development (Note S4 and Table S6, Supporting Information), including those encoding transcription factors (e.g., MYB, WD40), those involved in hormone signaling (e.g., auxin, gibberellic acid), and biosynthesis of macromolecules (e.g., actin, cellulose synthase).

### 2.6 Introgression between *G. hirsutum* and *G. barbadense*

The global history of cotton is intertwined with the history of industrialization,^[^ ^51^ ^]^ and thus cultivated lines and populations of *G. hirsutum* and *G. barbadense* were often brought into close proximity, raising the prospects for accidental and intentional interspecific introgression. Given this history, we evaluated our dataset for accessions containing reciprocal genetic signatures from wild accessions of the ‘other’ species, since wild accessions should be devoid of human‐mediated interbreeding. Introgression of *G. hirsutum* into *G. barbadense* (and vice versa) was inferred using a subset of wild accessions and methodology similar to^[^ ^34^ ^]^ (see method verification, Note S5, including Note S5, Table S1 and Note S5, Figures S1–S12, Supporting Information). Briefly, we used species‐specific SNPs (generated from multiple wild accessions each) to diagnose genomic regions as introgressed or non‐introgressed and merged adjacent SNPs (<30 kb apart) from the same category (introgressed or not) to characterize putative regions of introgression (see Experimental Section). Recent hybrid accessions, identified both by their intermediate positions on the PCA (Note S2 and Figure S5, Supporting Information) and by having an excess of introgression (see Experimental Section), were excluded.

#### 2.6.1 Introgression from *G. barbadense* into *G. hirsutum*

**Figure 5:**
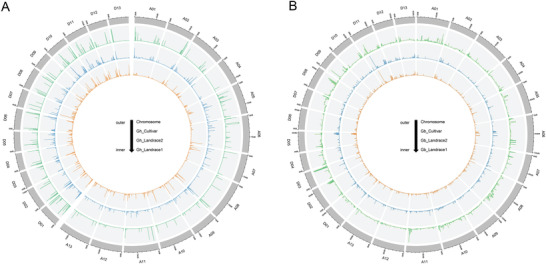
Reciprocal introgression in A) *G. hirsutum* and B) *G. barbadense*. Subpopulations of each species are shown, that is, cultivar (green), landrace 1 (blue), and landrace 2 (orange). The vertical axis of each circle represents the proportion of accessions from each population exhibiting interspecific introgression in *G. hirsutum* (A) and *G. barbadense* (B).

Introgression from *G. barbadense* into *G. hirsutum* was detected in 509 accessions, including 89 from Landrace 1, 111 from Landrace 2, and 309 of the modern domesticated cotton (Note S5 and Table S2, Supporting Information). Evidence of introgression was present on every chromosome in at least one accession of the Landrace 1, Landrace 2, and domesticated populations (**Figure** **5**A), with most accessions exhibiting introgression on over half of the chromosomes (median = 17). The total length of introgressed regions averaged 184 kb per accession (**Table** **3**), including, on average 2.4 introgressed regions per chromosome per accession (Note S5 and Table S2, Supporting Information). Landrace 1 exhibited the most introgression (average 7.5 Mb introgression per accession), 1.7x greater than the average amount of introgression in modern cultivars (4.4 Mb) and over twice the average amount as in Landrace 2 (3.6 Mb). Among accessions, the total length of introgression was relatively low, with accession TX‐2489 (Landrace 1) exhibiting the highest level, that is, 40.4 Mb or 1.7% of the genome (Note S5 and Table S3, Supporting Information). We note, however, that detecting introgression between *G. hirsutum* and *G. barbadense* is naturally limited by the relatively small divergence between the two species; therefore, our estimates should be considered conservative.

**Table 3:** Introgression in *G. hirsutum* and *G. barbadense*, reported as averages across accessions and partitioned by cultivation status. See tables in Note S5, Supporting Information, for more information

		*G. hirsutum*			*G. barbadense*		
		number of accessions	A chromosomes	D chromosomes	number of accessions	A chromosomes	D chromosomes
	all accessions	509	6	9	143	3	5
	cultivar	309	5	8	60	4	5
	LR1	89	8	10	31	4	4
	LR2	111	5	9	52	2	4
		all chromosomes	A chromosomes	D chromosomes	all chromosomes	A chromosomes	D chromosomes
regions^a)^	all accessions	2.4	1.7	3.1	1.5	1.1	1.9
	cultivar	2.2	1.4	2.9	2.6	2.0	3.1
	LR1	3.8	3.3	4.2	0.7	0.7	0.7
	LR2	2.0	1.4	2.6	0.8	0.4	1.3
total length^b)^	all accessions	4.8	2.3	2.5	3.6	1.9	2.1
	cultivar	4.4	2.1	2.3	6.8	3.1	3.7
	LR1	7.5	3.4	4.1	0.9	0.5	0.3
	LR2	3.6	1.8	1.8	1.7	0.5	1.1
number of genes^c)^	all accessions	7.8	5.8	9.8	5.1	3.4	6.8
	cultivar	7.3	5.3	9.2	8.8	6.2	11.4
	LR1	12.0	9.7	14.3	1.8	1.6	2.0
	LR2	5.9	4.1	7.7	2.8	1.2	4.4

^a)^ average number of independent, introgressed regions (<30 kb apart, see Experimental Section) per accession;

^b)^ average length of introgressed segments per accession, in Mb;

^c)^ average number of introgressed genes per accession.

Although the total length of introgression per accession was typically less than 1% of the genome, genic regions were slightly overrepresented, with 6.7% of the gene models in *G. hirsutum* (4421 genes out of 65 636 total gene models) exhibiting evidence of introgression in at least one accession (Note S5 and Table S4, Supporting Information). On average, genes exhibiting introgression were detected in 11 accessions, ranging from introgression in only 1 to a maximum of 213 accessions (out of 509 accessions total), although this distribution was left‐skewed (median = 1 accession per gene). Each accession averaged 7.8 introgressed genes per chromosome (Table 3), with a maximum of 49 genes per chromosome for accession TX‐2415 (Note S5 and Table S4, Supporting Information). Once again, the Caribbean cotton exhibited the most genic introgression (Note S5 and Table S4, Supporting Information), averaging 12 introgressed genes per chromosome per accession, followed by the modern cultivars (7.3). Of the 4421 introgressed genes, 15 also coincided with identified regions under selection (Note S5 and Table S5, Supporting Information), distributed nearly evenly between the A and D subgenomes (7 A_T_ and 8 D_T_).

Strikingly, introgression was asymmetric between the two subgenomes, with more introgression in the D_T_ genome (Table 3). About 70% more genes from the D_T_ genome exhibited introgression in at least one accession than from the A_T_ genome (2788 vs 1633), and more accessions shared the introgressed gene (average 7.7 accessions for D_T_ vs 6.6 for A_T_‐derived genes; Note S5 and Table S5, Supporting Information). On a per accession basis, more genes were introgressed on D_T_ chromosomes (on average) than on A_T_ chromosomes, that is, 9.8 versus 5.8, respectively (Table 3). This general bias is also present for the average number of introgressed regions and an average total length of introgressed segments, that is, an average of 40 regions totaling 2.5 Mb on D chromosomes versus 22 regions totaling 2.3 Mb on A chromosomes (Note S5 and Table S3, Supporting Information). Interestingly, however, while only three chromosomes of the A_T_ genome exhibited a higher than average number of genes introgressed (per chromosome per accession), two of these chromosomes, that is, ChrA01 and ChrA06, have been previously implicated in domestication.^[^ ^30^, ^48^ ^]^

#### 2.6.2 Introgression from *G. hirsutum* into *G. barbadense*

Introgression from *G. hirsutum* was detected in all 143 accessions of *G. barbadense* surveyed, including 60 modern cultivars, 31 accessions of Landrace 1 plus Tanguis, and 52 accessions of Landrace 2 (Note S5 and Table S6, Supporting Information). As with *G. hirsutum*, introgression was detected for every chromosome in at least one accession, but the typical *G. barbadense* accession contained introgressed segments on only a third of its chromosomes (Figure 5B). Whereas the *G. hirsutum* accessions averaged 4.8 Mb of total introgressed sequence per accession, *G. barbadense* averaged 3.6 Mb (Table 3). Cultivar (mutant) M437_9 (Note S5 and Table S6, Supporting Information) exhibits the greatest amount of introgression (2.4 Mb), largely attributable to 21.7 and 20.5 Mb of sequence introgressed on ChrD12 and ChrD04, respectively (Note S5 and Table S7, Supporting Information). Although *G. hirsutum* averages 60% more regions of introgression per accession, the average total length of introgressed segments is only 31% longer in *G. hirsutum*, potentially indicating fewer but longer regions of introgression in *G. barbadense* (Note S5 and Table S7, Supporting Information) (Table 3).

In *G. barbadense*, introgression was greatest in the cultivated accessions, averaging 6.8 Mb per cultivar (Table 3). Notably, this is higher than the average per accession introgression in cultivated *G. hirsutum* (4.4 Mb). Introgression in the genic space of the *G. barbadense* landrace populations was much lower (0.9–1.7 genes per accession). Consistent with the generally lower amount of introgression in *G. barbadense*, fewer genes show evidence of introgression in at least one accession (3973 out of 65 636, vs 4421 in *G. hirsutum*; Note S5 and Table S5, Supporting Information). Each gene exhibited introgression in fewer accessions (1.3 vs 6.6; Note S5 and Table S5, Supporting Information) and occurring in 10% of accessions at most (vs 42% in *G. hirsutum*; Note S5 and Table S8, Supporting Information). Genic introgression was highest in the cultivars, which had an average of 8.8 genes introgressed per chromosome per accession; in contrast, the landraces only averaged ≈2–3 introgressed genes (Note S5 and Table S8, Supporting Information).

As in *G. hirsutum*, introgression in *G. barbadense* D_T_ chromosomes was more abundant than in A_T_ chromosomes, both with respect to the average number of regions (14.9 A_T_ vs 24.5 D_T_; Note S5 and Table S6, Supporting Information) and average length (1.9 Mb A_T_ vs 2.1 Mb D_T_). The sole exception is Landrace 1, where the average number of regions was equivalent for A_T_ and D_T_ and the average length was greater for A_T_ chromosomes (Table 3). Interestingly, Tanguis cotton generally exhibits more introgression than the rest of Landrace 1, with threefold more regions (on average) and nearly ten times the average length, which may indicate a history of repeated (and possibly intentional) introgression in these currently cultivated landrace accessions.

Consistent with the overall higher level of introgression in the D_T_ subgenome, the number of D_T_ genes introgressed in at least one accession was 30% higher than A_T_ genes (2285 D_T_ vs 1688 A_T_); however, the average number of accessions that share an introgressed gene was equivalent between A_T_ and D_T_ (1.3). Of the 208 genes in *G. barbadense* regions putatively under selection (Note S5 and Table S5, Supporting Information), only 10 (5%) were also located in introgressed regions, possibly indicating that introgression has not played a big part in the breeding history of these accessions.

## 3 Discussion

Humans are largely dependent on only several dozen plant species, each resulting from a long history of strong directional selection and giving rise to common domestication phenotypes, including reduced seed dormancy, increased yield, changes in plant architecture associated with row‐cropping, loss of photoperiod sensitivity, and improved fruit (fiber) quality. Accordingly, there has long been an interest in characterizing the evolutionary processes associated with domestication and crop improvement, including describing patterns of variation and crop origins, the shape and severity of genetic bottlenecks, diffusion pathways across the landscape over time, the genetic basis of crop traits, and the role of interspecific introgression.^[^ ^11^, ^12^, ^52^, ^53^, ^54^, ^55^ ^]^ In recent years profound insights into these and related questions have emerged for many kinds of crop plants through the application of genomic tools, particularly whole‐genome resequencing efforts (e.g.,^[^ ^15^, ^16^, ^17^, ^18^, ^19^, ^56^, ^57^ ^]^). Here we use this approach to study allopolyploid (AD‐genome) cotton, a clade that includes seven wild species, two of which (*G. barbadense* and *G. hirsutum*) were domesticated independently by different cultures 4000–8000 years ago in NW South America and the Yucatan Peninsula of Mexico, respectively.^[^ ^3^, ^4^, ^5^, ^6^ ^]^ Our focus was on quantifying the portion as well as the proportion of the wild genetic diversity that was captured during the bottleneck associated with initial domestication and then later during subsequent crop improvement efforts, including in modern cultivars of each species. In addition, we hoped to gain insight into the importance of interspecific gene flow that occurred as the two early domesticates became sympatric as a result of human‐mediated geographic spread as well as intentionally during later crop improvement efforts. Finally, we hoped to reveal genomic regions associated with domestication and introgression.

To accomplish these goals, we conducted broad genomic resequencing of 643 accessions spanning the wild plant‐to‐elite cultivar continuum in each of the two crop species as well as multiple representatives of the other wild species, the latter to provide phylogenetic context. A distinguishing feature of our study is that the sampling strategy focused on the inclusion of wild forms or variously primitive landrace accessions, thus enabling insight into the patterns and processes accompanying crop domestication and improvement. These data were combined with existing data for hundreds of other accessions used in previous studies^[^ ^24^, ^25^, ^26^, ^27^, ^32^, ^34^ ^]^ to generate a data set that, following quality filtering, yielded 23.0 M and 26.6 M high‐quality SNPs in *G. hirsutum* and *G. barbadense*, respectively, with these two species being distinguished by 33.8M SNPs. As detailed below, these data provide insights into A) phylogenetic relationships among and within species; B) patterns of genetic diversity and the impact of domestication; C) the amount and genomic distribution of reciprocal interspecific introgression between the two cultivated species; and D) signatures of selection accompanying crop improvement.

### 3.1 Phylogenetic Relationships

#### 3.1.1 Phylogenetic Relationships among Species

Earlier work on phylogenetic relationships among the allopolyploid cotton species showed that they had a monophyletic origin^[^ ^43^ ^]^ and that one branch of the earliest divergence is now represented by the NE Brazilian species *G. mustelinum*.^[^ ^39^ ^]^ Consistent with previous studies on a subset of these species^[^ ^30^ ^]^ or a less extensive genomic dataset,^[^ ^2^, ^35^, ^36^, ^43^ ^]^ we demonstrate here (Figure 1A) that these other six species are divided into two clades, the “barbadense” group containing *G. barbadense* and *G. darwinii*, and the “hirsutum” group, which includes the Hawaiian Islands endemic *G. tomentosum, G. hirsutum*, and two geographically restricted species that are close relatives of *G hirsutum*, that is, *G. ekmanianum* and *G. stephensii* (latter not shown in Figure 1A). These phylogenetic results are echoed in the principal component analysis (Figure 1C), which also depicts the relative scales of diversity encompassed by each taxonomic species. Finally, and for the first time, divergence among all species is quantified by genome‐wide estimates of *F*_ST_, as depicted in Figure 1D. Collectively these results provide robust insight into the origin and phylogenetic relationships of the two domesticated species *G. hirsutum* and *G. barbadense*.

#### 3.1.2 Phylogenomic Analysis of Relationships within *G. hirsutum*

Because *G. hirsutum* and *G. barbadense* were first domesticated thousands of years ago and because wild forms are relatively rare, elucidating their geographic origins and subsequent diffusion pathways have been problematic, resisting illumination for at least a century.^[^ ^40^, ^58^, ^59^ ^]^ With respect to the former species, *G. hirsutum* has an aggregate indigenous range encompassing most of the American tropics, where it occurs in a diversity of forms including wild populations, commensal or door‐yard derivatives of various stages of domestication, and feral plants that have reverted to quasi‐wild morphologies. Truly wild *G. hirsutum* populations are uncommon and are ecologically restricted to relatively undisturbed coastal habitats such as stabilized, semi‐open dune vegetation.^[^ ^40^, ^60^, ^61^, ^62^ ^]^ Most *G. hirsutum* populations at the time of European colonization were, however, located inland and associated with human activity, either as row‐crops, commensals, or ferals.^[^ ^40^, ^60^, ^63^ ^]^ These morphologically highly variable plants defy a straightforward division into clear groupings, but Hutchinson's^[^ ^63^ ^]^ classification into six domesticated landraces (‘marie‐galante’, ‘punctatum’, ‘richmondi’, ‘morrilli’, ‘palmeri’, and ‘latifolium’) and one wild race (‘yucatanense’) provided a useful first approximation of an infraspecific division. Early genetic studies identified centers of diversity in the Caribbean and in southern Mexico/Guatemala,^[^ ^3^ ^]^ which, despite complex relationships among accessions and clear indications of both pre‐ and post‐colonization germplasm exchange, suggested that the geographic origin of the modern cultivated gene pool was centered in southern Mexico/Guatemala. Morphological considerations and comprehensive genetic marker analyses,^[^ ^4^ ^]^ however, led to the hypothesis that this region was a center of secondary development following the initial domestication of wild forms on the north coast of the Yucatan Peninsula.

Against these historical observations, our data provide compelling support for this general scenario. First, phylogenetic, principal component, and STRUCTURE analyses support the recognition of four germplasm groups with distinct geographic origins, here designated Wild, Landrace 1, Landrace 2, and modern or obsolete (i.e., no longer grown) cultivars (Figure 2). Inasmuch as wild *G. hirsutum* is ecologically restricted, as noted above, and is geographically confined to the Yucatan coast and adjacent areas, we infer that domestication was followed by germplasm diffusion into a broad swath of Central America, leading to the development of the diverse assemblage of material here termed Landraces 1 and 2. It was during this period, perhaps 4000 years ago or more, that the rangy perennial wild form was annualized and transformed into something resembling a modern cotton plant. Landrace 1 accessions derive mostly from northern South America (e.g., Brazil, Venezuela, and Colombia) and the Caribbean (e.g., Haiti, Puerto Rico, Dominican Republic, and Jamaica; Figure 2). The modern crop gene pool, however, clearly emerged from the clade we term Landrace 2, composed of accessions from Mexico, Guatemala, Belize, and Honduras. Our phylogenies confirmed the distinct breeding germplasm pools in the U.S. (Western, Southwest, Eastern, and Mid‐South) and China.^[^ ^37^ ^]^

These results thus strongly support the hypothesis, advanced more than 25 years ago based on allozyme^[^ ^3^ ^]^ and RFLP^[^ ^4^ ^]^ data, that modern cotton cultivars were derived from a gene pool from the geographic region occupied by the latter accessions (i.e., Landrace 2; Figure 2B). Historical, ethnographical, and archaeological support for this conjecture has been previously reviewed.^[^ ^8^ ^]^

#### 3.1.3 Phylogenomic Analysis of Relationships within *G. barbadense*

As with *G. hirsutum*, many of the details regarding the origins of modern domesticated forms of *G. barbadense* are obscure. The oldest archeological remains of *G. barbadense*, from coastal Peru, have been dated to at least 7800 years ago.^[^ ^64^ ^]^ Archaeological and morphological evidence is congruent with the limited genetic evidence in suggesting that original domestication occurred in northwestern South America,^[^ ^5^, ^6^, ^64^ ^]^ most likely in NW Peru or adjacent Ecuador, from where it later diffused into Argentina‐Paraguay and into eastern and northern South America east of the Andes. During this period and since, *G. barbadense* encountered primitive versions of cultivated *G. hirsutum*, particularly in a broad region encompassing much of the Caribbean, bringing these once widely disjunct species into sympatry following their independent domestication. The data gathered in the present study lend strong support to this general scenario, but provide a more quantitative and supported inference base. Phylogenetic, PCA, and STRUCTURE analyses (Figure 3) all support the spatio‐genetic‐temporal partitioning of *G. barbadense* similarly into four distinct groups, namely Cultivars, Landrace 1, Landrace 2, and Wild. As suggested by previous information, domestication is implicated as having taken place originally in NW South America, with subsequent phases of shared and independent crop improvement as the two landraces emerged from this initial gene pool. The two landrace groupings genetically mirror an overall geographical division into the west (Landrace 1) and east (Landrace 2) of the Andean mountain range, with the modern domesticated gene pool being more closely related to the latter gene pool. Within the domesticated gene pool, most modern cultivars cluster together, but also are partitioned into subgroups (i.e., Sea Island, Egyptian, and Pima) based on their history of domestication.

In summary, using deep sequencing of more than 1000 accessions of the two domesticated cotton species reveals the complex history of the pre‐ and post‐colonization parallel domestication process. Our results provide evidence for the geographical locations of initial domestication, the subsequent development of the primitively domesticated and modern gene pools, and the long history of interspecific contact that arose concomitantly with human‐mediated germplasm diffusion.

### 3.2 Genetic Diversity

Both *G. hirsutum* and *G. barbadense* are widely regarded as species with relatively restricted gene pools. This has been quantified in a comparative sense using allozymes^[^ ^3^, ^5^ ^]^ and restriction fragment length polymorphisms,^[^ ^4^ ^]^ and here is quantified for a broad sampling of accessions of both species using whole‐genome SNP data. Our results demonstrate among accessions on average, one might expect an SNP only about once every 500 bases (*π* = 0.002 in each species), equally distributed between the A_T_ and D_T_ genomes. This low level of standing variation likely reflects the life history of both species, which arose as polyploids with low effective population size and which are self‐compatible in mostly small, isolated populations with scattered individuals. Consequent with domestication, bottlenecks further reduced diversity, such that on average SNPs are expected once per 1.2 and 0.6 kb in *G. hirsutum* and *G. barbadense*, respectively. Thus, although these data might indicate only about a twofold and 20% decrease in diversity associated with the development of modern cultivars, this reduction has taken place from relatively genetically depauperate initial conditions. Moreover, the severity of the bottleneck in both species has to some extent been counteracted by interspecific gene flow, discussed in the following section.

### 3.3 Reciprocal Introgression

As the two nascent domesticated species (*G*. *hirsutum* and *G*. *barbadense*) diffused across cultures and landscapes from their widely disjunct ancestral homes (≈3500 km apart), they became at least partially sympatric in a broad geographic swath of each species’ cultivated range, particularly in the Caribbean. This history created the opportunity for bi‐directional interspecific gene flow, noting that the two species readily form fertile hybrids and backcrosses equivalently (although see ^[^ ^47^ ^]^ for a discussion of possible ecological barriers). Previous studies have documented several aspects of this history using multiple sources of evidence,^[^ ^3^, ^4^, ^5^, ^8^, ^47^, ^59^, ^65^, ^66^, ^67^ ^]^ and it is clear that interspecific introgression occurred both naturally as well intentionally. Historical records and pedigrees demonstrate bi‐directional cross‐breeding between species for disease and fiber traits even during the past several decades.^[^ ^5^, ^66^, ^68^, ^69^, ^70^, ^71^ ^]^ Undoubtedly this intermingling of genomes has contributed to the inability to generate a stable understanding of relationships and groupings within each species.

Given this history, it is unsurprising that bi‐directional introgression is apparent in our resequencing data. Perhaps more surprising is its sheer prominence; introgression from *G. barbadense* into *G. hirsutum* was detected in about half of the 782 accessions studied, illustrating the scope and scale of this phenomenon. With *G. hirsutum, G. barbadense* SNPs were detected in 89 of the samples from Landrace 1, 111 from Landrace 2, and 309 of the modern domesticated cotton (Note S5 and Table S2, Supporting Information). Introgressed alleles were prevalent not only among accessions but throughout the genome, (Figure 4), with most accessions exhibiting introgression on over half of the 26 chromosomes (median = 17). As expected from the history insofar as it is known, and in a manner that is congruent with our phylogenomic analyses (Figures 1 and 2), the population with the greatest introgression was Landrace 1 from the Caribbean (Note S5 and Table S2, Supporting Information). The total length of introgressed regions in *G. hirsutum* averaged 184 kb per accession (Table 3), with Landrace 1 exhibiting the most introgression (7.5 Mb per accession), 1.7x greater than the amount of introgression estimated for modern cultivars (4.8 Mb) and over twice the amount estimated for Landrace 2 (3.6 Mb). Although the total length of introgression per accession was typically less than 1% of the genome, genic regions were overrepresented, with 6.7% of the gene models in *G. hirsutum* (4421 genes out of 65 636 total gene models) exhibiting evidence of introgression in at least one accession (Note S5 and Table S4, Supporting Information).

These data thus corroborate the history of recent, intentional introgression reported for breeding in *G. hirsutum* and provide evidence that this has been occurring for centuries and likely millennia prior to European colonization of the Western Hemisphere.

*Gossypium hirsutum* not only has been the recipient of introgression from *G. barbadense*, but also the reciprocal donor to the latter species. Introgression into *G. barbadense* was detected in all 143 accessions surveyed (Note S5 and Table S6, Supporting Information). As with *G. hirsutum*, introgression into *G. barbadense* was detected on every chromosome, but the overall level of introgression was greatly reduced; whereas *G. hirsutum* averaged 4.8 Mb introgressed sequence per accession, *G. barbadense* averaged 3.6 Mb per accession (Table 3). Introgression in the cultivated *G. barbadense* was markedly higher, however, averaging 6.8 Mb per accession (Table 3), higher than the reverse, that is, introgression into cultivated *G. hirsutum*. Notably, the mean length of introgressed segments in *G. barbadense* is ≈44% longer than in *G. hirsutum* (476 kb vs 332 kb in *G. hirsutum*), suggesting that introgression into *G. barbadense* may have been, on average, more recent than in the reciprocal direction. That the two species differ so much in introgression in their broad area of sympatry expands on early observations made more than 25 years ago,^[^ ^47^ ^]^ which invoked as plausible explanations differences in population structure and pollination biology.

A final surprising dimension of introgression, observed reciprocally for the two species, is the striking asymmetry in gene flow with respect to the two co‐resident genomes in each allopolyploid. Specifically, introgression in the D‐genome predominates (Table 3), particularly for genes. In *G. hirsutum*, 70% more genes introgressed from *G. barbadense* (in at least one accession) were from the D_T_ than the A_T_ genome (2788 D vs 1633 A), and more accessions shared these introgressed genes (average 7.7 accessions for D‐homoeologs vs 6.6 for A‐homoeologs). Similarly, introgression from *G. hirsutum* into *G. barbadense* D_T_ chromosomes was more abundant than in A_T_ chromosomes, both with respect to the average number of regions (14.9 A_T_ vs 24.5 D_T_; Note S5 and Table S6, Supporting Information) and average total length (1.9 Mb A_T_ vs 2.1 Mb D_T_). While in principle this might reflect biases in subgenome detection, we note that the coresident genomes exhibit only slight biases in diversity. To our knowledge, this asymmetry between genomes for interspecific introgression has no parallel in polyploid plants. Moreover, it raises the possibility that introgressed genes might provide clues into differential adaptation under human selection to the domesticated environment. Further research on the extent and effects of introgression in domesticated species will be able to shed light on this interesting phenomenon.

### 3.4 Signatures of Selection

Despite the reduced detection analytical power imposed by the limited genetic diversity, scans for signatures of selection associated with domestication and crop improvement identified 438 putatively selected regions (Figure 4) in *G. hirsutum*, representing approximately 3% (65 MB) of the genome, with slightly higher numbers (247 vs 191) and total length (35 MB vs 30 MB) of regions in the A_T_ than D_T_ genomes. Notably, there was a striking asymmetry between the two subgenomes in the number of genes included in these regions, and in the opposite direction (795 in A_T_ vs 1226 in D_T_). These results parallel those of Ma et al.^[^ ^27^ ^]^ who reported a similar asymmetry for fiber‐related genes, but differ from other results using different germplasm comparisons.^[^ ^32^ ^]^ Notably, expression levels for 157 genes in these putatively selected regions were altered by domestication (Note S4, Table S3, and Figure S3, Supporting Information), thereby providing candidates for further functional relationships to the radically altered morphology of wild versus domesticated cotton fiber. In contrast to *G. hirsutum*, regions of selection detected in the A_T_‐genome of *G. barbadense* are twice in number and total length relative to D_T_ (168 and 48.34 MB in A_T_ vs 93 and 21.54 MB in D_T_, respectively Note S4 and Table S5, Supporting Information). Thus, genomic biases in responses to selection have been quite different under parallel selection in the two species. Also, biases in selected genes, though similar in number in the two species, are themselves biased toward the A_T_ genome in *G. barbadense* (1317 A_T_ and 1010 D_T_, respectively), reiterating these differing dynamics of responses to selection. These suggestions that directional selection has affected different targets in the two species is also reflected in the small overlap in specific genes (Note S4 and Table S6, Supporting Information) as well as differing GO enrichment results for putatively selected genes in *G. hirsutum* versus *G. barbadense*.

## 4 Concluding Remarks

Here we present a robust analysis of the allopolyploid cotton clade, presenting definitive results bearing on species relationships and with a special focus on the parallel domestication process in *G. hirsutum* and *G. barbadense*. These two species provide a remarkable example of independent domestication for the same purpose, in both cases transforming wild perennial plants into annualized row crops with strikingly different morphologies from their progenitors, especially in fiber traits. Our sampling strategy emphasized the wild and semi‐domesticated gene pools, such that we could characterize geographical origins, genetic bottlenecks associated with domestication, and subsequent geographic diffusion processes as both species emerged from their narrow ancestral homes to become important plants throughout the American tropics in pre‐colonial times and later globally subsequent to European colonization. We demonstrate pervasive and genome‐wide bidirectional introgression and present a robust analysis of infraspecific relationships in each species, genetic distances among various parts of the gene pool within each, and foundational lists of introgressed genes and those targeted unknowingly during the parallel domestication process.

## 5 Experimental Section

##### Plant Material and Sequencing

Based on previous studies^[^ ^37^, ^38^ ^]^ and availability of material from Dr. Wendel's collection at Iowa State University, 643 genetically representative accessions were selected as a “core set” of tetraploid cotton, including 442 *G. hirsutum*, 182 *G. barbadense*, and 19 other tetraploid accessions (Note S1 and Table S1, Supporting Information; including Germplasm Resources Information Network (GRIN) accession numbers, where available). Seeds from each accession were planted in the greenhouse or field of Brigham Young University (BYU, Provo, Utah), Iowa State University (ISU, Ames, Iowa), and the Southern Plains Agricultural Research Center of USDA‐ARS (College Station, TX). Young leaves were collected and shipped to BYU and extracted using the Cetyl Trimethyl Ammonium Bromide (CTAB) method.^[^ ^72^ ^]^ High‐quality DNA was shipped to BGI or the BYU Sequence Center (BYUSC) for Illumina sequencing on either the X‐Ten or HiSeq 2500, respectively. Libraries were constructed using the Illumina PCR‐free method, as the PCR‐free library method has: 1) no GC content limit; 2) improved coverage across high and low GC regions; 3) no PCR‐induced bias prior to cluster generation; 4) increased maximum library insert size for increased complexity; and 5) low read‐duplication ratio relative to PCR‐based methods. All the reads were deposited in NCBI SRA with project accession no. PRJNA414461. An additional 789 samples from previously reported resequencing^[^ ^24^, ^25^, ^26^, ^34^ ^]^ were also downloaded from the SRA for possible inclusion; however, many were later dropped due to insufficient sequencing depth (see below).

##### Read Mapping and SNP Detection

Illumina reads (PE150) were trimmed and filtered using SOAPnuke v1.6.0,^[^ ^73^ ^]^ and cleaned reads were mapped to the reference genome of TM1^[^ ^74^ ^]^ using BWA‐mem v0.7.16.^[^ ^75^ ^]^ Because *G. hirsutum* is a polyploid species, only uniquely mapped reads were retained, which allowed alleles and/or homoeologs to be distinguished.^[^ ^76^ ^]^ The output of BWA‐mem was converted to a sorted Binary Alignment Map (BAM) using SAMtools v1.7.^[^ ^77^ ^]^ Only those reads with high mapping quality against the reference genome (‐F 4 ‐q 30) were kept. Following mapping, PCR duplicates were removed from each accession with Picard v1.123 (https://github.com/broadinstitute/picard/). Then, the RealignerTargetCreator algorithm from GATK v3.5^[^ ^72^ ^]^ was used to identify candidate insertion/deletion (InDel) regions, and IndelRealigner was subsequently used to correct local misalignments.^[^ ^72^ ^]^ Indels identified by GATK were retained for further analysis and the realigned BAM files were used to call SNPs.

SNPs were identified by two independent SNP callers, GATK and FreeBayes v1.1.0,^[^ ^78^ ^]^ and the intersection of these were considered high‐confidence SNPs. The enormous size of the dataset required that the genome be divided into 500kb segments for most analyses. GATK was run as per the Best Practices for genomic resequencing.^[^ ^79^ ^]^ For FreeBayes, putative SNPs were required to have a minimum of 10X coverage containing 3 or more reads supporting that variant, a base quality of at least 20, and a mapping quality greater than 30 (‐m 30 ‐q 20 –min‐coverage 10 ‐C 3).  Consensus SNPs from both programs were generated in GATK using “SelectVariants”, and the quality value of each SNP was recalibrated (“BaseRecalibrator” from GATK) to minimize false‐positives. Only bi‐allelic SNP were retained. HaplotypeCaller from GATK was used to generate a genomic variant call format file (gVCF) for each sample, which was used for joint genotyping among all samples (via “GenotypeGVCFs” from GATK). Raw SNPs were filtered with “QD < 2.0 || FS > 60.0 || MQ <40.0 || MQRankSum < −12.5 || ReadPosRankSum < −8.0 ”, and then all 500kb segments were merged together to calculate the number of sites with missing data (per sample). Nearly 30% of individuals (408) had a missing site rate greater than 25%, and were subsequently removed from further analyses (Note S2 and Figure S3, Supporting Information); most of these samples were derived from previously generated, lower coverage data downloaded from the SRA. After removing samples with >25% missing sites, the SNP sites were filtered from the remaining 1024 samples based on the sample missing rate (>25%) and minor allele frequency (5%). Only chromosomal SNPs were retained for downstream analyses. Preliminary annotation of variant effects was performed using SnpEff v4.3.^[^ ^80^ ^]^ All scripts are available from https://github.com/Wendellab/BYUReseq.

##### Plant Material and Polymorphism Detection

643 genetically representative accessions to sequence were selected, including 442 *G. hirsutum*, 182 *G. barbadense*, and 19 other tetraploid cottons. SNPs were identified by two independent SNP callers, GATK and FreeBayes v1.1.0, and the intersection of these were considered high‐confidence SNPs.

##### Population Genetic Analysis

PCA was performed with the smartpca program embedded in the EIGENSOFT package v7.2.0;^[^ ^81^, ^82^ ^]^ parameters are available at https://github.com/Wendellab/BYUReseq. Maximum likelihood phylogenetic reconstruction was performed by RAxML v8.1.15 with the substitution model “GTRCAT” (‐m GTRCAT ‐p 12345 ‐b 1000 ‐T 4 ‐N 1000)^[^ ^83^ ^]^ using only 4D SNPs, and results were visualized with the iTOL v5.^[^ ^84^ ^]^ While SNP‐based phylogenies were not the only approach for phylogenetic reconstruction, it is noted that the interspecific SNP‐based phylogeny here recapitulates what has been previously demonstrated by classical phylogenetic analysis and by the STRUCTURE analyses here. The variant file of 4D sites was converted to structure file format using PGDSpider v2.0.9^[^ ^85^ ^]^ with default parameters. STRUCTURE v2.3.4^[^ ^86^, ^87^ ^]^ was used to infer population clusters. Ten independent runs were performed for each K between 2 and 10, with a burn‐in period of 100 000 iterations followed by 200 000 iterations of the Markov Chain Monte Carlo algorithm. Evanno's ΔΚ was applied to identify the most probable groups (K) that best fit the data using Structure Harvester v0.6.94.^[^ ^88^ ^]^ The program CLUMPP v1.1.2^[^ ^89^ ^]^ was used to align 30 iterations of the optimal K to generate a consensus structure, and the output was visualized using DISTRUCT v1.1.^[^ ^90^ ^]^ All analyses are available at https://github.com/Wendellab/BYUReseq.

##### Genetic Diversity and Detection of Selection Sweeps

Nucleotide diversity (*π*) is a measure of genetic variation, which is defined as the average number of nucleotide differences per site between any two DNA sequences chosen randomly from the sample population.^[^ ^91^ ^]^ The level of genetic diversity was measured with VCFtools v0.1.13^[^ ^92^ ^]^ using 100‐kb windows sliding 20 kb. Fixation index (*F*_st_) is a measure of population differentiation, genetic distance, based on genetic polymorphism data.^[^ ^93^ ^]^ While typically used to describe population structure within species, recent methods have also used *F*_st_ to describe variation among species.^[^ ^94^, ^95^, ^96^, ^97^ ^]^ The *F*_st_ value was measured using VCFtools v0.1.13^[^ ^92^ ^]^ 100‐kb window with sliding steps of 20 kb. Candidate domestication‐sweep windows were identified as the top 5% genomic regions exhibiting the greatest reduction in diversity (*π*_L_/*π*_c_) values and the top 5% of regions with the greatest *F*_st_ between landrace and cultivar.

##### Detection of Introgression

Introgression was evaluated using a previously published method,^[^ ^34^ ^]^ which produces results congruent with ABBA‐BABA tests while also permitting imputation of introgressed regions. Based on the results of PCA, phylogenetic tree, and population structure, 36 accessions of wild *G. hirsutum* and 46 accessions of *G. barbadense* were used to infer SNPs that distinguish the two species. Because *G. stephensii* and *G. ekmanianum* were both very close relatives of *G. hirsutum*,^[^ ^35^, ^36^, ^43^ ^]^ representatives of these species were also used to represent the ancestral SNPs of *G. hirsutum*. Mapped reads from each species were used in conjunction with interSNP,^[^ ^98^, ^99^ ^]^ which generates a species‐specific SNP index (see Note S5, Supporting Information, for more detail). Reads were separated into “*hirsutum*” or “*barbadense*” bam files by PolyCat,^[^ ^98^ ^]^ which categorizes reads based on the SNPs present, regardless of species identity. A BED file containing position data for segments of contiguous reads (minimum size 500 bp and 10x coverage) was generated from each bam file using eflen.^[^ ^99^ ^]^ Subsequently, adjacent segments with ends closer than 30 kb were merged via awk (code available from https://github.com/Wendellab/BYUReseq) and only segments of putative introgressed sequence were retained for each species. Genes located in putative introgressed regions were identified via Bedtools v2.27.1.^[^ ^100^ ^]^ Gene and segment output was tabulated and parsed in R v3.6.3. Code for analyses is available from https://github.com/Wendellab/BYUReseq.

##### Gene Expression Analysis

RNA‐seq reads for multiple, bulked tissues were downloaded from NCBI (Project ID: PRJNA490626).^[^ ^24^ ^]^ Raw reads were cleaned by SOAPnuke v1.5.2^[^ ^73^ ^]^ and subsequently aligned to the reference TM‐1 genome^[^ ^74^ ^]^ using STAR v2.7.1a.^[^ ^101^ ^]^ Quantification of gene expression was performed with Cufflinks version v2.2.1.^[^ ^102^ ^]^ To detect tissue‐dominant or tissue‐specific expression, an enrichment test was performed with TissueEnrich.^[^ ^101^, ^103^ ^]^ Gene expression in wild and cultivated *G. hirsutum* fiber was also compared using previously generated results.^[^ ^104^, ^105^ ^]^ Expression heatmaps were drawn using the online tool ClustVis.^[^ ^104^ ^]^ Gene in putative regions of selection was cross‐referenced with fiber expression for *G. hirsutum*, and GO enrichment categories were identified for all genes in regions of selection using the R package cluster Profiler.^[^ ^106^ ^]^ Functional annotations were derived from the release on CottonGen.^[^ ^107^ ^]^

## Conflict of Interest

The authors declare no conflict of interest.

## Supporting information

Supporting Information

Supporting Figures

Supporting Tables

## Data Availability

The data that support the findings of this study are openly available in NCBI SRA at https://www.ncbi.nlm.nih.gov/sra, reference number PRJNA414461.

## References

[1] M. A. Khan , A. Wahid , M. Ahmad , M. T. Tahir , M. Ahmed , S. Ahmad , M. Hasanuzzaman , in Cotton Production and Uses: Agronomy, Crop Protection, and Postharvest Technologies (Eds: S. Ahmad , M. Hasanuzzaman ), Springer Singapore, Singapore 2020, p. 1.

[2] J. F. Wendel , C. E. Grover , in Cotton (Eds: D. D. Fang , R. G. Percy ), Agronomy Monographs, American Society of Agronomy, Inc., Crop Science Society of America, Inc., and Soil Science Society of America, Inc., Madison, WI, USA 2015, p. 25.

[3] J. F. Wendel , C. L. Brubaker , A. E. Percival , Am. J. Bot. 1992, 79, 1291.

[4] C. L. Brubaker , J. F. Wendel , Am. J. Bot. 1994, 81, 1309.

[5] R. G. Percy , J. F. Wendel , Theor. Appl. Genet. 1990, 79, 529. 24226459 10.1007/BF00226164

[6] O. T. Westengen , Z. Huamán , M. Heun , Theor. Appl. Genet. 2005, 110, 392. 15580473 10.1007/s00122-004-1850-2

[7] J. F. Wendel , C. L. Brubaker , T. Seelanan , in Physiology of Cotton (Eds: J. M. Stewart , D. M. Oosterhuis , J. J. Heitholt , J. R. Mauney ), Springer, Netherlands, Dordrecht 2010, p. 1.

[8] C. L. Brubaker , F. M. Borland , J. F. Wendel , in Cotton: Origin, History, Technology and Production (Eds: C. W. Smith , J. T. Cothren ), John Wiley & Sons, New York 1999, p. 3.

[9] W. L. Applequist , R. Cronn , J. F. Wendel , Evol. Dev. 2001, 3, 3. 11256432 10.1046/j.1525-142x.2001.00079.x

[10] B. L. Gross , J. L. Strasburg , BMC Biol. 2010, 8, 137. 21078209 10.1186/1741-7007-8-137 PMC2987764

[11] J. F. Doebley , B. S. Gaut , B. D. Smith , Cell 2006, 127, 1309. 17190597 10.1016/j.cell.2006.12.006

[12] K. M. Olsen , J. F. Wendel , Annu. Rev. Plant Biol. 2013, 64, 47. 23451788 10.1146/annurev-arplant-050312-120048

[13] B. S. Gaut , Mol. Biol. Evol. 2015, 32, 1661. 26012904 10.1093/molbev/msv105

[14] M. Brozynska , A. Furtado , R. J. Henry , Plant Biotechnol. J. 2016, 14, 1070. 26311018 10.1111/pbi.12454 PMC11389173

[15] X. Huang , X. Wei , T. Sang , Q. Zhao , Q. Feng , Y. Zhao , C. Li , C. Zhu , T. Lu , Z. Zhang , M. Li , D. Fan , Y. Guo , A. Wang , L. Wang , L. Deng , W. Li , Y. Lu , Q. Weng , K. Liu , T. Huang , T. Zhou , Y. Jing , W. Li , Z. Lin , E. S. Buckler , Q. Qian , Q.‐F. Zhang , J. Li , B. Han , Nat. Genet. 2010, 42, 961. 20972439 10.1038/ng.695

[16] X. Huang , N. Kurata , X. Wei , Z.‐X. Wang , A. Wang , Q. Zhao , Y. Zhao , K. Liu , H. Lu , W. Li , Y. Guo , Y. Lu , C. Zhou , D. Fan , Q. Weng , C. Zhu , T. Huang , L. Zhang , Y. Wang , L. Feng , H. Furuumi , T. Kubo , T. Miyabayashi , X. Yuan , Q. Xu , G. Dong , Q. Zhan , C. Li , A. Fujiyama , A. Toyoda , T. Lu , Q. Feng , Q. Qian , J. Li , B. Han , Nature 2012, 490, 497. 23034647 10.1038/nature11532 PMC7518720

[17] Q. Zhao , Q. Feng , H. Lu , Y. Li , A. Wang , Q. Tian , Q. Zhan , Y. Lu , L. Zhang , T. Huang , Y. Wang , D. Fan , Y. Zhao , Z. Wang , C. Zhou , J. Chen , C. Zhu , W. Li , Q. Weng , Q. Xu , Z.‐X. Wang , X. Wei , B. Han , X. Huang , Nat. Genet. 2018, 50, 278. 29335547 10.1038/s41588-018-0041-z

[18] M. B. Hufford , X. Xu , J. van Heerwaarden , T. Pyhäjärvi , J.‐M. Chia , R. A. Cartwright , R. J. Elshire , J. C. Glaubitz , K. E. Guill , S. M. Kaeppler , J. Lai , P. L. Morrell , L. M. Shannon , C. Song , N. M. Springer , R. A. Swanson‐Wagner , P. Tiffin , J. Wang , G. Zhang , J. Doebley , M. D. McMullen , D. Ware , E. S. Buckler , S. Yang , J. Ross‐Ibarra , Nat. Genet. 2012, 44, 808. 22660546 10.1038/ng.2309 PMC5531767

[19] Z. Zhou , Y. Jiang , Z. Wang , Z. Gou , J. Lyu , W. Li , Y. Yu , L. Shu , Y. Zhao , Y. Ma , C. Fang , Y. Shen , T. Liu , C. Li , Q. Li , M. Wu , M. Wang , Y. Wu , Y. Dong , W. Wan , X. Wang , Z. Ding , Y. Gao , H. Xiang , B. Zhu , S.‐H. Lee , W. Wang , Z. Tian , Nat. Biotechnol. 2015, 33, 408. 25643055 10.1038/nbt.3096

[20] F. Li , G. Fan , C. Lu , G. Xiao , C. Zou , R. J. Kohel , Z. Ma , H. Shang , X. Ma , J. Wu , X. Liang , G. Huang , R. G. Percy , K. Liu , W. Yang , W. Chen , X. Du , C. Shi , Y. Yuan , W. Ye , X. Liu , X. Zhang , W. Liu , H. Wei , S. Wei , G. Huang , X. Zhang , S. Zhu , H. Zhang , F. Sun , X. Wang , J. Liang , J. Wang , Q. He , L. Huang , J. Wang , J. Cui , G. Song , K. Wang , X. Xu , J. Z. Yu , Y. Zhu , S. Yu , Nat. Biotechnol. 2015, 33, 524. 25893780 10.1038/nbt.3208

[21] X. Liu , B. Zhao , H.‐J. Zheng , Y. Hu , G. Lu , C.‐Q. Yang , J.‐D. Chen , J.‐J. Chen , D.‐Y. Chen , L. Zhang , Y. Zhou , L.‐J. Wang , W.‐Z. Guo , Y.‐L. Bai , J.‐X. Ruan , X.‐X. Shangguan , Y.‐B. Mao , C.‐M. Shan , J.‐P. Jiang , Y.‐Q. Zhu , L. Jin , H. Kang , S.‐T. Chen , X.‐L. He , R. Wang , Y.‐Z. Wang , J. Chen , L.‐J. Wang , S.‐T. Yu , B.‐Y. Wang , J. Wei , S.‐C. Song , X.‐Y. Lu , Z.‐C. Gao , W.‐Y. Gu , X. Deng , D. Ma , S. Wang , W.‐H. Liang , L. Fang , C.‐P. Cai , X.‐F. Zhu , B.‐L. Zhou , Z. Jeffrey Chen , S.‐H. Xu , Y.‐G. Zhang , S.‐Y. Wang , T.‐Z. Zhang , G.‐P. Zhao , X.‐Y. Chen , Sci. Rep. 2015, 5, 14139. 26420475 10.1038/srep14139 PMC4588572

[22] D. Yuan , Z. Tang , M. Wang , W. Gao , L. Tu , X. Jin , L. Chen , Y. He , L. Zhang , L. Zhu , Y. Li , Q. Liang , Z. Lin , X. Yang , N. Liu , S. Jin , Y. Lei , Y. Ding , G. Li , X. Ruan , Y. Ruan , X. Zhang , Sci. Rep. 2015, 5, 17662. 26634818 10.1038/srep17662 PMC4669482

[23] T. Zhang , Y. Hu , W. Jiang , L. Fang , X. Guan , J. Chen , J. Zhang , C. A. Saski , B. E. Scheffler , D. M. Stelly , A. M. Hulse‐Kemp , Q. Wan , B. Liu , C. Liu , S. Wang , M. Pan , Y. Wang , D. Wang , W. Ye , L. Chang , W. Zhang , Q. Song , R. C. Kirkbride , X. Chen , E. Dennis , D. J. Llewellyn , D. G. Peterson , P. Thaxton , D. C. Jones , Q. Wang , X. Xu , H. Zhang , H. Wu , L. Zhou , G. Mei , S. Chen , Y. Tian , D. Xiang , X. Li , J. Ding , Q. Zuo , L. Tao , Y. Liu , J. Li , Y. Lin , Y. Hui , Z. Cao , C. Cai , X. Zhu , Z. Jiang , B. Zhou , W. Guo , R. Li , Z. J. Chen , Nat. Biotechnol. 2015, 33, 531. 25893781 10.1038/nbt.3207

[24] L. Fang , Q. Wang , Y. Hu , Y. Jia , J. Chen , B. Liu , Z. Zhang , X. Guan , S. Chen , B. Zhou , G. Mei , J. Sun , Z. Pan , S. He , S. Xiao , W. Shi , W. Gong , J. Liu , J. Ma , C. Cai , X. Zhu , W. Guo , X. Du , T. Zhang , Nat. Genet. 2017, 49, 1089. 28581501 10.1038/ng.3887

[25] L. Fang , H. Gong , Y. Hu , C. Liu , B. Zhou , T. Huang , Y. Wang , S. Chen , D. D. Fang , X. Du , H. Chen , J. Chen , S. Wang , Q. Wang , Q. Wan , B. Liu , M. Pan , L. Chang , H. Wu , G. Mei , D. Xiang , X. Li , C. Cai , X. Zhu , Z. J. Chen , B. Han , X. Chen , W. Guo , T. Zhang , X. Huang , Genome Biol. 2017, 18, 33. 28219438 10.1186/s13059-017-1167-5 PMC5317056

[26] M. Wang , L. Tu , M. Lin , Z. Lin , P. Wang , Q. Yang , Z. Ye , C. Shen , J. Li , L. Zhang , X. Zhou , X. Nie , Z. Li , K. Guo , Y. Ma , C. Huang , S. Jin , L. Zhu , X. Yang , L. Min , D. Yuan , Q. Zhang , K. Lindsey , X. Zhang , Nat. Genet. 2017, 49, 579. 28263319 10.1038/ng.3807

[27] Z. Ma , S. He , X. Wang , J. Sun , Y. Zhang , G. Zhang , L. Wu , Z. Li , Z. Liu , G. Sun , Y. Yan , Y. Jia , J. Yang , Z. Pan , Q. Gu , X. Li , Z. Sun , P. Dai , Z. Liu , W. Gong , J. Wu , M. Wang , H. Liu , K. Feng , H. Ke , J. Wang , H. Lan , G. Wang , J. Peng , N. Wang , L. Wang , B. Pang , Z. Peng , R. Li , S. Tian , X. Du , Nat. Genet. 2018, 50, 803. 29736016 10.1038/s41588-018-0119-7

[28] Y. Hu , J. Chen , L. Fang , Z. Zhang , W. Ma , Y. Niu , L. Ju , J. Deng , T. Zhao , J. Lian , K. Baruch , D. Fang , X. Liu , Y.‐L. Ruan , M.‐U. Rahman , J. Han , K. Wang , Q. Wang , H. Wu , G. Mei , Y. Zang , Z. Han , C. Xu , W. Shen , D. Yang , Z. Si , F. Dai , L. Zou , F. Huang , Y. Bai , Y. Zhang , A. Brodt , H. Ben‐Hamo , X. Zhu , B. Zhou , X. Guan , S. Zhu , X. Chen , T. Zhang , Nat. Genet. 2019, 51, 739. 30886425 10.1038/s41588-019-0371-5

[29] M. Wang , L. Tu , D. Yuan , D. Zhu , C. Shen , J. Li , F. Liu , L. Pei , P. Wang , G. Zhao , Z. Ye , H. Huang , F. Yan , Y. Ma , L. Zhang , M. Liu , J. You , Y. Yang , Z. Liu , F. Huang , B. Li , P. Qiu , Q. Zhang , L. Zhu , S. Jin , X. Yang , L. Min , G. Li , L.‐L. Chen , H. Zheng , K. Lindsey , Z. Lin , J. A. Udall , X. Zhang , Nat. Genet. 2019, 51, 224. 30510239 10.1038/s41588-018-0282-x

[30] Z. J. Chen , A. Sreedasyam , A. Ando , Q. Song , L. M. De Santiago , A. M. Hulse‐Kemp , M. Ding , W. Ye , R. C. Kirkbride , J. Jenkins , C. Plott , J. Lovell , Y.‐M. Lin , R. Vaughn , B. Liu , S. Simpson , B. E. Scheffler , L. Wen , C. A. Saski , C. E. Grover , G. Hu , J. L. Conover , J. W. Carlson , S. Shu , L. B. Boston , M. Williams , D. G. Peterson , K. McGee , D. C. Jones , J. F. Wendel , D. M. Stelly , J. Grimwood , J. Schmutz , Nat. Genet. 2020, 52, 525. 32313247 10.1038/s41588-020-0614-5 PMC7203012

[31] G. Huang , Z. Wu , R. G. Percy , M. Bai , Y. Li , J. E. Frelichowski , J. Hu , K. Wang , J. Z. Yu , Y. Zhu , Nat. Genet. 2020, 52, 516. 32284579 10.1038/s41588-020-0607-4 PMC7203013

[32] M. F. Nazir , Y. Jia , H. Ahmed , S. He , M. S. Iqbal , Z. Sarfraz , M. Ali , C. Feng , I. Raza , G. Sun , Z. Pan , X. Du , Plants 2020, 9, 711. 10.3390/plants9060711 PMC7356552 32503111

[33] L. Li , C. Zhang , J. Huang , Q. Liu , H. Wei , H. Wang , G. Liu , L. Gu , S. Yu , Plant Biotechnol. J. 2020, 19, 109. 32652678 10.1111/pbi.13446 PMC7769233

[34] J. T. Page , Z. S. Liechty , R. H. Alexander , K. Clemons , A. M. Hulse‐Kemp , H. Ashrafi , A. Van Deynze , D. M. Stelly , J. A. Udall , PLoS Genet. 2016, 12, e1006012. 27168520 10.1371/journal.pgen.1006012 PMC4864293

[35] C. E. Grover , X. Zhu , K. K. Grupp , J. J. Jareczek , J. P. Gallagher , E. Szadkowski , J. G. Seijo , J. F. Wendel , Genet. Resour. Crop Evol. 2015, 62, 103.

[36] J. P. Gallagher , C. E. Grover , K. Rex , M. Moran , J. F. Wendel , Systematic 2017, 42, 115.

[37] P. Tyagi , M. A. Gore , D. T. Bowman , B. T. Campbell , J. A. Udall , V. Kuraparthy , Theor. Appl. Genet. 2014, 127, 283. 24170350 10.1007/s00122-013-2217-3

[38] L. L. Hinze , D. D. Fang , M. A. Gore , B. E. Scheffler , J. Z. Yu , J. Frelichowski , R. G. Percy , Theor. Appl. Genet. 2015, 128, 313. 25431191 10.1007/s00122-014-2431-7

[39] J. F. Wendel , R. Rowley , J. M. Stewart , Plant Syst. Evol. 1994, 192, 49.

[40] P. A. Fryxell , Natural History of the Cotton Tribe, 1st ed., Texas A&M University Press, College Station, Texas, USA 1979.

[41] B. Hendrix , J. M. Stewart , Ann. Bot. 2005, 95, 789. 15701660 10.1093/aob/mci078 PMC4246727

[42] J. F. Wendel , P. D. Olson , J. M. Stewart , Am. J. Bot. 1989, 76, 1795.

[43] C. E. Grover , J. P. Gallagher , J. J. Jareczek , J. T. Page , J. A. Udall , M. A. Gore , J. F. Wendel , Mol. Phylogenet. Evol. 2015, 92, 45. 26049043 10.1016/j.ympev.2015.05.023

[44] A. Wegier , A. Piñeyro‐Nelson , J. Alarcón , A. Gálvez‐Mariscal , E. R. Alvarez‐Buylla , D. Piñero , Mol. Ecol. 2011, 20, 4182. 21899621 10.1111/j.1365-294X.2011.05258.x

[45] K. Fernandez‐Stark , P. Bamber , G. Gereffi , Peru in the High Quality Cotton Textile and Apparel Global Value Chain: Opportunities for Upgrading; Duke CGGC, Duke University, Center on Globalization, Governance & Competitiveness, Durham, North Carolina, USA 2016.

[46] Y. Camán Salazar , Determinants of the production and commercialization (indirect) of Tanguis cotton in the province of Palpa, department of Ica to the state of Florida‐USA, in the period 2018 (Thesis‐partial). 2019.

[47] C. L. Brubaker , J. A. Koontz , J. F. Wendel , Am. J. Bot. 1993, 80, 1203.

[48] C. E. Grover , M.‐J. Yoo , M. Lin , M. D. Murphy , D. B. Harker , R. L. Byers , A. E. Lipka , G. Hu , D. Yuan , J. L. Conover , J. A. Udall , A. H. Paterson , M. A. Gore , J. F. Wendel , G3 2020, 10, 731. 31843806 10.1534/g3.119.400909 PMC7003101

[49] C. Shen , N. Wang , C. Huang , M. Wang , X. Zhang , Z. Lin , Plant J. 2019, 99, 494. 31002209 10.1111/tpj.14339

[50] J. I. Said , Z. Lin , X. Zhang , M. Song , J. Zhang , BMC Genomics 2013, 14, 776. 24215677 10.1186/1471-2164-14-776 PMC3830114

[51] S. Beckert , Empire of Cotton: A Global History, Vintage Books, New York City, New York, USA 2015.

[52] M. D. Purugganan , Curr. Biol. 2019, 29, R705. 31336092 10.1016/j.cub.2019.05.053

[53] B. L. Gross , K. M. Olsen , Trends Plant Sci. 2010, 15, 529. 20541451 10.1016/j.tplants.2010.05.008 PMC2939243

[54] B. S. Gaut , D. K. Seymour , Q. Liu , Y. Zhou , Nat. Plants 2018, 4, 512. 30061748 10.1038/s41477-018-0210-1

[55] R. S. Meyer , M. D. Purugganan , Nat. Rev. Genet. 2013, 14, 840. 24240513 10.1038/nrg3605

[56] H. Razifard , A. Ramos , A. L. Della Valle , C. Bodary , E. Goetz , E. J. Manser , X. Li , L. Zhang , S. Visa , D. Tieman , E. van der Knaap , A. L. Caicedo , Mol. Biol. Evol. 2020, 37, 1118. 31912142 10.1093/molbev/msz297 PMC7086179

[57] Y. Zhou , A. Muyle , B. S. Gaut , in The Grape Genome, (Eds: D. Cantu , M. A. Walker ), Springer International Publishing, Cham 2019, p. 39.

[58] S. G. Watt , The Wild and Cultivated Cotton Plants of the World: A Revision of the Genus Gossypium, Framed Primarily with the Object of Aiding Planters and Investigators Who May Contemplate the Systematic Improvement of the Cotton Staple, Longmans, Green, and Company, London, United Kingdom 1907.

[59] J. B. Hutchinson , R. A. Silow , S. G. Stephens , Q. Rev. Biol. 1949, 24, 143.

[60] G. C. d'Eeckenbrugge , J.‐M. Lacape , PLoS One 2014, 9, e107458. 25198534 10.1371/journal.pone.0107458 PMC4157874

[61] S. G. Stephens , Am. Nat. 1966, 100, 199.

[62] S. G. Stephens , Am. Nat. 1958, 92, 83.

[63] J. B. Hutchinson , Heredity 1951, 5, 161.

[64] J. C. Splitstoser , T. D. Dillehay , J. Wouters , A. Claro , Sci. Adv. 2016, 2, e1501623. 27652337 10.1126/sciadv.1501623 PMC5023320

[65] S. G. Stephens , L. L. Phillips , Biotropica 1972, 4, 49.

[66] S. G. Stephens , Agric. Hist. 1976, 50, 391.

[67] S. G. Stephens , Econ. Bot. 1976, 30, 409.

[68] G. L. Wang , J. M. Dong , A. H. Paterson , Theor. Appl. Genet. 1995, 91, 1153. 24170011 10.1007/BF00223934

[69] S. Jizhong , L. Jinlan , Z. Jinfa , Acta Gossypii Sinica 1994, 6, 135.

[70] D. D. Davis , Adv. Agron. 1979, 30, 129.

[71] P. W. Chee , A. H. Paterson , J. A. Udall , J. F. Wendel , in Polyploidy and Hybridization for Crop Improvement (Ed: A. S. Mason ), CRC Press, Boca Raton, Florida, USA 2016, p. 1.

[72] A. McKenna , M. Hanna , E. Banks , A. Sivachenko , K. Cibulskis , A. Kernytsky , K. Garimella , D. Altshuler , S. Gabriel , M. Daly , M. A. DePristo , Genome Res. 2010, 20, 1297. 20644199 10.1101/gr.107524.110 PMC2928508

[73] Y. Chen , Y. Chen , C. Shi , Z. Huang , Y. Zhang , S. Li , Y. Li , J. Ye , C. Yu , Z. Li , X. Zhang , J. Wang , H. Yang , L. Fang , Q. Chen , Gigascience 2018, 7, gix120. 10.1093/gigascience/gix120 PMC5788068 29220494

[74] C. A. Saski , B. E. Scheffler , A. M. Hulse‐Kemp , B. Liu , Q. Song , A. Ando , D. M. Stelly , J. A. Scheffler , J. Grimwood , D. C. Jones , D. G. Peterson , J. Schmutz , Z. J. Chen , Sci. Rep. 2017, 7, 15274. 29127298 10.1038/s41598-017-14885-w PMC5681701

[75] H. Li , R. Durbin , Bioinformatics 2009, 25, 1754. 19451168 10.1093/bioinformatics/btp324 PMC2705234

[76] Hu G. , Grover C. E. , Arick M. A. , Liu M. , Peterson D. G. , Wendel J. F. , Homoeologous gene expression and co‐expression network analyses and evolutionary inference in allopolyploids Brief. Bioinform. 2020, 10.1093/bib/bbaa035. PMC7986634 32219306

[77] H. Li , B. Handsaker , A. Wysoker , T. Fennell , J. Ruan , N. Homer , G. Marth , G. Abecasis , R. Durbin , Bioinformatics 2009, 25, 2078. 19505943 10.1093/bioinformatics/btp352 PMC2723002

[78] E. Garrison , G. Marth , https://arxiv.org/abs/1207.3907, https://github.com/freebayes/freebayes, 2012.

[79] G. A. Van der Auwera , M. O. Carneiro , C. Hartl , R. Poplin , G. del Angel , A. Levy‐Moonshine , T. Jordan , K. Shakir , D. Roazen , J. Thibault , E. Banks , K. V. Garimella , D. Altshuler , S. Gabriel , M. A. DePristo , Curr. Protoc. Bioinform. 2013, 43, 11.10.1. 10.1002/0471250953.bi1110s43 PMC4243306 25431634

[80] P. Cingolani , A. Platts , L. L. Wang , M. Coon , T. Nguyen , L. Wang , S. J. Land , X. Lu , D. M. Ruden , Fly 2012, 6, 80. 22728672 10.4161/fly.19695 PMC3679285

[81] K. J. Galinsky , G. Bhatia , P.‐R. Loh , S. Georgiev , S. Mukherjee , N. J. Patterson , A. L. Price , Am. J. Hum. Genet. 2016, 98, 456. 26924531 10.1016/j.ajhg.2015.12.022 PMC4827102

[82] A. L. Price , N. J. Patterson , R. M. Plenge , M. E. Weinblatt , N. A. Shadick , D. Reich , Nat. Genet. 2006, 38, 904. 16862161 10.1038/ng1847

[83] A. Stamatakis , Bioinformatics 2014, 30, 1312. 24451623 10.1093/bioinformatics/btu033 PMC3998144

[84] I. Letunic , P. Bork , Nucleic Acids Res. 2016, 44, W242. 27095192 10.1093/nar/gkw290 PMC4987883

[85] H. E. L. Lischer , L. Excoffier , Bioinformatics 2012, 28, 298. 22110245 10.1093/bioinformatics/btr642

[86] J. K. Pritchard , M. Stephens , P. Donnelly , Genetics 2000, 155, 945. 10835412 10.1093/genetics/155.2.945 PMC1461096

[87] M. J. Hubisz , D. Falush , M. Stephens , J. K. Pritchard , Mol. Ecol. Resour. 2009, 9, 1322. 21564903 10.1111/j.1755-0998.2009.02591.x PMC3518025

[88] D. A. Earl , B. M. vonHoldt , Conserv. Genet. Resour. 2012, 4, 359.

[89] M. Jakobsson , N. A. Rosenberg , Bioinformatics 2007, 23, 1801. 17485429 10.1093/bioinformatics/btm233

[90] N. A. Rosenberg , Mol. Ecol. Notes 2003, 4, 137.

[91] F. Tajima , Genetics 1983, 105, 437. 6628982 10.1093/genetics/105.2.437 PMC1202167

[92] P. Danecek , A. Auton , G. Abecasis , C. A. Albers , E. Banks , M. A. DePristo , R. E. Handsaker , G. Lunter , G. T. Marth , S. T. Sherry , G. McVean , R. Durbin , Bioinformatics 2011, 27, 2156.21653522

[93] B. S. Weir , C. C. Cockerham , Evolution 1984, 38, 1358. 28563791 10.1111/j.1558-5646.1984.tb05657.x

[94] M. B. Kantar , A. R. Nashoba , J. E. Anderson , B. K. Blackman , L. H. Rieseberg , Bioscience 2017, 67, 971.

[95] A. L. Pendleton , F. Shen , A. M. Taravella , S. Emery , K. R. Veeramah , A. R. Boyko , J. M. Kidd , BMC Biol. 2018, 16, 64. 29950181 10.1186/s12915-018-0535-2 PMC6022502

[96] J. Schmutz , P. E. McClean , S. Mamidi , G. A. Wu , S. B. Cannon , J. Grimwood , J. Jenkins , S. Shu , Q. Song , C. Chavarro , M. Torres‐Torres , V. Geffroy , S. M. Moghaddam , D. Gao , B. Abernathy , K. Barry , M. Blair , M. A. Brick , M. Chovatia , P. Gepts , D. M. Goodstein , M. Gonzales , U. Hellsten , D. L. Hyten , G. Jia , J. D. Kelly , D. Kudrna , R. Lee , M. M. S. Richard , P. N. Miklas , J. M. Osorno , J. Rodrigues , V. Thareau , C. A. Urrea , M. Wang , Y. Yu , M. Zhang , R. A. Wing , P. B. Cregan , D. S. Rokhsar , S. A. Jackson , Nat. Genet. 2014, 46, 707. 24908249 10.1038/ng.3008 PMC7048698

[97] M. Iorizzo , S. Ellison , D. Senalik , P. Zeng , P. Satapoomin , J. Huang , M. Bowman , M. Iovene , W. Sanseverino , P. Cavagnaro , M. Yildiz , A. Macko‐Podgórni , E. Moranska , E. Grzebelus , D. Grzebelus , H. Ashrafi , Z. Zheng , S. Cheng , D. Spooner , A. Van Deynze , P. Simon , Nat. Genet. 2016, 48, 657. 27158781 10.1038/ng.3565

[98] J. T. Page , A. R. Gingle , J. A. Udall , G3 2013, 3, 517. 23450226 10.1534/g3.112.005298 PMC3583458

[99] J. T. Page , Z. S. Liechty , M. D. Huynh , J. A. Udall , BMC Res. Notes 2014, 7, 829. 25421351 10.1186/1756-0500-7-829 PMC4258253

[100] A. R. Quinlan , Curr. Protoc. Bioinf. 2014, 47, 11. 10.1002/0471250953.bi1112s47 PMC4213956 25199790

[101] A. Dobin , C. A. Davis , F. Schlesinger , J. Drenkow , C. Zaleski , S. Jha , P. Batut , M. Chaisson , T. R. Gingeras , Bioinformatics 2013, 29, 15. 23104886 10.1093/bioinformatics/bts635 PMC3530905

[102] C. Trapnell , A. Roberts , L. Goff , G. Pertea , D. Kim , D. R. Kelley , H. Pimentel , S. L. Salzberg , J. L. Rinn , L. Pachter , Nat. Protoc. 2012, 7, 562. 22383036 10.1038/nprot.2012.016 PMC3334321

[103] A. Jain , G. Tuteja , Bioinformatics 2019, 35, 1966. 30346488 10.1093/bioinformatics/bty890 PMC6546155

[104] T. Metsalu , J. Vilo , Nucleic Acids Res. 2015, 43, W566. 25969447 10.1093/nar/gkv468 PMC4489295

[105] Y. Bao , G. Hu , C. E. Grover , J. Conover , D. Yuan , J. F. Wendel , Nat. Commun. 2019, 10, 5399. 31776348 10.1038/s41467-019-13386-w PMC6881400

[106] G. Yu , L.‐G. Wang , Y. Han , Q.‐Y. He , OMICS: J. Integr. Biol. 2012, 16, 284. 10.1089/omi.2011.0118 PMC3339379 22455463

[107] J. Yu , S. Jung , C.‐H. Cheng , S. P. Ficklin , T. Lee , P. Zheng , D. Jones , R. G. Percy , D. Main , Nucleic Acids Res. 2014, 42, D1229. 24203703 10.1093/nar/gkt1064 PMC3964939

