# Supplementary material for: Parallel and Intertwining Threads of Domestication in Allopolyploid Cotton
Source: Adv Sci (Weinh). 2021 Mar 15;8(10):2003634. doi: 10.1002/advs.202003634 (PMC8132148; doi:10.1002/advs.202003634)
Supplement: Supplementary file 1 — Supporting Information [file ADVS-8-2003634-s003.pdf]

© 2021 Wiley-VCH GmbH

ADVANCED SCIENCE
Open Access

Supporting Information

for *Adv. Sci.,* DOI: 10.1002/advs.202003634

Parallel and intertwining threads of domestication in allopolyploid cotton

Daojun Yuan[1,2], Corrinne E. Grover[1], Guanjing Hu[1], Mengqiao Pan[3], Emma R. Miller[1], Justin L. Conover[1], Spencer P. Hunt[4], Joshua A. Udall[5*], and Jonathan F. Wendel[1]*

Supplementary Information

Parallel and intertwining threads of domestication in allopolyploid cotton species (*Gossypium*)

Daojun Yuan[1,2*+], Corrinne E. Grover[1*], Guanjing Hu[1], Mengqiao Pan[3], Emma R. Miller[1], Justin L. Conover[1], Spencer P. Hunt[4], Joshua A. Udall[5**+], and Jonathan F. Wendel[1**]

[1]Department of Ecology, Evolution, and Organismal Biology (EEOB), Bessey Hall, Iowa State University, Ames, IA 50011, USA
[2]College of Plant Science and Technology, Huazhong Agricultural University, Wuhan, Hubei 430070, China
[3]State Key Laboratory of Crop Genetics and Germplasm Enhancement, Cotton Hybrid R & D Engineering Center, Nanjing Agricultural University, Nanjing 210095, China
[4]BioFire Inc., 515 Colorow Dr., Salt Lake City, UT 84108, USA
[5]Crop Germplasm Research Unit, USDA-ARS, College Station, TX 77845, USA
[*]authors contributed equally
[**]authors contributed equally
[+]Correspondence should be addressed to: Joshua.Udall@usda.gov or robert@mail.hzau.edu.cn

DY: 0000-0001-6007-5571 (robert@mail.hzau.edu.cn); tissue collection, DNA extraction, data generation, analysis, writing
CEG: 0000-0003-3878-5459 (corrinne@iastate.edu); analysis, writing
GH: 0000-0001-8552-7394 (hugj2006@iastate.edu); analysis, writing
MP: 0000-0002-5133-3185 (mengqiaopan@live.com); plant growth, DNA extraction, data generation
ERM: 0000-0001-9009-5303 (erdostal@iastate.edu); plant growth, DNA extraction, data generation
JLC: 0000-0002-3558-6000 (jconover@iastate.edu); tissue collection
SPH: 0000-0002-1317-4697 (spencer.p.hunt@gmail.com); DNA extraction,data generation
JAU: 0000-0003-0978-4764 (Joshua.Udall@usda.gov); project conception and funding, project management, analysis, writing
JFW: 0000-0003-2258-5081 (jfw@iastate.edu); project conception and funding, project management, writing

Keywords: Domestication, Genome evolution, Whole Genome Resequencing, Introgression, Selective sweep

Supplementary Note 1

Accessions selected for resequencing

Based on previous genetic diversity studies using multiple different techniques, we selected 643 accessions to sequence, including 442 for *G. hirsutum,* 182 for *G. barbadense,* and 19 from the remaining five tetraploid species (i.e., *G. tomentosum*, *G. mustelinum*, *G. darwinii*, *G. ekmanianum,* and *G. stephensii*).

For *G. hirsutum*, 73 accessions were chosen from the core set of cultivars used to develop the NAM breeding population and/or those containing novel SSR alleles [1]. Another 163 improved and 114 wild/feral samples were separately selected from a genetic diversity assessment of 1,523 *G. hirsutum* accessions using 105 SSR markers [2]. These two populations each represented 'a core set' of genetic diversity based on SSR markers. The domesticated and improved accessions represent the genetic diversity found in (1) the four major production zones of the U.S., (2) historical cultivars, and (3) accessions frequently present in modern pedigrees. The wild/feral *G. hirsutum* accessions include many representatives from Guatemala and the Yucatan peninsula, the latter representing truly wild forms of *G. hirsutum*. The semi-domesticated and "dooryard" accessions include all historical sub-types of *G. hirsutum*, including those referred to by the race names "marie-galante", "latifolium", "richmondii", "morrilli", "palmeri", and "punctatum". In addition, we also selected an additional 81 notable accessions from Dr. Wendel's collection at Iowa State University. In total, 232 accessions spanning the wild to landrace continuum, 189 cultivar, and 20 mutant accessions of *G. hirsutum* were sequenced (**Supplementary Note 1, Table 1)**.

For *G. barbadense*, the same set of 105 SSR markers determined clusters of genetic diversity among 410 *G. barbadense* accessions [2]. From these, we selected 182 samples to sequence, including core sets of 101 accessions spanning the wild to landrace continuum, 66 domesticated, and 15 mutant accessions. These accessions represent the diversity center/center of origin west of the Andes in coastal Peru and Ecuador, dooryard or commensal cotton from the Andes, Caribbean, and Central America, and modern domesticated lineages including Peru cotton, Egypt cotton, American Pima, and Israeli Pima cultivars.

Two to three accessions each of the remaining wild species were sequenced as representatives for SNP discovery and phylogenetic analyses. Combined with existing data, most species had more than 6 representatives, with the exception of the newly reported *G. stephensii* (2 representatives), which has limited availability.

Supplementary Note 1, Table 1. A descriptive list of the 1,432 cotton accessions analyzed in this study.

Supplementary Note 1, Fig. 1. A map depicting the location of wild/landrace (orange) or domesticated (green) *G. hirsutum* accessions. Circles scale with density of accessions. Note that location data for some samples was restricted to the country of origin (e.g., “India” or “Madagascar”). In those cases, the location was placed in the country center.

Supplementary Note 1, Fig. 2. A map depicting the location of wild/landrace (orange) or domesticated (green) cotton accessions. Circles scale with density of accessions. Note that location data for some samples was restricted to the country of origin (e.g., “India” or “Madagascar”). In those cases, the location was placed in the country center.

Supplementary Note 2

Assessment of sequence coverage, SNP detection methods, and resequencing data quality

Simulations of resequencing depth were used to assess our dataset using our variation detection pipeline. First, three replicate samples of random reads representing different coverage levels (5×, 10×, 15×, 20×, 25×, 30× and 35×) were extracted from a high coverage sequenced dataset (SRR1536366, **Supplementary Note 2 Fig. 1**). Sampled reads were independently mapped to the reference genome and analyzed for SNPs. Over 97% of reads achieved a quality score >Q20. Only uniquely-mapped, high-quality reads were retained, whereas duplicated and low mapping quality reads were removed. For the low coverage (5×) sample, which has similar depth to previous diversity assays ([3–5]; **Supplementary Note 2, Table 1**), only about 45% of the genome was covered by more than 5 reads, and only 5% was covered by more than 10, both of which reflect a general unevenness of coverage at lower sequencing depths. For the simulated 20× samples, which is comparable to the targeted coverage of this study (**Supplementary Note 2, Table 1**), more than 80% of the genome was mapped by at least 10 reads (versus 5% at 5× coverage). Because both depth and evenness of read coverage can impact the number and distribution of SNPs detected, we evaluated reproducibility of SNP detection at different coverage levels (**Supplementary Note 2 Fig. 1B**). In the 5× datasets, only ~30% of SNPs were detected in 3 out of 3 replicate analyses and only ~60% were detected in 2 out of 3 replicates. In the 20× datasets, however, 75% of SNPs were detected in all 3 replications and 86% were detected in 2 out of 3 replicates. This indicates that higher sequence coverage results in increased robustness and reproducibility of SNP detection. Methods for SNP detection can be found in the main manuscript and at https://github.com/Wendellab/BYUReseq.

We assessed coverage of all candidate datasets (1,432) (**Supplementary Note 2 Fig. 2**), and parsed these results by data origin (i.e., sequenced here or downloaded from the SRA). The effective depth of this study was 22.5x coverage, more than 4- and 10-fold than the previous reports of [6] and [7,8] . More than 85% of the reference genome had 10x coverage for samples sequenced in the present study, compared to 25% and 7% for [6] and [7,8], respectively (**Supplementary Note 2, Table 1**). After the initial round of SNP identification (see main methods), 408 of these low coverage accessions were removed from further analyses due to a significant number of missing SNPs (> 25%, **Supplementary Note 2 Fig. 3**). Notably, most of

these were from the publicly available datasets. By removing these low-coverage samples and increasing the coverage of wild/landrace accessions (**Supplementary Note 2 Fig. 3**), we were able to detect 3-fold more variation than previously reported [3,7,8].

Supplementary Note 2, Table 1. The effective coverage depth (average) and the percentage of reference genome covered at three different depths of reads.

			Covered Genome		
	Eff. Depth	Read Map. %	≥ 3 Reads %	≥ 5 Reads %	≥ 10 Reads %
Current study	22.54	86.40	89.30	87.61	85.40
Wang et al., 2017 [6]	5.45	85.82	83.28	58.35	25.21
Fang et al., 2017 [7,8]	2.24	48.57	35.05	14.36	6.90

Supplementary Note 2, Fig. 1

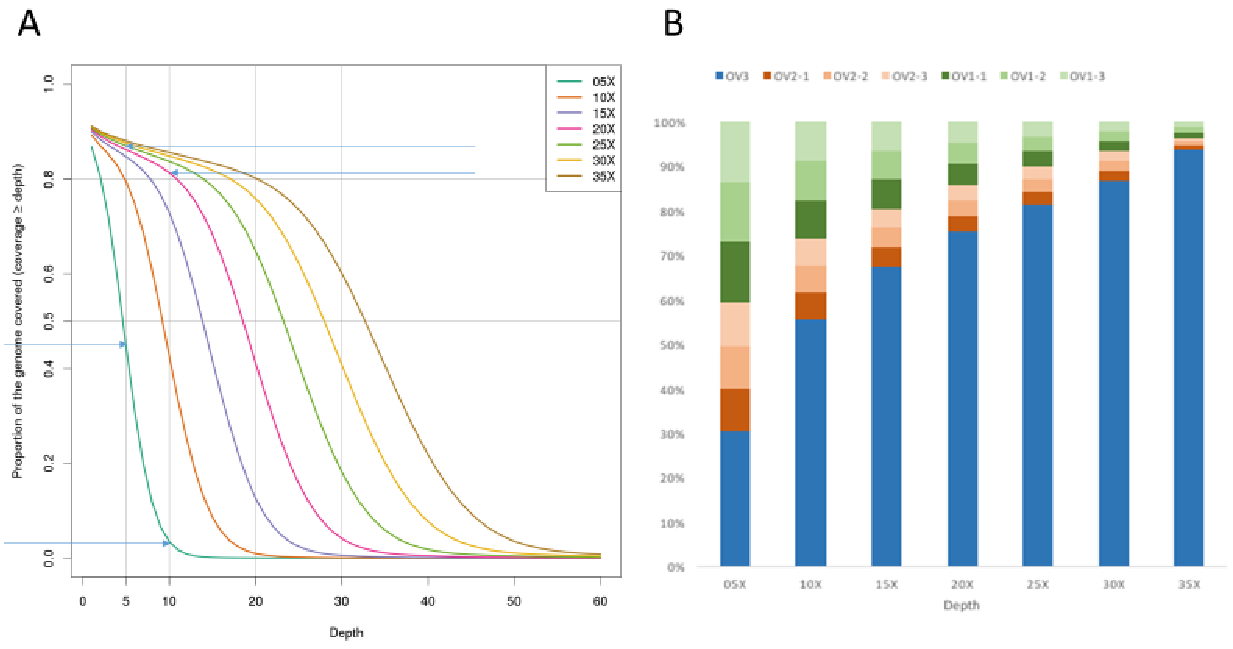

Supplementary Note 2 Fig. 1. The effect of sequencing depth on variation identification. A) Effective cumulative coverage distribution (y-axis) of different coverage datasets (x-axis). The right-pointing arrows indicate ~45% and ~5% of the reference genome that was aligned or 'covered' by more than 5 and 10 reads, respectively, in the 5× dataset. The left-pointing arrows indicate ~93% and ~80% of the reference genome that was covered by more than 5 and 10 reads, respectively, in the 20× dataset. B) The percentage of detected SNPs in different coverage datasets. OV3 = number of SNPs detected in 3 of 3 replicates, OV2-* = number of SNPs detected in 2 of 3 replicates, OV1-* = number of SNPs detected in 1 of 3 replicates.

Supplementary Note 2, Fig. 2

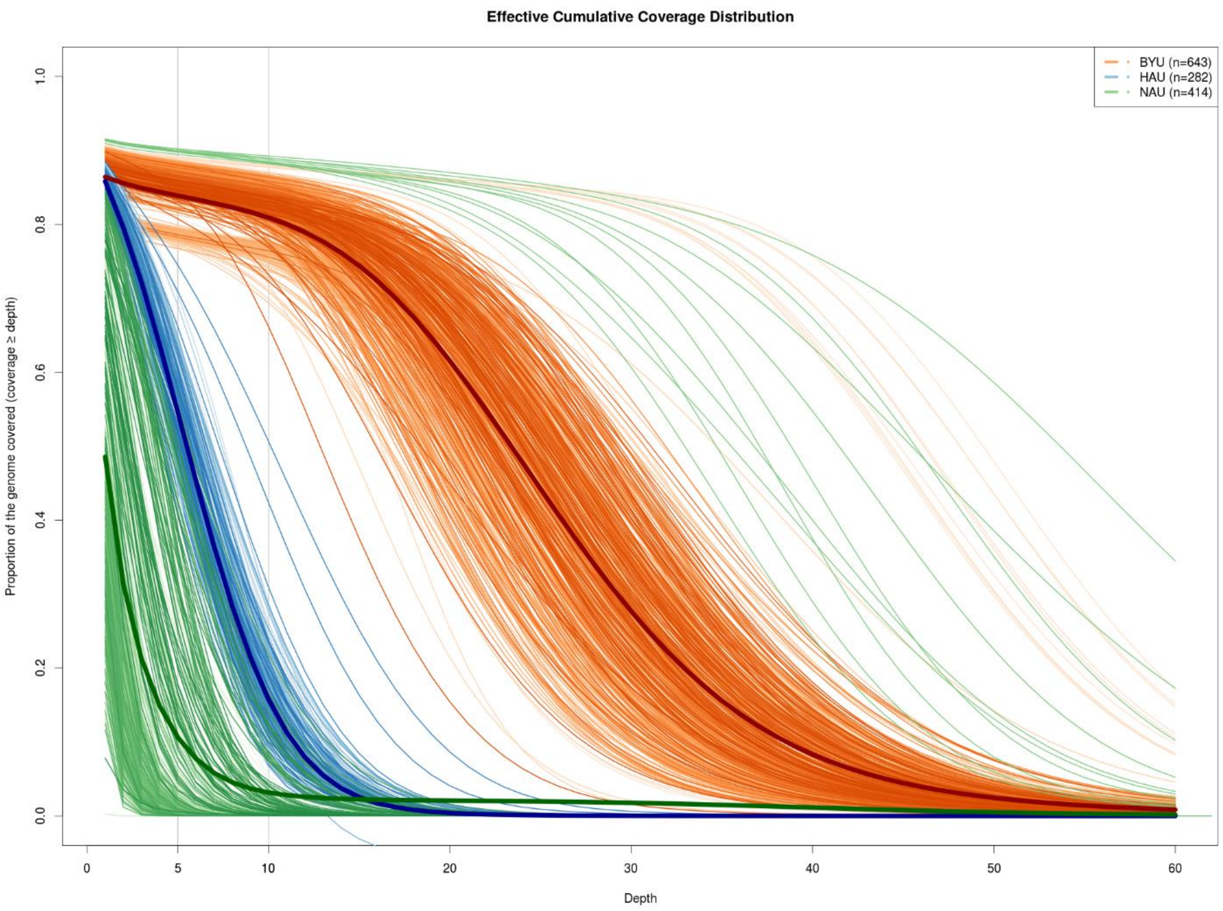

Supplementary Note 2 Fig. 2. The proportion of the reference genome was plotted against the amount of effective cumulative coverage (depth) for each accession (1,432 total accessions). The orange lines indicate the percentage of reference genome coverage (y-axis) at each coverage depth (x-axis) for individual accessions sequenced for in our current study (BYU). The dark-red slope line represents the average coverage for this study. The blue lines indicate the percentage of reference genome coverage (y-axis) at each coverage depth (x-axis) for individual accessions of the study (HAU) [6]. The dark-blue slope line represents the average coverage for that study. The green lines represent the percentage of reference genome coverage (y-axis) at each coverage depth (x-axis) for individual accessions of the study (NAU) [7,8]. The dark-green slope line represents the average coverage for that study.

Supplementary Note 2 Fig. 3

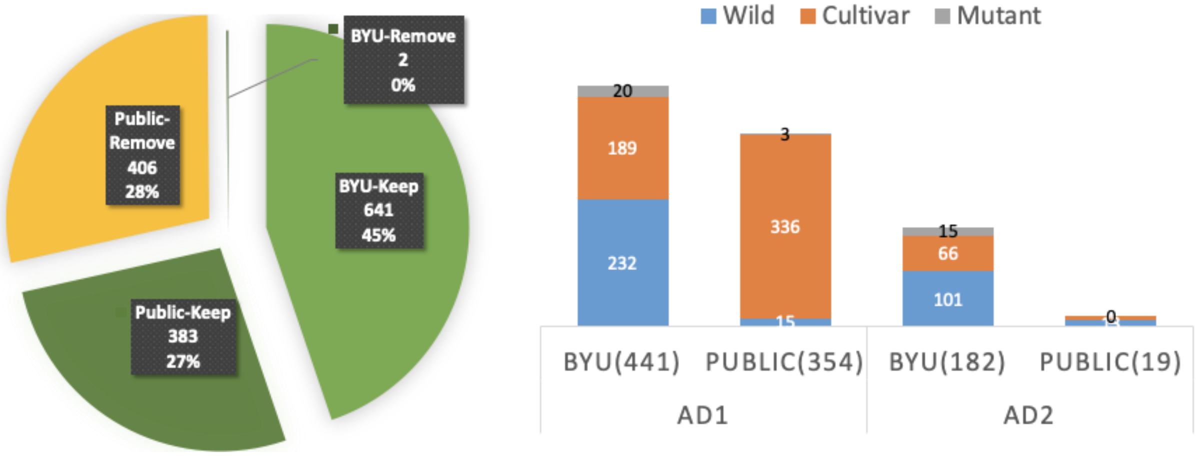

Supplementary Note 2 Fig. 3. A) Samples with >25% missing sites among the 116.2 M SNP sites led to the removal of 408 accessions from subsequent analyses. From a total of 1,432 tetraploid samples, 1,024 samples were kept for the next analysis, including 641 from this project and 383 from public data. (B) The wild/cultivar distribution of 996 *G. hirsutum* and *G. barbadense* accessions, composed of 795 *G. hirsutum* and 201 *G. barbadense* in total. Most wild/landrace accessions analyzed are newly sequenced in this study.

Supplementary Note 3

Summary of genetic variation in different genomic regions

We identified 53.7 million SNPs in the seven species of *Gossypium* (AD_1-AD_7; **Supplementary Note 3, Table 1 and Fig. 1**). Approximately half of the genic SNPs were found in introns, followed by 34.2% in coding regions, 10.1% in the 3' UTRs, and 5.7% in the 5'UTRs. In coding regions, 256,503 synonymous SNPs and 480,025 non-synonymous SNPs were identified (**Supplementary Note 3, Fig. 1**), comprising 11.9% and 22.3% of the total SNPs, respectively. We also identified 23.0 and 26.6 million SNPs within *G. hirsutum* (AD_1) and *G. barbadense* (AD_2), respectively (**Supplementary Note 3, Fig. 2)**, with a slightly higher percentage of genic SNPs was found in AD_1 than in AD_2. Genomic SNPs were used to construct a PCA to show general relationships among species and accessions (**Supplementary Note 3, Fig. 3)**

We identified 5.9 MB of InDels (<10 bp), of which 2.5 MB reflected insertions (relative to the *G. hirsutum* reference) and 3.5 MB were deletions (**Supplementary Note 3, Table 2**). Most InDels were smaller than 3 bp (**Supplementary Note 3, Table 3**). Most InDels (77.27%) were found in intergenic regions (9.79% upstream, 6.47% downstream, and 6.48% in gene regions, **Supplemental Note 3, Table 4**), which was lower than the percentage of intergenic SNPs (88.65%). In gene regions, 63.55% of the InDels were located in introns. There was a higher percentage of InDels identified upstream/downstream of genes (putative regulatory regions) compared to the percentage of identified SNPs.

Nucleotide diversity (π) was calculated for each population, as per the main methods, and diversity between *G. hirsutum* and *G. barbadense* was compared overall (**Supplementary Note 3, Fig. 4**) and with respect to genomic location (**Supplementary Note 3, Fig. 5**). Because the number of *G. hirsutum* accessions greatly outweighs the number of *G. barbadense* accessions, we verified that this imbalance did not influence our estimates of π. We calculated π for ten replicates of 100 randomly chosen vcf files from each species, and used calculated with VCFtools, as previously noted. The maximum, minimum, and average π values were calculated

for each replicate (**Supplementary Note 3, Table 5**), and these values were compared to the values recovered for the entire dataset. Fixation index (F_{ST}) is a measure of population differentiation, genetic distance, based on genetic polymorphism data [9]. We measured the F_{ST} value using VCFtools [10] in 100-kb windows with sliding steps of 20 kb (**Supplementary Note 3, Fig. 6**).

Relationships among accessions of *G. hirsutum* and *G. barbadense* were also evaluated using phylogenetics and principal component analysis using the same parameters as outlined above. In both cases, accessions of *G. mustelinum* were used as an outgroup. Both the phylogeny (**Supplementary Note 3, Fig. 7**) and PCA (**Supplementary Note 3, Fig. 8**) for *G. hirsutum* suggest a four populations that include a wild population, the modern cultivars, and two distinct landrace populations. Relationships within *G. barbadense* were likewise divided into four populations, including two distinct landraces, by phylogeny (**Supplementary Note 3, Fig. 9**) and PCA (**Supplementary Note 3, Fig. 10**). In the case of *G. barbadense*, however, several currently cultivated accessions (i.e., the Tanguis cottons of Peru) were placed within Landrace 1, consistent with the history of Tanguis cotton cultivation (see main text).

Supplementary Note 3, Table 1. Numbers of SNPs identified within and among *Gossypium* species.

Supplementary Note 3, Table 2. Numbers of Indels identified per species

	AD1	AD2	AD3	AD4	AD5	AD6	AD7
Intergenic	3,514,180	3,012,330	377,364	1,310,465	1,208,140	1,182,957	73,565
Upstream	468,084	387,592	33,817	173,094	166,278	167,363	8,247
5'UTR	30,929	25,781	2,796	11,862	11,526	11,481	806
Exon	33,203	25,899	3,396	10,669	9,242	9,523	789
Intron	194,606	160,148	17,407	74,907	68,577	70,973	5,150
3'UTR	43,537	35,739	3,667	16,873	15,684	15,884	1,214
Downstream	307,926	255,462	23,455	115,177	108,733	109,630	6,155
**Total**	**4,592,465**	**3,902,951**	**461,902**	**1,713,047**	**1,588,180**	**1,567,811**	**95,926**

Supplementary Note 3, Table 3. The length distribution of InDels relative to the *G. hirsutum* reference. Negative lengths indicate lack of sequence relative to the reference.

Length	Count	Percent(%)
-9	21,200	0.85
-8	25,525	1.03
-7	31,166	1.25
-6	49,863	2.01
-5	48,341	1.95
-4	84,451	3.4
-3	124,522	5.01
-2	355,349	14.3
-1	1,744,170	70.2
1	2,474,806	71.62
2	419,923	12.15
3	186,261	5.39
4	112,610	3.26
5	66,174	1.92
6	60,798	1.76
7	45,971	1.33
8	45,447	1.32
9	43,463	1.26

Supplementary Note 3, Table 4. Overall numbers of Indels, parsed by subgenome

Category		A_TD_T	A_T	D_T
Intergenic		4,589,773	2,773,188	1,816,585
Upstream		581,278	280,190	301,088
5'UTR		38,379	18,530	19,849
Exon		46,347	21,936	24,411
	Multiple of 3 (inframe)	13,928 (5,291)	6,440 (2,453)	7,488 (2,838)
	Other	32,419	15,496	16,923
Intron		244,482	117,560	126,922
3'UTR		55,504	26,960	28,544
Downstream		384,277	184,536	199,741
Total		5,940,040	3,422,900	2,517,140

Supplementary Note 3, Table 5. Replicate calculations of diversity using equivalent numbers of accessions

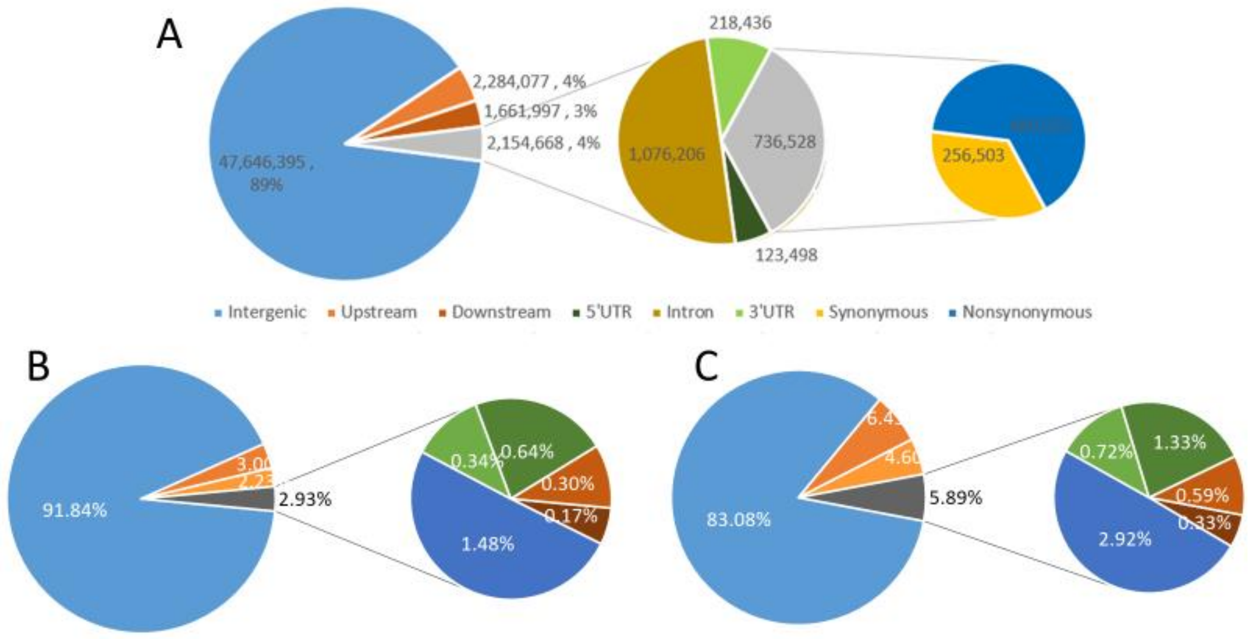

Supplementary Note 3, Fig. 1. SNP identification between seven *Gossypium* species. Identified SNPs were categorized based on their proximity to gene regions, i.e., intergenic, 2kb upstream, 2kb downstream, and genic SNPs (further categorized to 5' UTR, 3' UTR, intron, exon synonymous, and exon nonsynonymous SNPs). **A.** Overall SNPs; **B**. A_T SNPs; **C.** D_T SNPs.

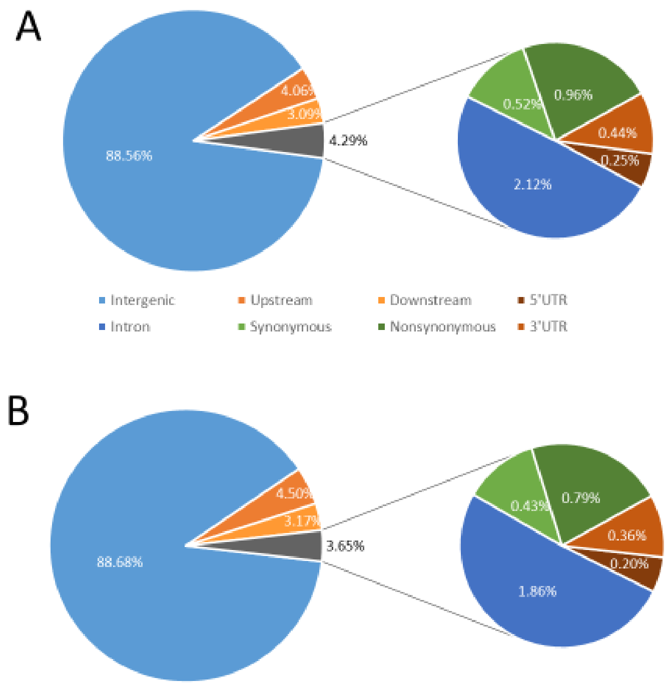

Supplementary Note 3, Fig. 2 SNP identification within *Gossypium hirsutum* (A) and within *G. barbadense* (B).

Supplementary Note 3 Fig. 3. SNP-based PCA of all samples. Sample names are labeled, and species are indicated by color and shape, as per the legend. This figure is the same as in Figure 1C, but has each individual labeled (instead of species groups).

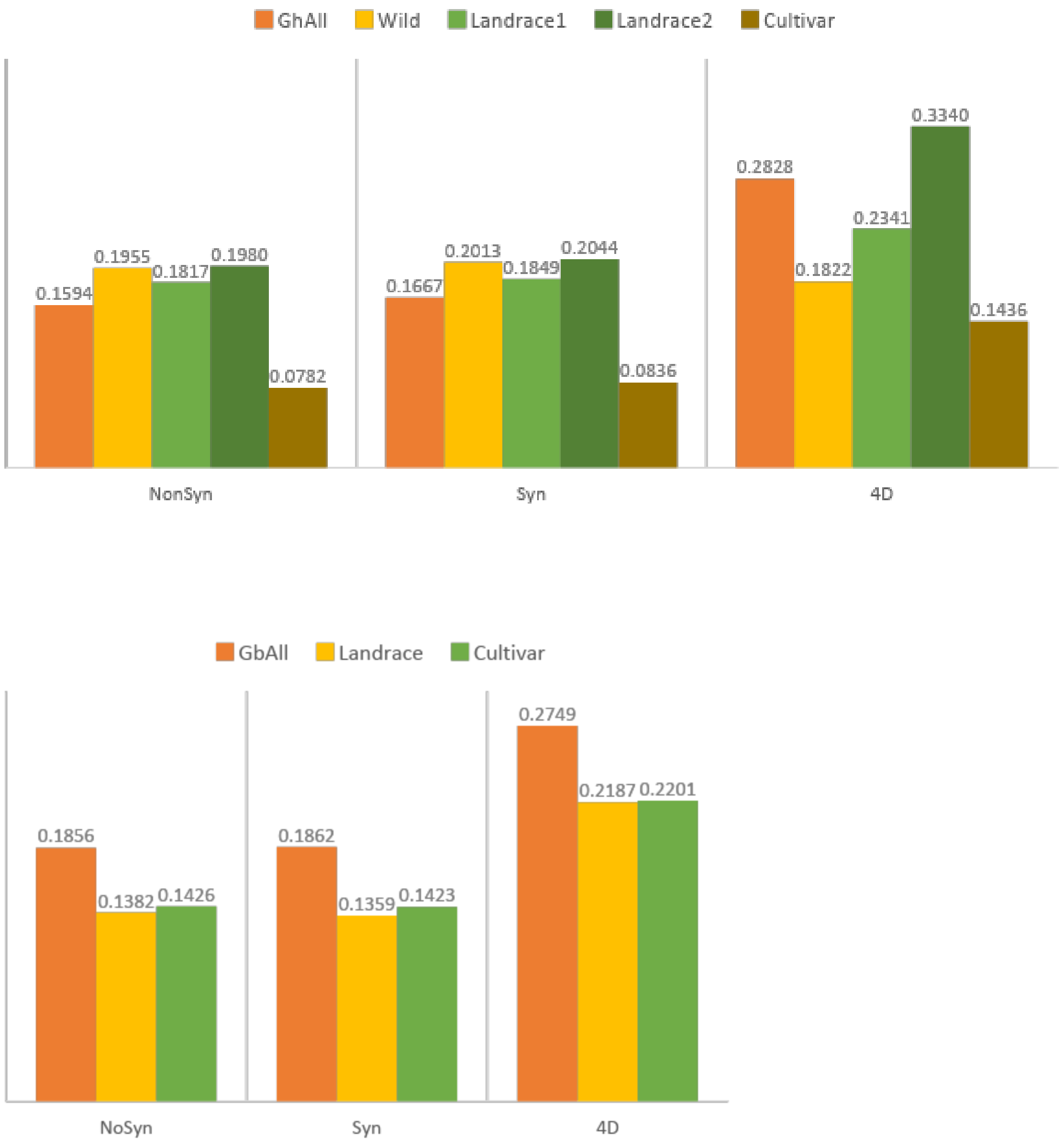

Supplementary Note 3, Fig. 4. Nucleotide diversity (π) of nonsynonymous, synonymous, and 4-fold generate sites (4D) for *G. hirsutum* (top) and *G. barbadense* (bottom), partitioned by population.

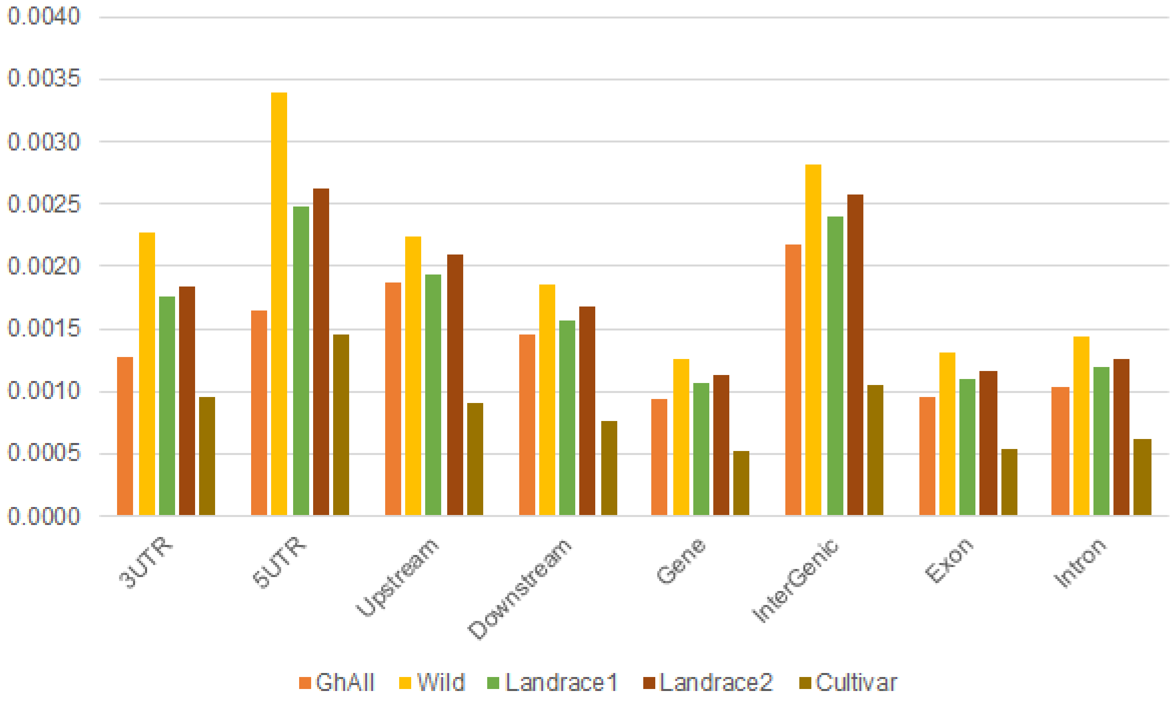

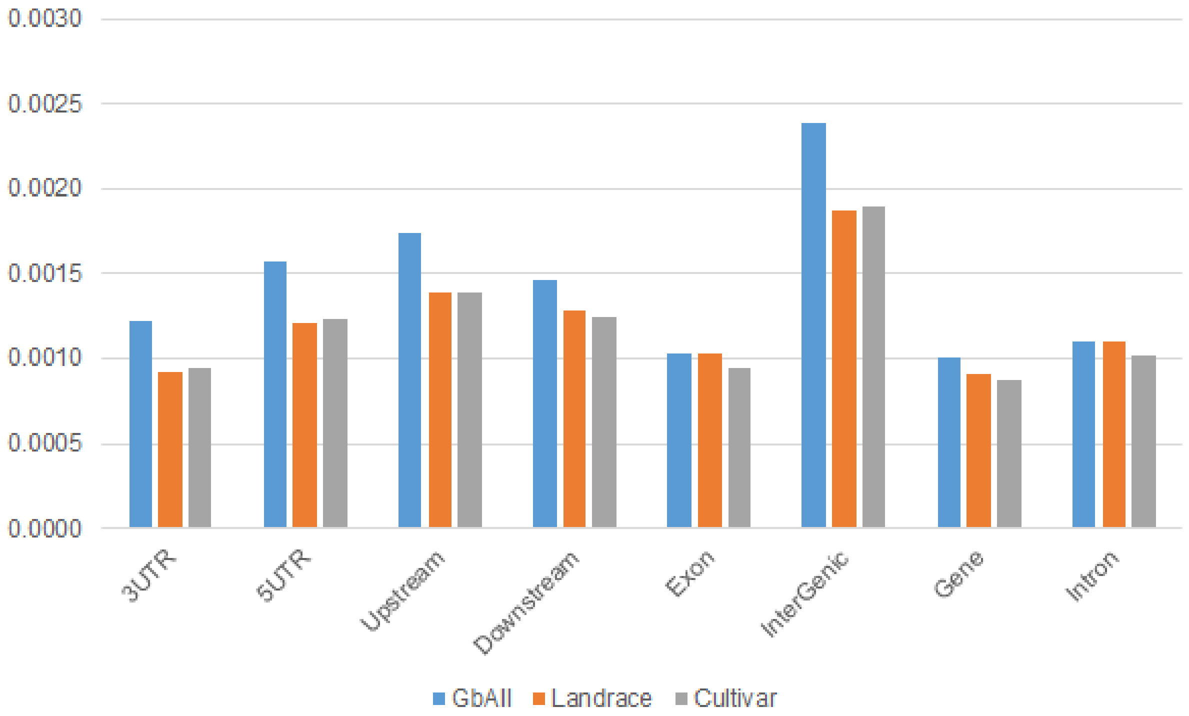

Supplemental Note 3, Fig. 5. Diversity among gene parts for *G. hirsutum* (top) and *G. barbadense* (bottom), partitioned by population.

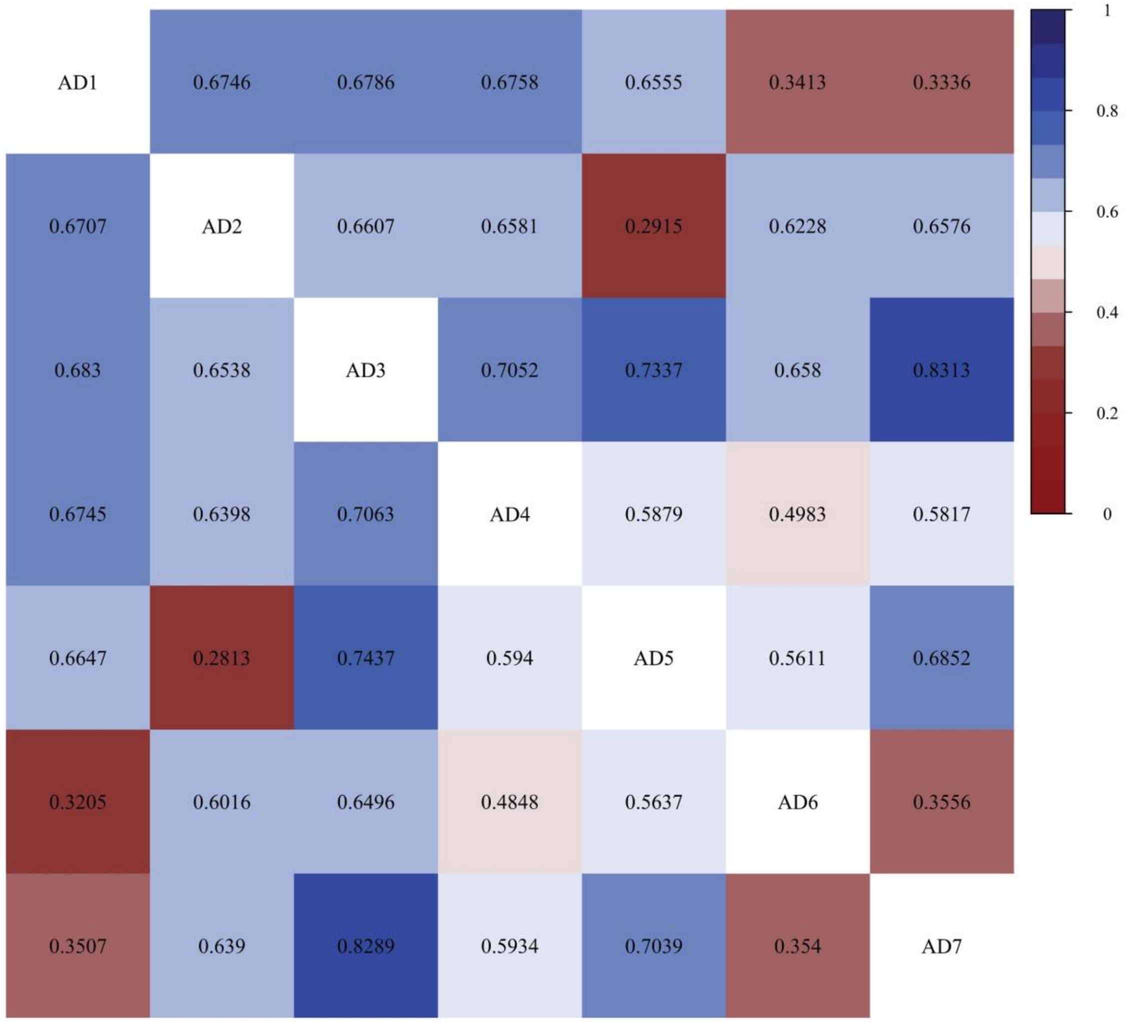

Supplemental Note 3, Fig. 6. Weighted Fst values between populations for the A (lower triangle) and D (upper triangle) subgenomes

Supplemental Note 3, Fig. 7. Phylogeny of *G. hirsutum* accessions using *G. mustelinum* as an outgroup species. Nexus formatted tree is available from https://github.com/Wendellab/BYUReseq.

Supplemental Note 3, Fig. 8. PCA of *G. hirsutum* samples, with points labeled.

Supplemental Note 3, Fig. 9. Phylogeny of *G. barbadense* accessions using *G. mustelinum* as an outgroup species. Nexus formatted tree is available from https://github.com/Wendellab/BYUReseq.

Supplemental Note 3, Fig. 10. PCA of *G. barbadense* samples, with points labeled.

Supplementary Note 4

Selective sweeps and biased expression between *G. hirsutum* and *G. barbadense*

Methods

Putative regions of selection were identified by identifying genomic regions where nucleotide diversity exhibited the greatest reduction and where F_{st} between landrace 2 and the cultivar was the greatest. As per the main methods, VCFtools v0.1.13 [10] was used to measure each in 100kb windows sliding 20kb. Candidate domestication-sweep windows were identified as the top 5% genomic regions exhibiting the greatest reduction in diversity ($\pi_L/\pi_{c[10]}$ values and the top 5% of regions with the greatest F_{st} between landrace and cultivar **(Supplementary Note 4, Fig. 1**). Putative selective sweep regions were cross-referenced with existing QTL (Grover et al. 2020; Said et al. 2013; Shen et al. 2019) for *G. hirsutum*. Enrichment for known QTL in selected regions was conducted using a binomial test to compare the proportion of QTL (length) contained within selected regions versus the whole genome. For QTL detected under domestication (Grover et al. 2020), results are significant (65.5% versus 57.7%; P<0.05). Neither the metaQTL (Said et al. 2013) nor recent QTL analyses (Shen et al. 2019) exhibit enrichment in selected regions (2.1% vs 4.3% and 11.4% vs 15.3%, respectively; $P < 0.05$); however, as both of these generally encompass selection during crop improvement (for fiber) or for cross-species comparisons, this is not unexpected. Code is available from https://github.com/Wendellab/BYUReseq.

Raw RNA-seq reads for multiple, bulked tissues were downloaded from NCBI (Project ID: PRJNA490626) [11]. These accessions include bulked RNA from roots, stems, leaves, and various reproductive organs of *G. hirsutum* accession TM-1 and *G. barbadense* accession Hai7124 [11]. Raw reads were cleaned by SOAPnuke v1.5.2 [12] and subsequently aligned to the reference TM-1 genome [13] using STAR v2.7.1a [14]. Quantification of gene expression was performed with Cufflinks version v2.2.1 [15]. To detect tissue-dominant or tissue-specific expression, we performed an enrichment test with TissueEnrich [16]. Gene expression in wild and cultivated *G. hirsutum* fiber **(Supplementary Note 4, Fig. 2**) was also compared using previously generated results [17]. Expression heatmaps were drawn using the online tool ClustVis [18]. Gene in putative regions of selection were cross-referenced with fiber expression

for *G. hirsutum* (**Supplementary Note 4, Fig. 3**), and GO enrichment categories were identified for all genes in regions of selection using the R package clusterProfiler [19] (**Supplementary Note 4, Fig. 4**). Functional annotations were derived from the release on CottonGen (https://www.cottongen.org/species/Gossypium_hirsutum/jgi-AD1_genome_v1.1).

To test whether the length of overlapping selected regions is greater than expected by chance, we performed a permutation test to randomly generate "selected regions" for *G. hirsutum* and *G. barbadense*, which maintained the number and size of regions as characterized in this dataset. Permutations were performed 1000 times to generate the null distributions of overlapped regions by region number and length. While the number of selected regions that overlap (22) was observed was within the distribution (rank in 58%), the total length of overlap (3.1 Mb) was greater than expected, occurring in less than 2.5% of the random permutations.

Supplementary Note 4, Table 1. The list of 438 putatively selected regions in *G. hirsutum*

Supplementary Note 4, Table 2. Expression profiles for 362 putatively selected genes of *G. hirsutum* that dominant expressed in ovule or fiber tissue

Supplementary Note 4, Table 3. The expression profile of 157 putative selective genes of *G. hirsutum* that have differently expressed levels between wild and domesticated cotton fiber tissue

Supplementary Note 4, Table 4. The list of 261 putatively selected regions in *G. barbadense*

Supplementary Note 4, Table 5. Selective sweeps during domestication in *G. hirsutum* and *G. barbadense*

Species	Subgenome	Number of regions	Total Length (bp)	Length relative to genome (%)	Gene Number
G. hirsutum	A_T	247	35,239,753	1.62	795
G. barbadense	A_T	168	48,339,832	2.23	1317
G. hirsutum	D_T	191	29,939,809	1.38	1227
G. barbadense	D_T	93	21,539,907	0.99	1010

Supplementary Note 4, Table 6. Genes in selected sweep regions and their functional annotation (as per www.cottongen.org/species/Gossypium_hirsutum/jgi-AD1_genome_v1.1), including genes in common between *G. hirsutum* and *G. barbadense*

Supplementary Note 4, Table 7. Expression profiles of 287 putative selective genes in *G. barbadense* upregulated in ovule or fiber tissue

Supplementary Note 4, Table 8. Expression profiles of 208 putative selective genes of *G. barbadense* that have differentially expressed levels between wild and domesticated cotton fibers

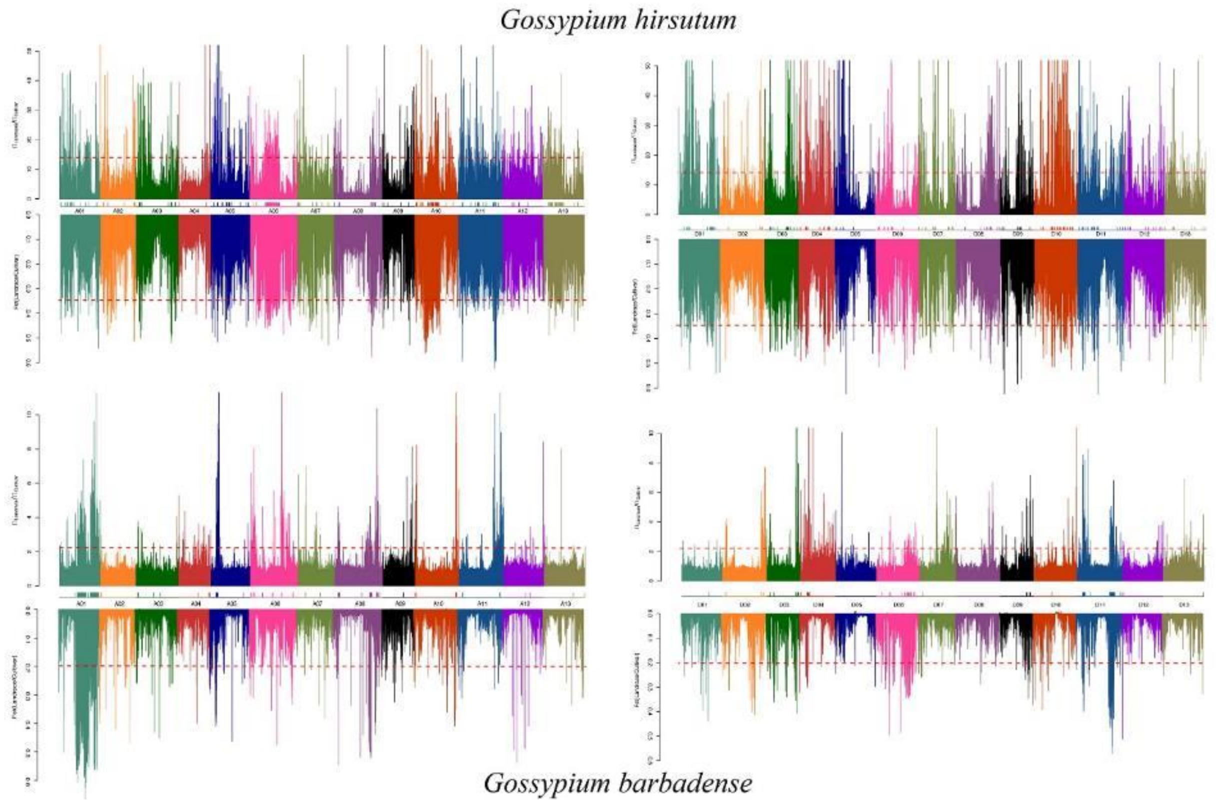

Supplementary Note 4, Fig. 1. Diversity and Fst plots detecting putative regions of selection in *G. hirsutum* (top) and *G. barbadense* (bottom) for the A_T (left) and D_T (right) chromosomes. For each panel, the top graph displays a sliding window ratio of πLandrace2/πCultivar, and the bottom panel depicts a sliding window Fst between Landrace 2 and Cultivar. For each graph, the red line indicates the 5% threshold. Between each set of graphs, is a depiction of the putative selected regions for each.

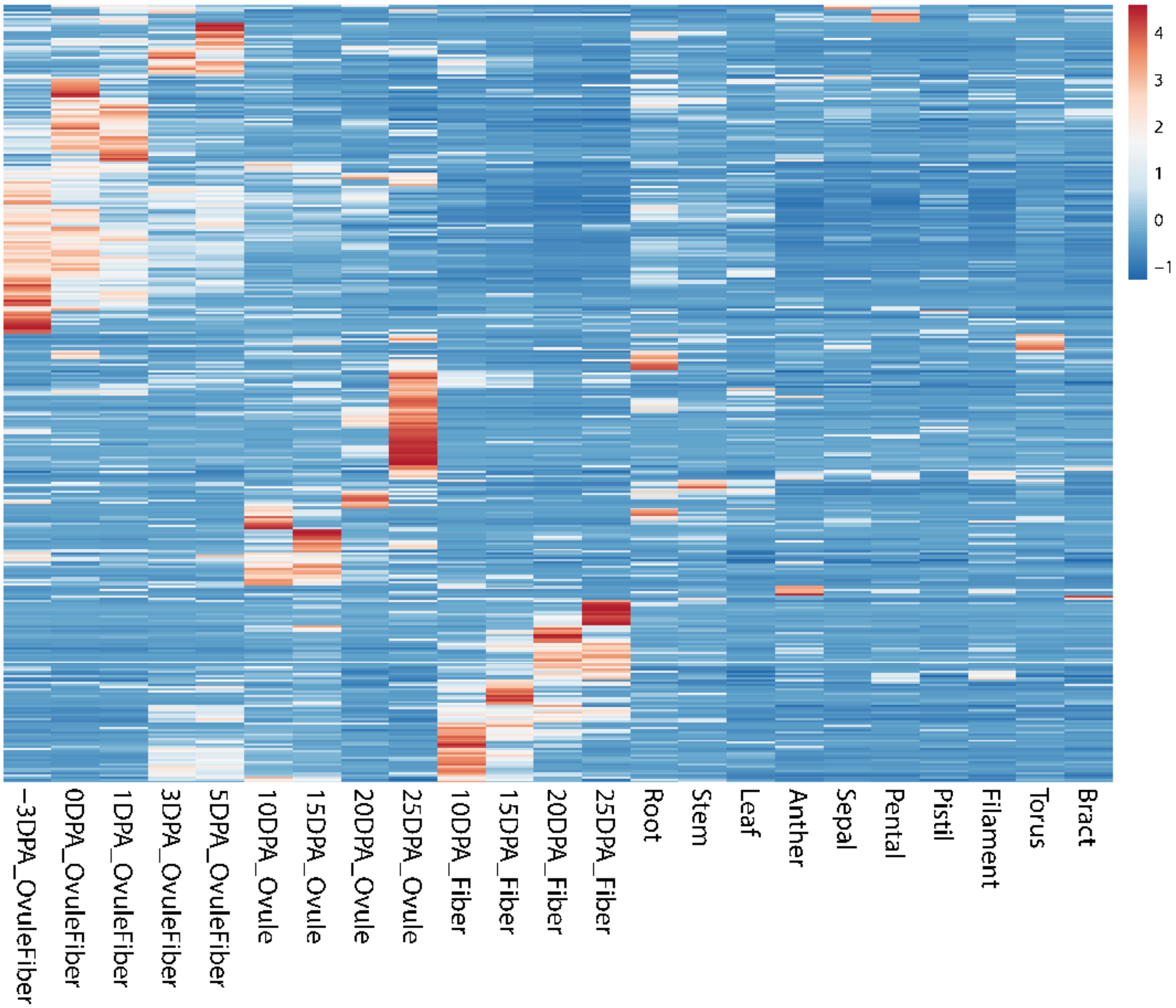

Supplementary Note 4, Fig. 2. The expression profile of 363 genes in putative regions of selection in *G. hirsutum* that are dominantly expressed in ovule or fiber tissue.

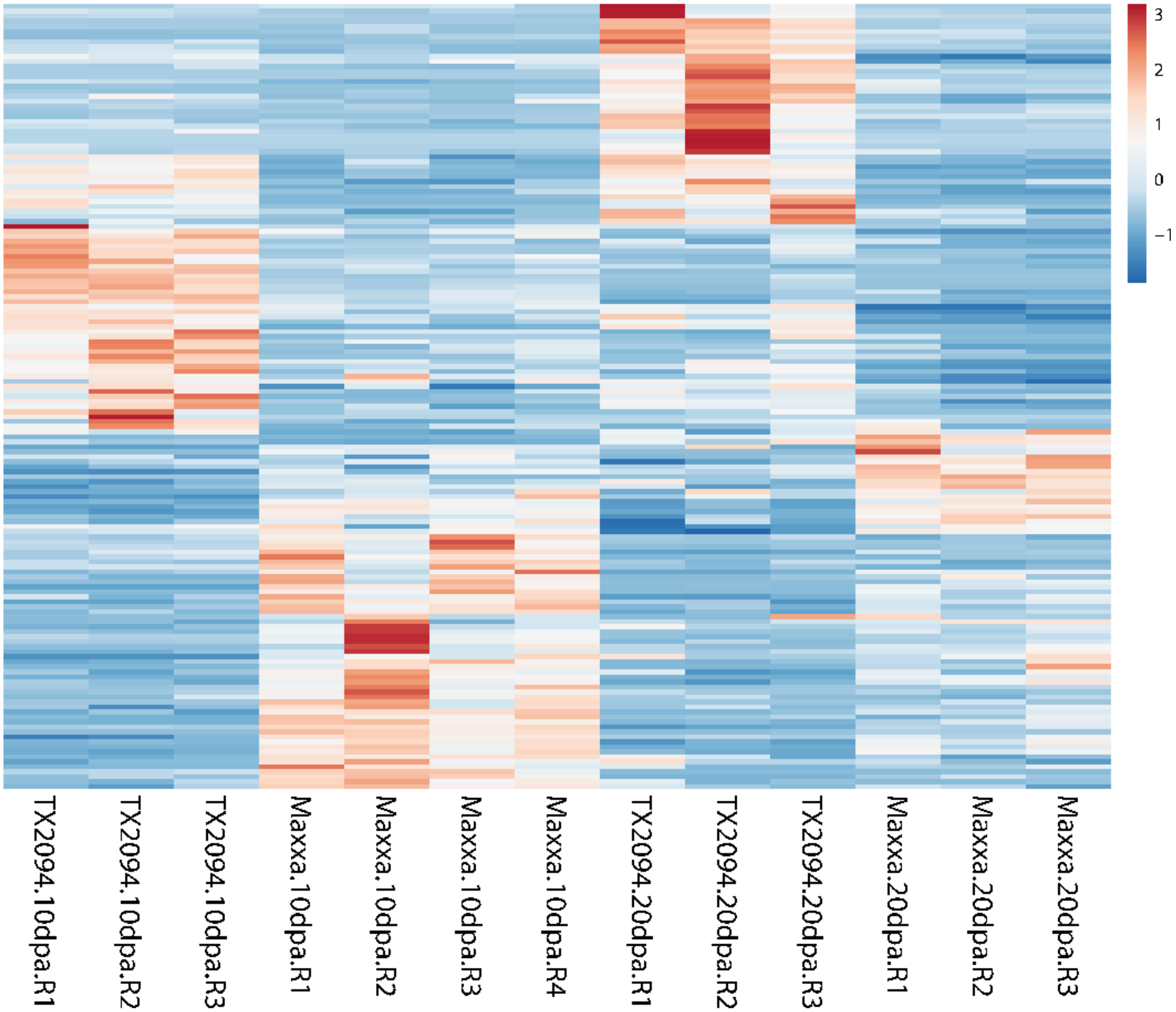

Supplementary Note 4, Fig. 3. The expression profile of 157 putative selective genes in putative regions of selection in *G. hirsutum* that have different levels of expression between wild and domesticated cotton fiber.

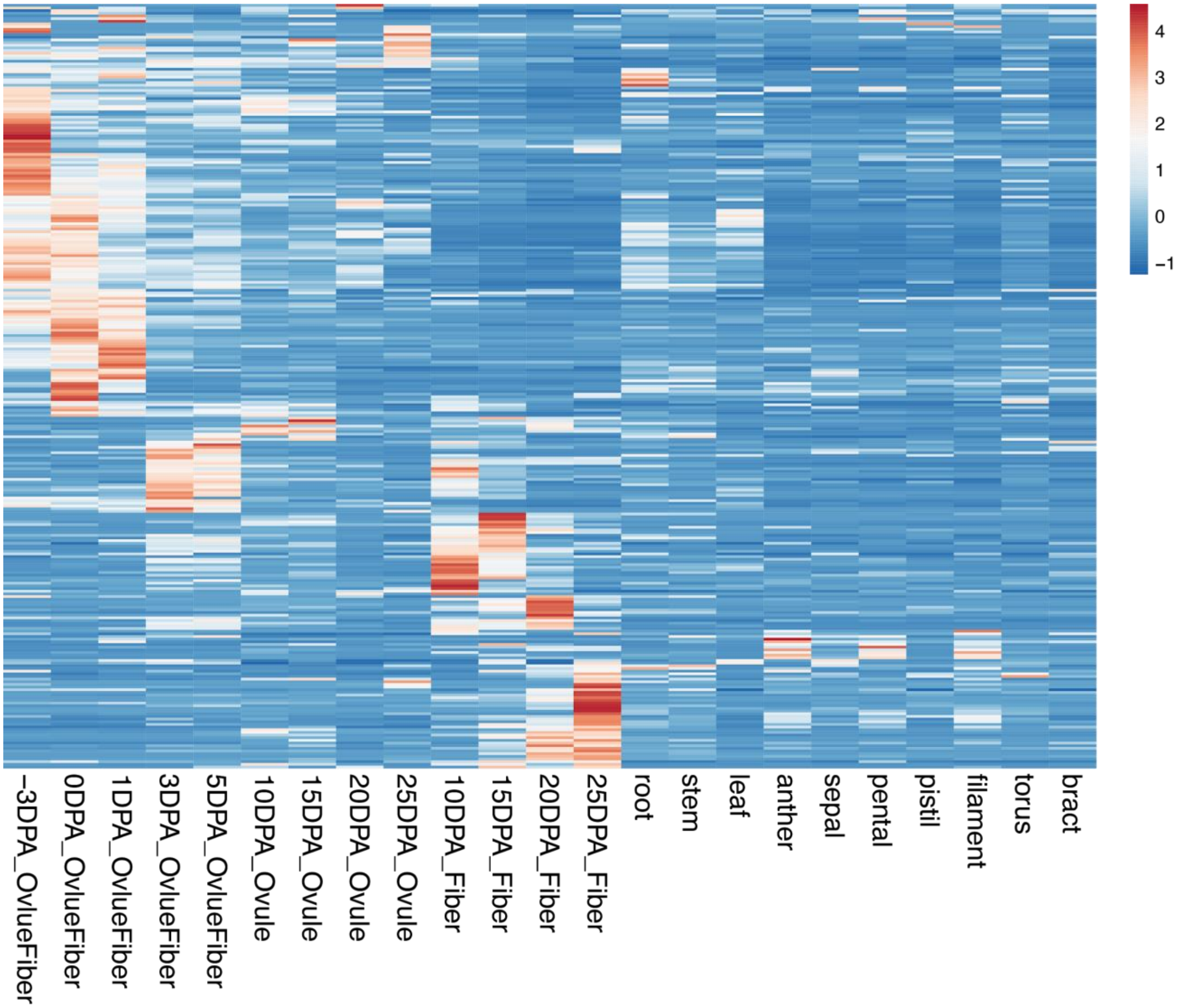

Supplementary Note 4, Fig. 4. The expression profile of 287 genes in putative regions of selection in *G. barbadense* that are dominantly expressed in ovule or fiber tissue (expression is from published *G. hirsutum* data).

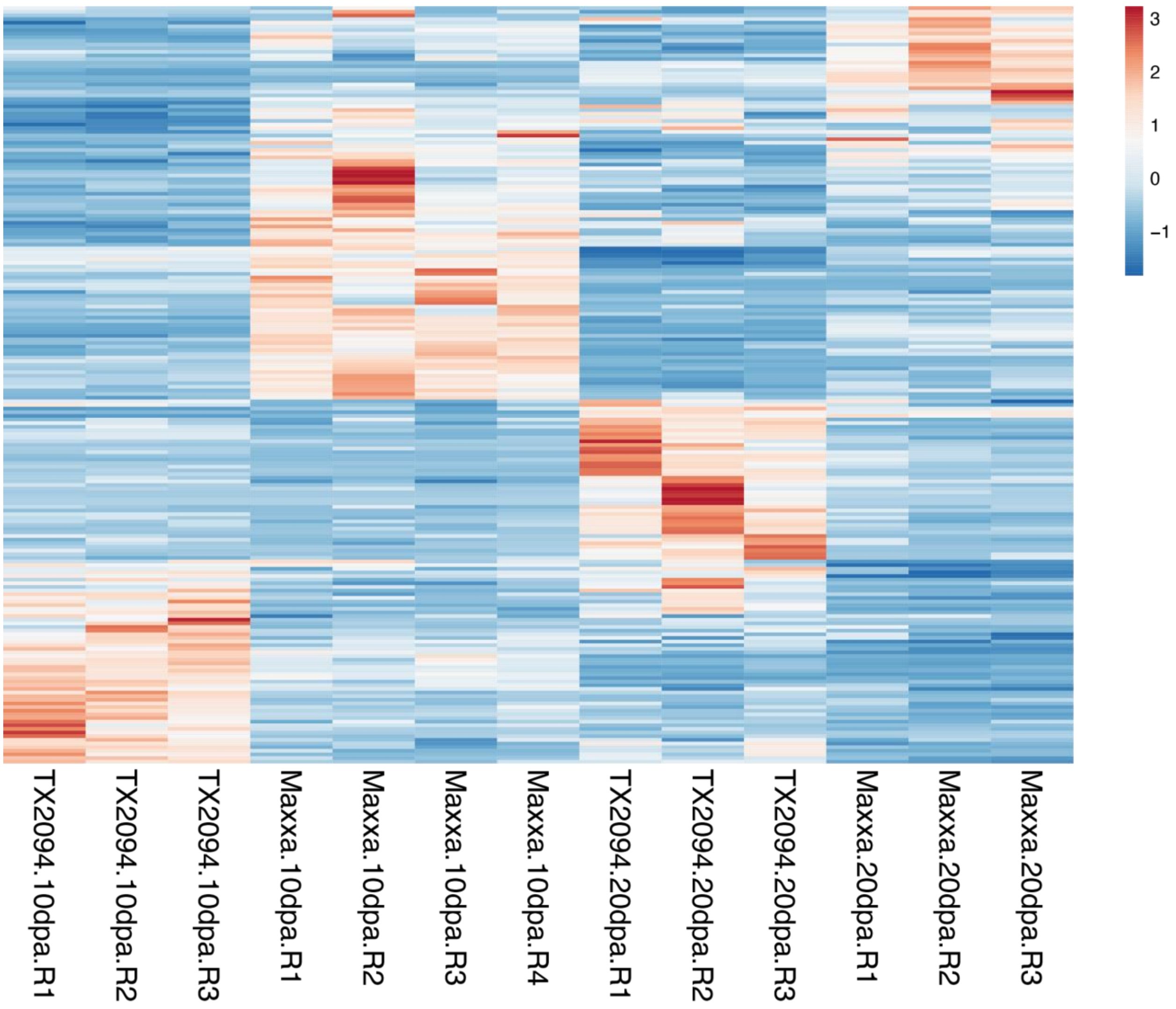

Supplementary Note 4, Fig. 5. The expression profile of 208 putative selective genes in putative regions of selection in *G. barbadense* that have different levels of expression between wild and domesticated *G. hirsutum* fiber (TX2094 is wild *G. hirsutum* and Maxxa is domesticated).

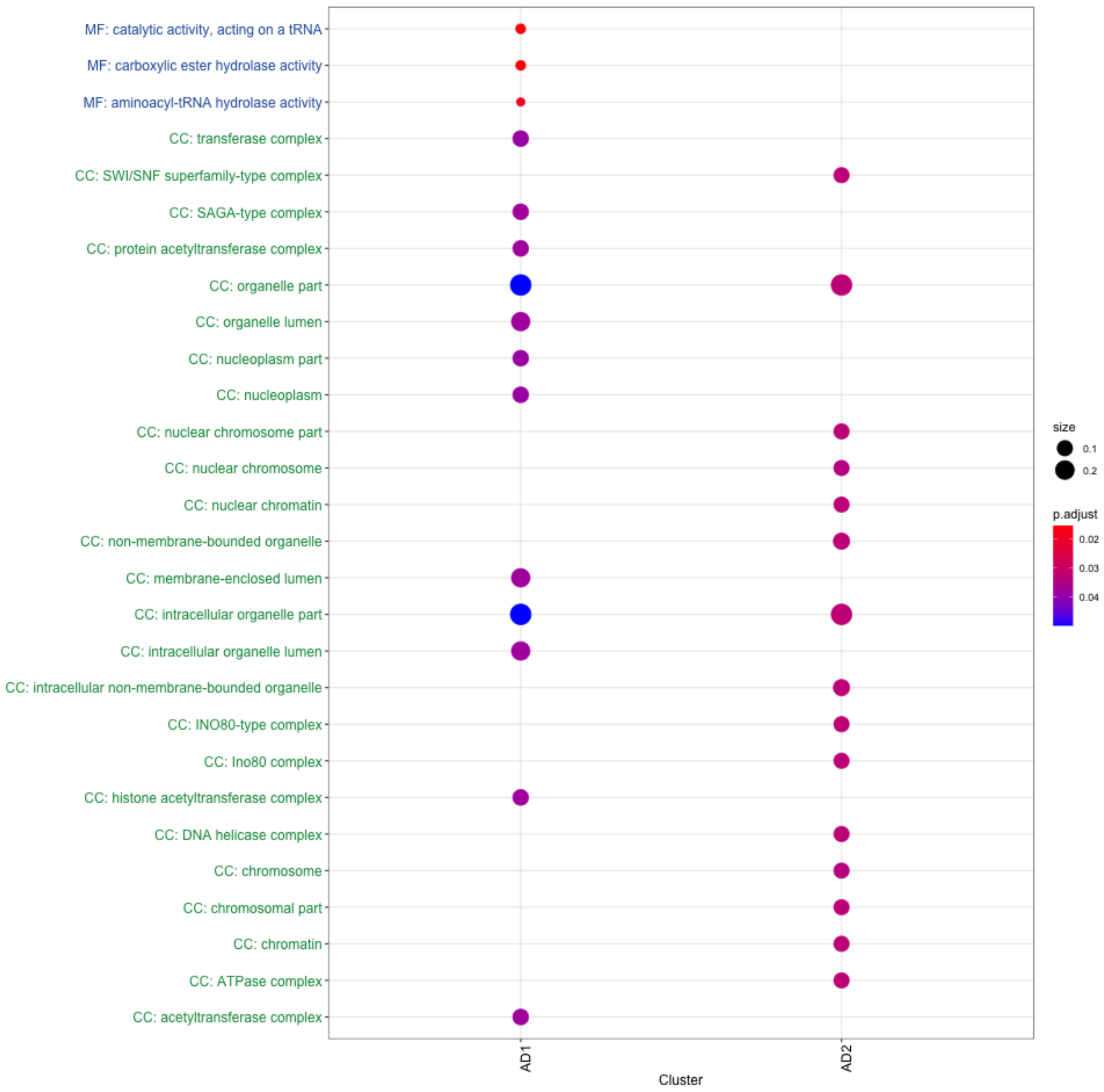

Supplementary Note 4, Fig. 6. GO enrichment analysis of selected genes in *G. hirsutum* (AD_1) and *G. barbadense* (AD_2).

Supplementary Note 5

Identification of reciprocal introgression between *G. hirsutum* and *G. barbadense*

Introgression was identified by scanning and categorizing SNPs identified between wild *G. hirsutum* and wild *G. barbadense* that did not have a history of interbreeding. Generally, we followed the methods recently described for the detection of homoeologous conversion [20]. Briefly, we created an SNP-index of nucleotides that were diagnostic of *G. hirsutum* and *G. barbadense* using multiple, truly wild accessions (36 wild *G. hirsutum* and 46 wild *G. barbadense*). Because of our depth of sequence, we used a robust 10x coverage threshold for each base throughout the pipeline. Ignoring heterozygosity, we compiled the consensus nucleotide (MAF > 0.10 and missing rate between accessions <40%) for each position and from each wild species into a joint species-specific SNP-index capable of distinguishing *G. hirsutum* SNPs from *G. barbadense*. This SNP-index was used to identify putatively introgressed bases along each chromosome by characterizing each SNP in each accession as *G. hirsutum* or *G. barbadense*-like based on the below pipeline.

Assuming that each paired-end read originated from a single DNA molecule, we scanned the read alignment file (e.g. bam file) for each accession and partitioned read pairs into separate alignment files for reads with *G. hirsutum* (H) nucleotides or reads with *G. barbadense* (B) nucleotides based on the SNP-index. For this, we used PolyCat (BamBam package, [21]), which analyzes paired-end reads concurrently and uses a default of 75% agreement among SNPs to place read pairs into a category. A second program from BamBam [21], i.e., *eflen*, was used to identify regions of introgression based on a minimum coverage (i.e., 10x) of SNPs and immediate adjacency (within 500 bp) based on read overlap, which were subsequently written as annotations (i.e., gff files). Small blocks were then merged via awk (https://github.com/Wendellab/BYUReseq) if the adjacent block was within 30 kilobases (kb) and shared the same origin (i.e., *G. hirsutum* or *G. barbadense*).

As a controlled test for introgression detection methods we used three previously reported near-isogenic lines (NIL) containing an introgressed block of *G. barbadense* into *G. hirsutum* (**Supplementary Note 5, Table 1**; [23]. These NIL lines used as controls were sequenced at much lower sequence coverage requiring a lower threshold of 3x read coverage throughout the detection pipeline. Even with lower coverage, all three homozygous, introgressed blocks were successfully identified (**Supplementary Note 5, Figures 1-12**). The small heterozygous block in N17 D08 was not detected (**Supplementary Note 5, Fig. 8**). In this region, our method detected half the coverage of 'B' bases in the introgressed region (~1.5x coverage) compared to the homozygous regions, putting the coverage level below our detection limits.

Based on the results of PCA, phylogenetic tree, and population structure, intermediate (i.e., recent hybrid) and/or misclassified samples were removed from the analysis. For *G. hirsutum*, this led to the removal of accessions DIV_055, SA-1845, SA-2606,SA-3308,TX_1249, and TX-1262; from *G. barbadense*, accessions GB_0254, GB_1449, GB_0262, GB_0631, GB-0623, olg2_14, olg2_18, olg2_5. Each accession was run individually through the pipeline indicated

above to call regions as either arising from *G. hirsutum* or *G. barbadense*. All regions that were identified as originating from the opposite species (e.g., a *G. hirsutum* region in *G. barbadense*) were retained and tabulated (Supplementary Note 5, Tables 2- 8) in R v 3.6.3 [24] using the {reshape} package [25]. All scripts are available at https://github.com/Wendellab/BYUReseq.

Supplementary Note 5, Table 1. Near-isogenic introgression lines of *G. barbadense* into *G. hirsutum* (Wang et al. 2019, Supp Table 28). Note that reference sequence of D08 used in this study and that of Wang et al. 2019 (*positions in this table) are reverse complements.

Introgression line	Chr	Start	End	Length	Type
N5	Ghir_A01	112,502,167	115,413,534	2,911,367	homo
N17	Ghir_D08	393,286 *	837,912*	444,626	hetero
N17	Ghir_A13	107,273,915	108,331,411	1,057,496	homo
N21	Ghir_D08	63,592,380*	67,824,583 *	4,232,203	homo

Supplementary Note 5, Table 2. Number of regions with inferred introgression per chromosome per accession for *G. hirsutum*. The top part of the table reflects global averages, whereas the bottom part lists accessions individually.

Supplementary Note 5, Table 3. Length of introgression per chromosome per accession for *G. hirsutum*. The top part of the table reflects global averages, whereas the bottom part lists accessions individually.

Supplementary Note 5, Table 4. Number of genes with inferred introgression per chromosome per accession for *G. hirsutum*. The top part of the table reflects global averages, whereas the bottom part lists accessions individually.

Supplementary Note 5, Table 5. Genes introgressed in either *G. hirsutum* or *G. barbadense*. The top part of the table summarizes the number of genes introgressed in common, and in each species individually, as well as the average number of accessions that share any given introgressed gene. Genes are individually listed in the bottom part of the table with their representation *G. hirsutum* or *G. barbadense* accessions. The intersection with putative genes under selection is also shown.

Supplementary Note 5, Table 6. Number of regions with inferred introgression per chromosome per accession for *G. barbadense*. The top part of the table reflects global averages, whereas the bottom part lists accessions individually.

Supplementary Note 5, Table 7. Length of introgression per chromosome per accession for *G. barbadense*. The top part of the table reflects global averages, whereas the bottom part lists accessions individually.

Supplementary Note 5, Table 8. Number of genes with inferred introgression per chromosome per accession for *G. barbadense*. The top part of the table reflects global averages, whereas the bottom part lists accessions individually.

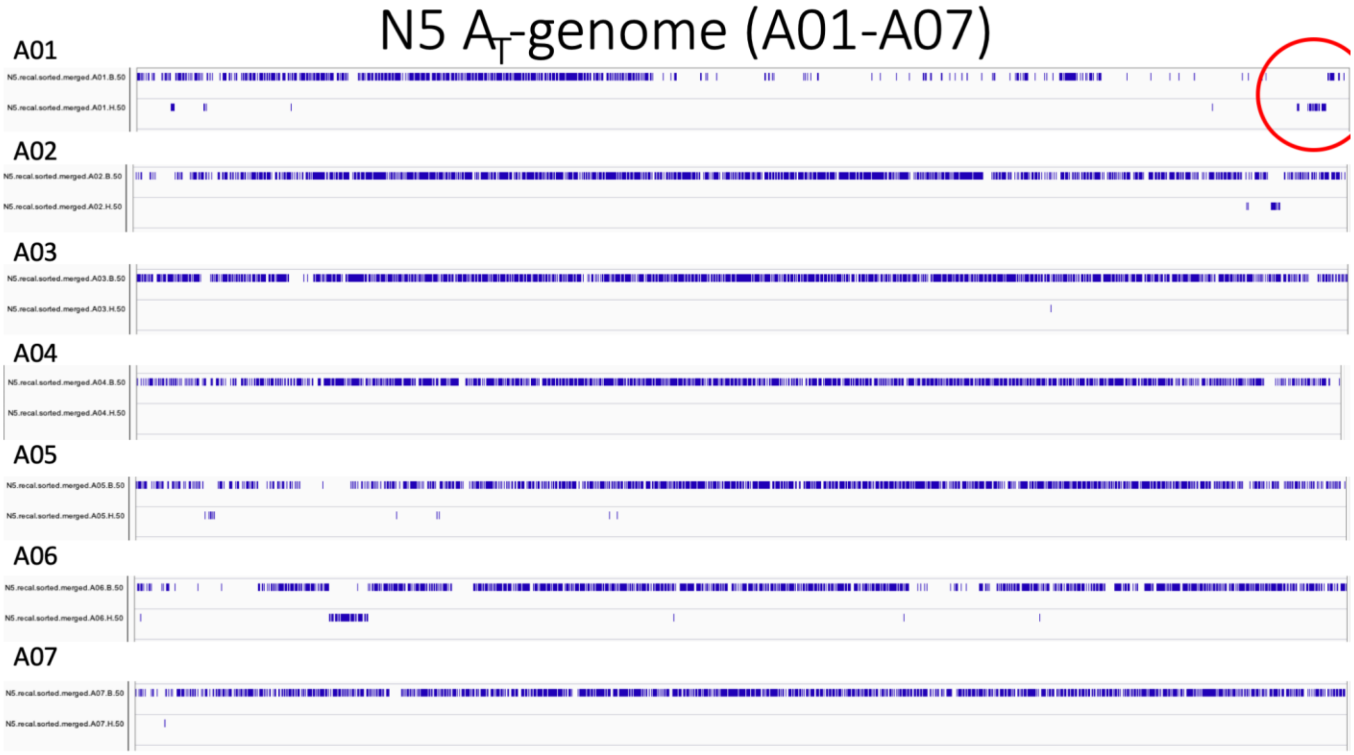

Supplementary Note 5, Fig. 1. Categorized region of chromosomes A01-A07 in *G. hirsutum* in the NIL N5 [23]. Each chromosome has two categorized tracks. The top track was categorized as 'H' for *G. hirsutum*, and the bottom track was categorized as 'B' for *G. barbadense*. A 'B' introgression region on A01 in N5 was detected at the end of chromosome A01 (red circle indicates alignments on the bottom track illustration regions categorized as 'B'. Regions of introgression were also detected on A02, A05, and A06. These additional introgressions existed in the *G. hirsutum* recurrent parent used for near isogenic line development. They are historical *G. barbadense* blocks common to nearly all accessions of *G. hirsutum* (see text). The ability to detect introgression was also dependent on the number of SNPs along the genome. Chromosome A01 had the largest number of 'holes' where the number consecutive diagnostic SNPs limited detection.

N5 A_T-genome (A08-A13)

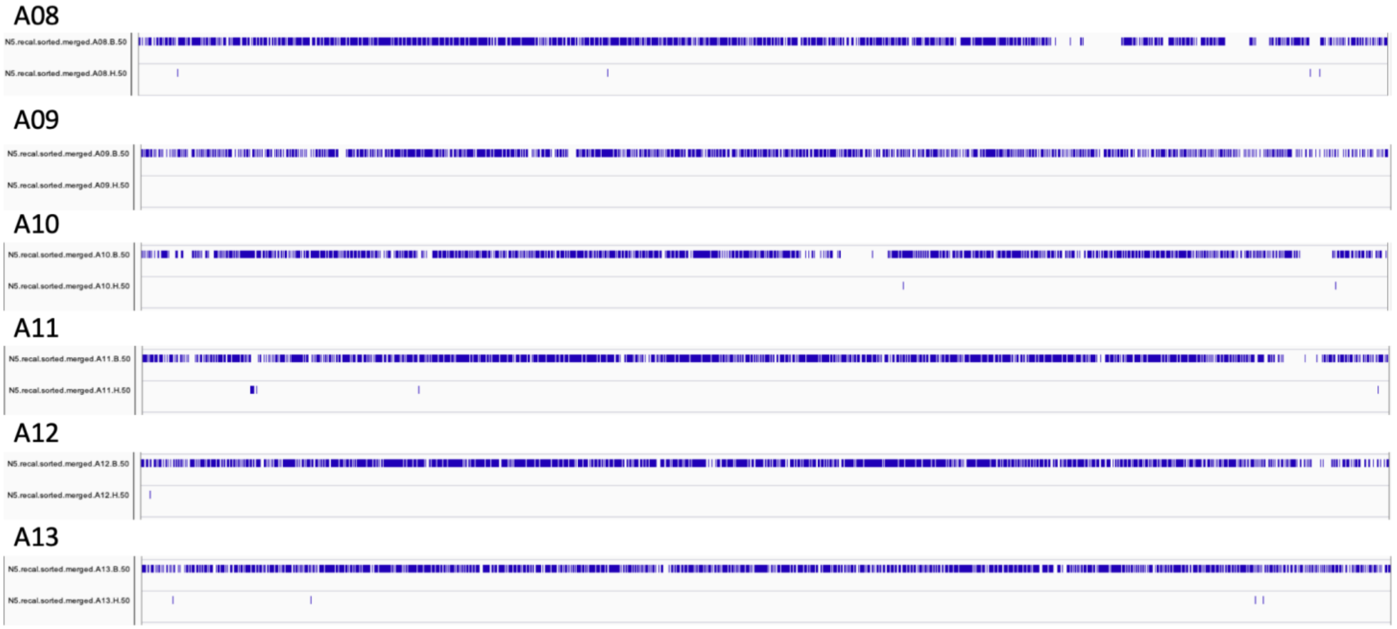

Supplementary Note 5, Fig. 2. Categorized region of chromosomes A08-A13 in *G. hirsutum* in the NIL N5 [23]. Each chromosome has two categorized tracks. The top track was categorized as 'H' for *G. hirsutum*, and the bottom track was categorized as 'B' for *G. barbadense*. No intentionally introgressed regions were detected. A region of introgression was also detected on A11. These additional introgressions existed in the *G. hirsutum* recurrent parent used for near-isogenic line development. They are historical *G. barbadense* blocks common to nearly all accessions of *G. hirsutum* (see text).

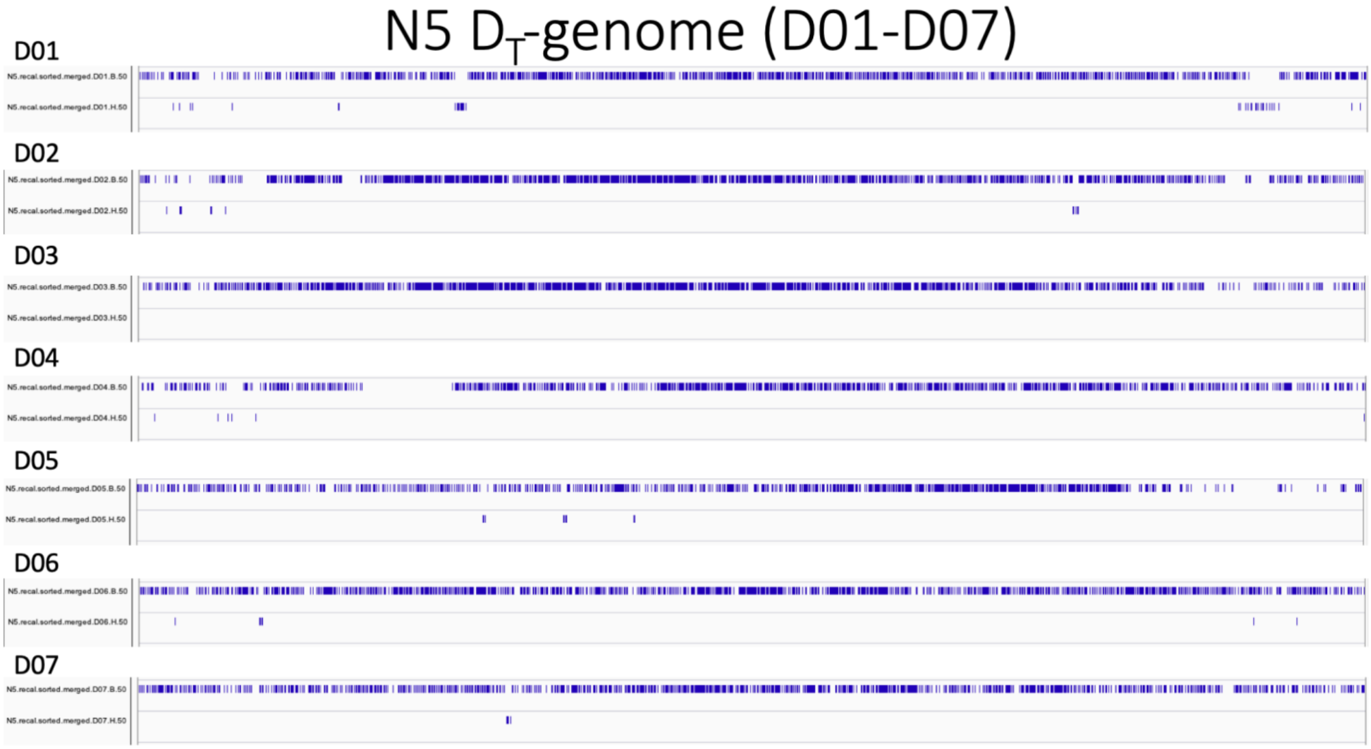

Supplementary Note 5, Fig. 3. Categorized region of chromosomes D01-D07 in *G. hirsutum* in the NIL N5 [23]. Each chromosome has two categorized tracks. The top track was categorized as 'H' for *G. hirsutum*, and the bottom track was categorized as 'B' for *G. barbadense*. No intentionally introgressed regions were detected. A region of introgression was also detected on D01, D02, D05, D06, and D07. These additional introgressions existed in the *G. hirsutum* recurrent parent used for near-isogenic line development. They are historical *G. barbadense* blocks common to nearly all accessions of *G. hirsutum* (see text).

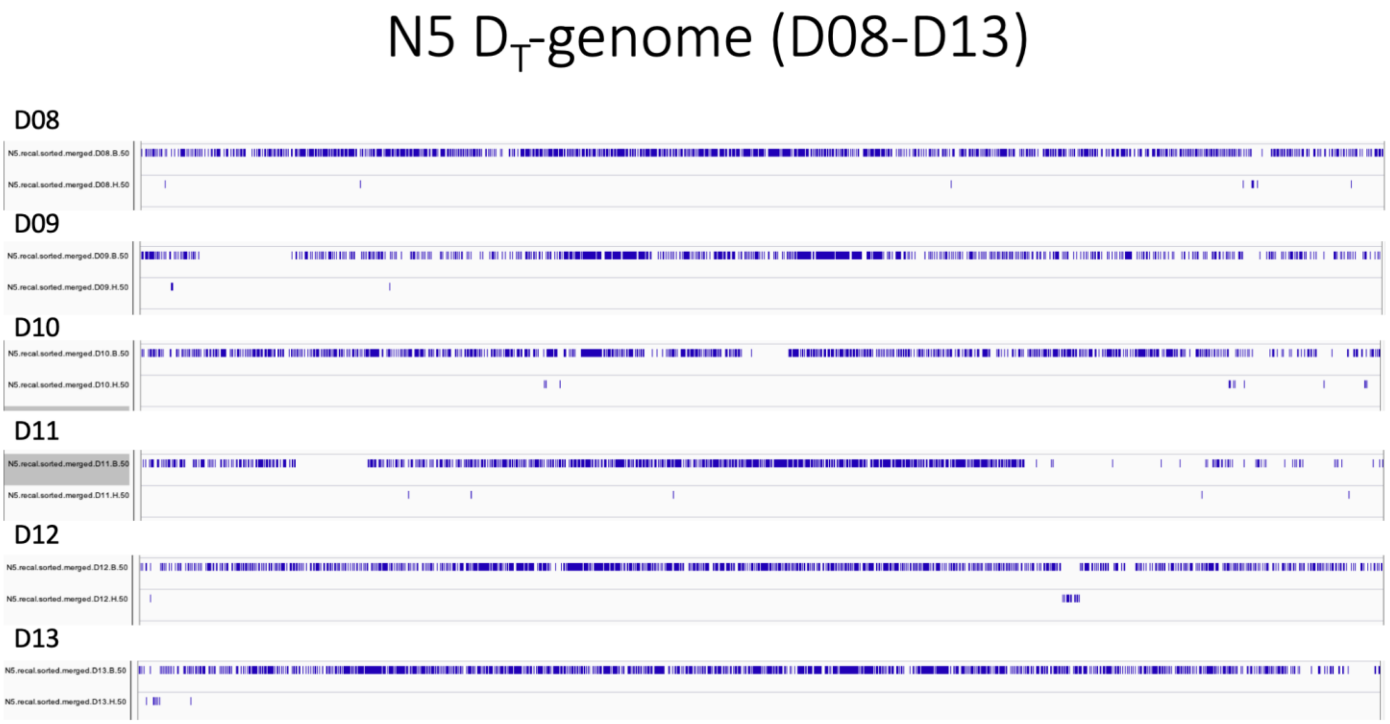

Supplementary Note 5, Fig. 4. Categorized region of chromosomes D08-D13 in *G. hirsutum* in the NIL N5 [23]. Each chromosome has two categorized tracks. The top track was categorized as 'H' for *G. hirsutum*, and the bottom track was categorized as 'B' for *G. barbadense*. No intentionally introgressed regions were detected. A region of introgression was also detected on D12. These additional introgressions existed in the *G. hirsutum* recurrent parent used for near-isogenic line development. They are historical *G. barbadense* blocks common to nearly all accessions of *G. hirsutum* (see text).

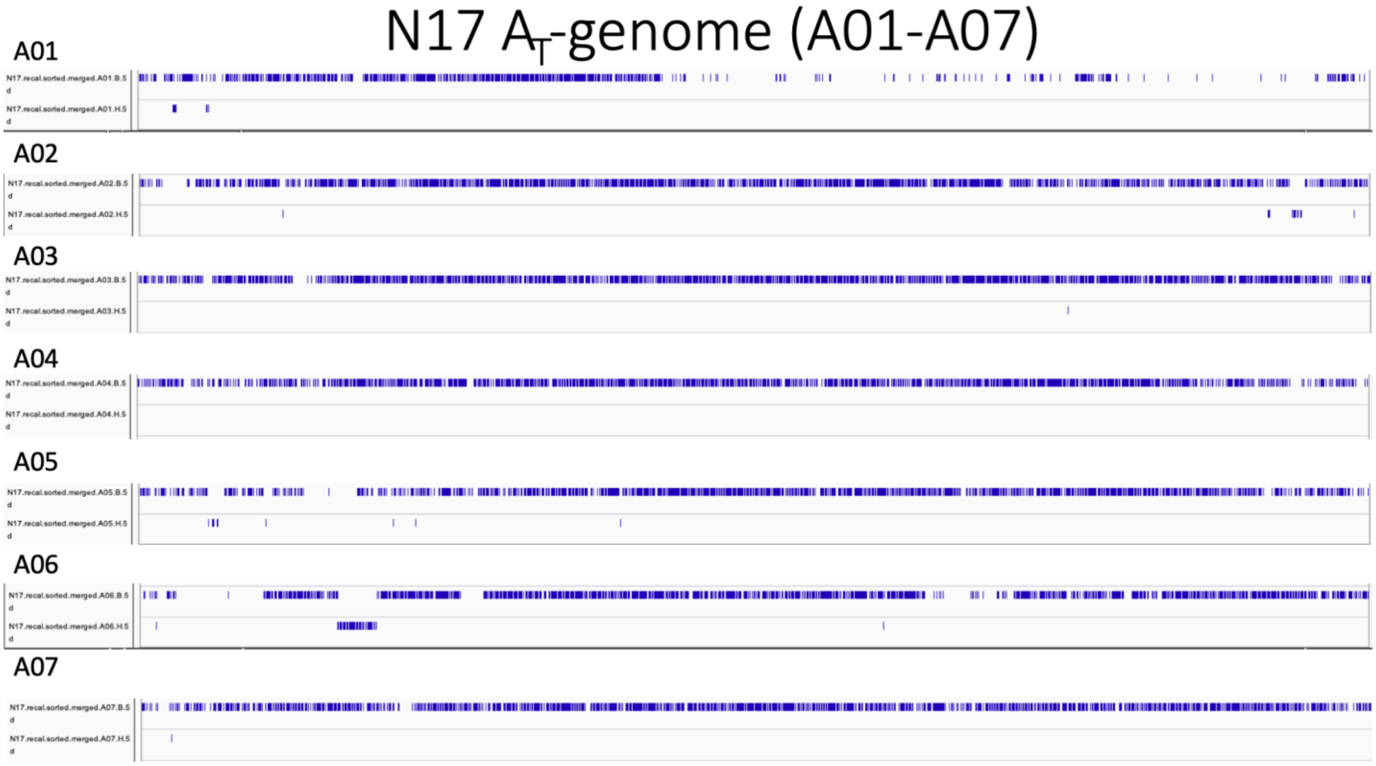

Supplementary Note 5, Fig. 5. Categorized region of chromosomes A01-A07 in *G. hirsutum* in the NIL N17 [23]. Each chromosome has two categorized tracks. The top track was categorized as ‘H’ for *G. hirsutum*, and the bottom track was categorized as ‘B’ for *G. barbadense*. Regions of introgression were detected on A02, A05, and A06. These additional introgressions existed in the *G. hirsutum* recurrent parent used for near isogenic line development. They are historical *G. barbadense* blocks common to nearly all accessions of *G. hirsutum* (see text). The ability to detect introgression was also dependent on the number of SNPs along the genome. Chromosome A01 had the largest number of ‘holes’ where the number of consecutive diagnostic SNPs limited detection.

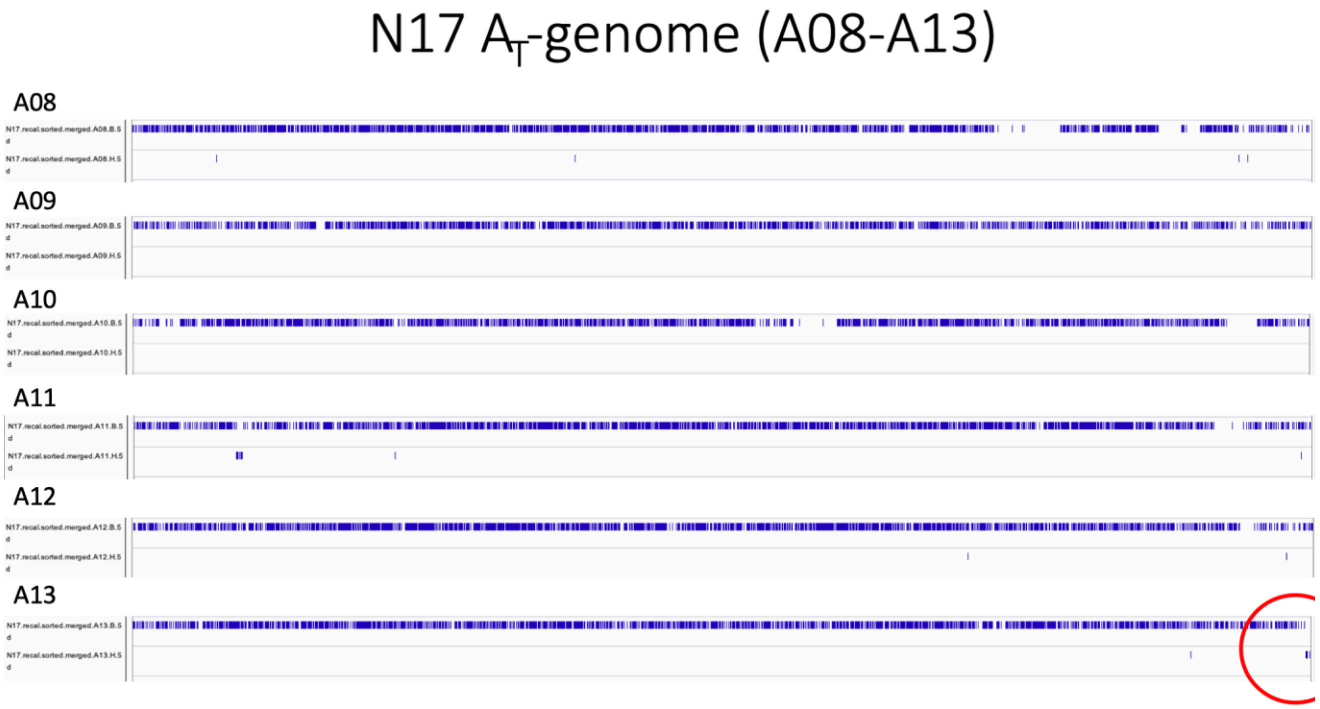

Supplementary Note 5, Fig. 6. Categorized region of chromosomes A08-A13 in *G. hirsutum* in the NIL N17 [23]. Each chromosome has two categorized tracks. The top track was categorized as 'H' for *G. hirsutum*, and the bottom track was categorized as 'B' for *G. barbadense*. A region of intentional introgression was detected on A13. A region of introgression was also detected on A11. These additional introgressions existed in the *G. hirsutum* recurrent parent used for near isogenic line development. They are historical *G. barbadense* blocks common to nearly all accessions of *G. hirsutum* (see text).

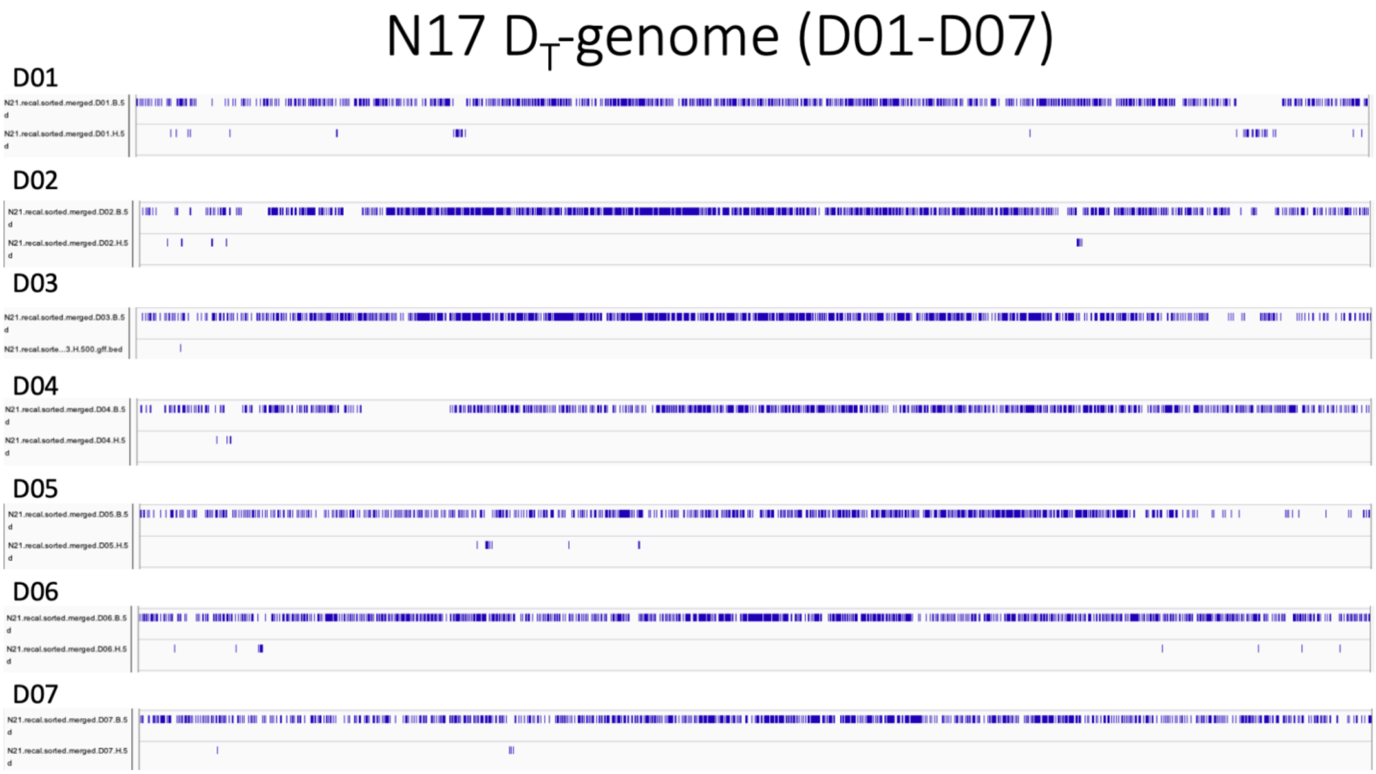

Supplementary Note 5, Fig. 7. Categorized region of chromosomes D01-D07 in *G. hirsutum* in the NIL N5 [23]. Each chromosome has two categorized tracks. The top track was categorized as ‘H’ for *G. hirsutum*, and the bottom track was categorized as ‘B’ for *G. barbadense*. No intentionally introgressed regions were detected. A region of introgression was also detected on D01, D02, D05, D06, and D07. These additional introgressions existed in the *G. hirsutum* recurrent parent used for near isogenic line development. They are historical *G. barbadense* blocks common to nearly all accessions of *G. hirsutum* (see text).

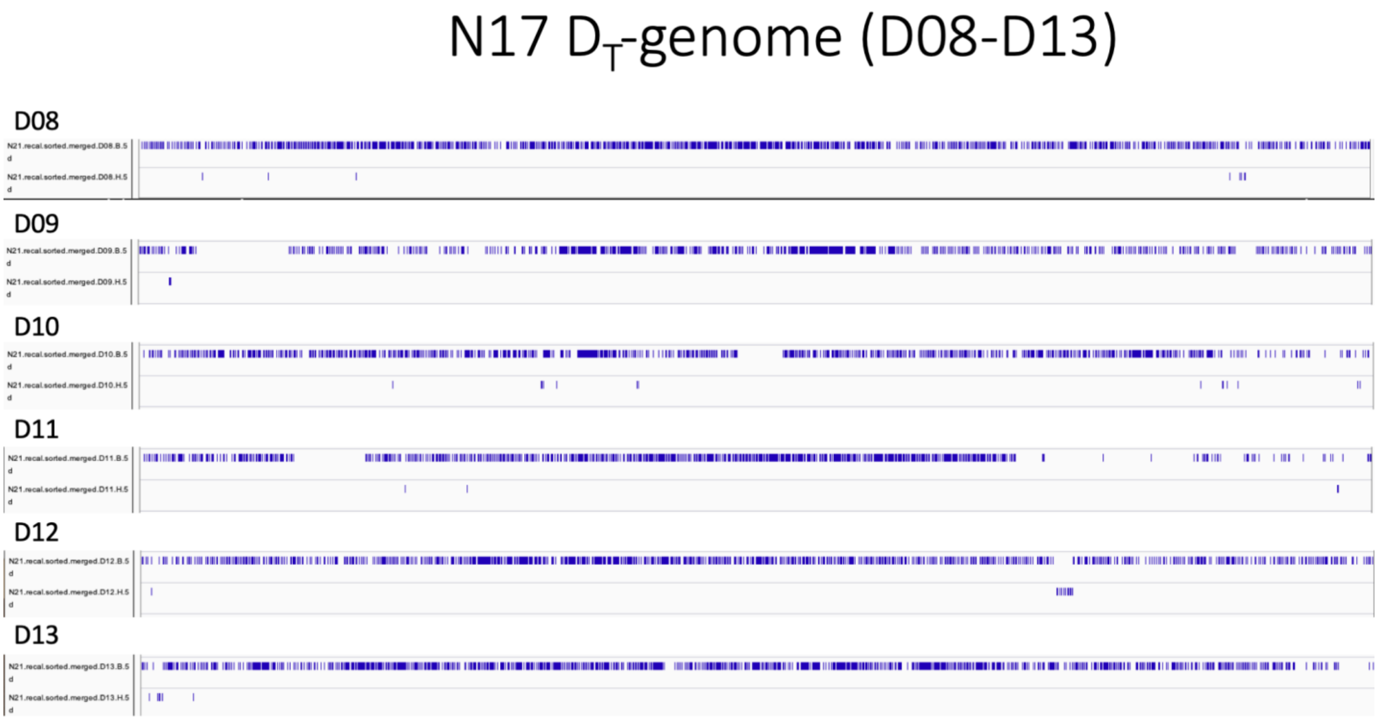

Supplementary Note 5, Fig. 8. Categorized region of chromosomes D08-D13 in *G. hirsutum* in the NIL N17 [23]. Each chromosome has two categorized tracks. The top track was categorized as ‘H’ for *G. hirsutum*, and the bottom track was categorized as ‘B’ for *G. barbadense*. No intentionally introgressed regions were detected, including the small segment at the end of D08. A region of introgression was also detected on D12. These additional introgressions existed in the *G. hirsutum* recurrent parent used for near-isogenic line development. They are historical *G. barbadense* blocks common to nearly all accessions of *G. hirsutum* (see text).

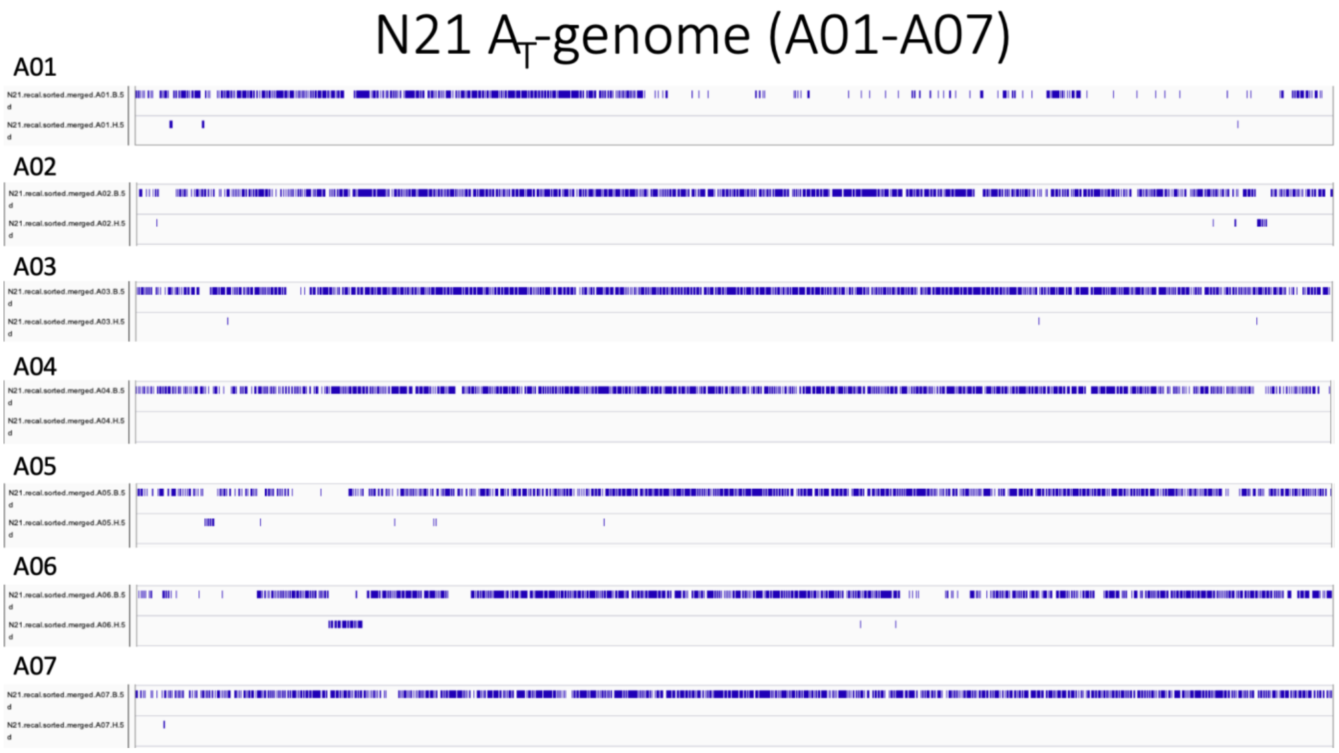

Supplementary Note 5, Fig. 9. Categorized region of chromosomes A01-A07 in *G. hirsutum* in the NIL N21 [23]. Each chromosome has two categorized tracks. The top track was categorized as 'H' for *G. hirsutum*, and the bottom track was categorized as 'B' for *G. barbadense*. Regions of introgression were detected on A02, A05, and A06. These additional introgressions existed in the *G. hirsutum* recurrent parent used for near isogenic line development. They are historical *G. barbadense* blocks common to nearly all accessions of *G. hirsutum* (see text). The ability to detect introgression was also dependent on the number of SNPs along the genome. Chromosome A01 had the largest number of 'holes' where the number consecutive diagnostic SNPs limited detection.

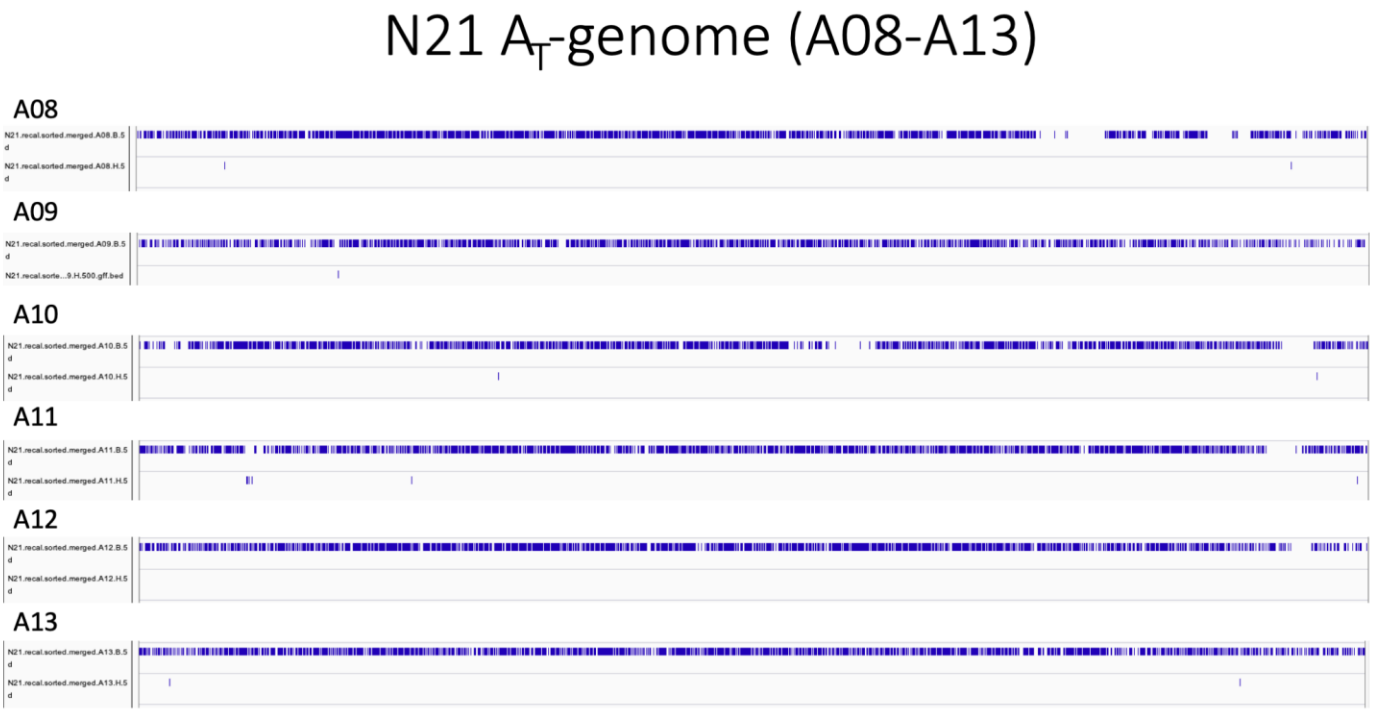

Supplementary Note 5, Fig. 10. Categorized region of chromosomes A08-A13 in *G. hirsutum* in the NIL N21 [23]. Each chromosome has two categorized tracks. The top track was categorized as 'H' for *G. hirsutum*, and the bottom track was categorized as 'B' for *G. barbadense*. A region of intentional introgression was detected on A13. A region of introgression was also detected on A11. These additional introgressions existed in the *G. hirsutum* recurrent parent used for near-isogenic line development. They are historical *G. barbadense* blocks common to nearly all accessions of *G. hirsutum* (see text).

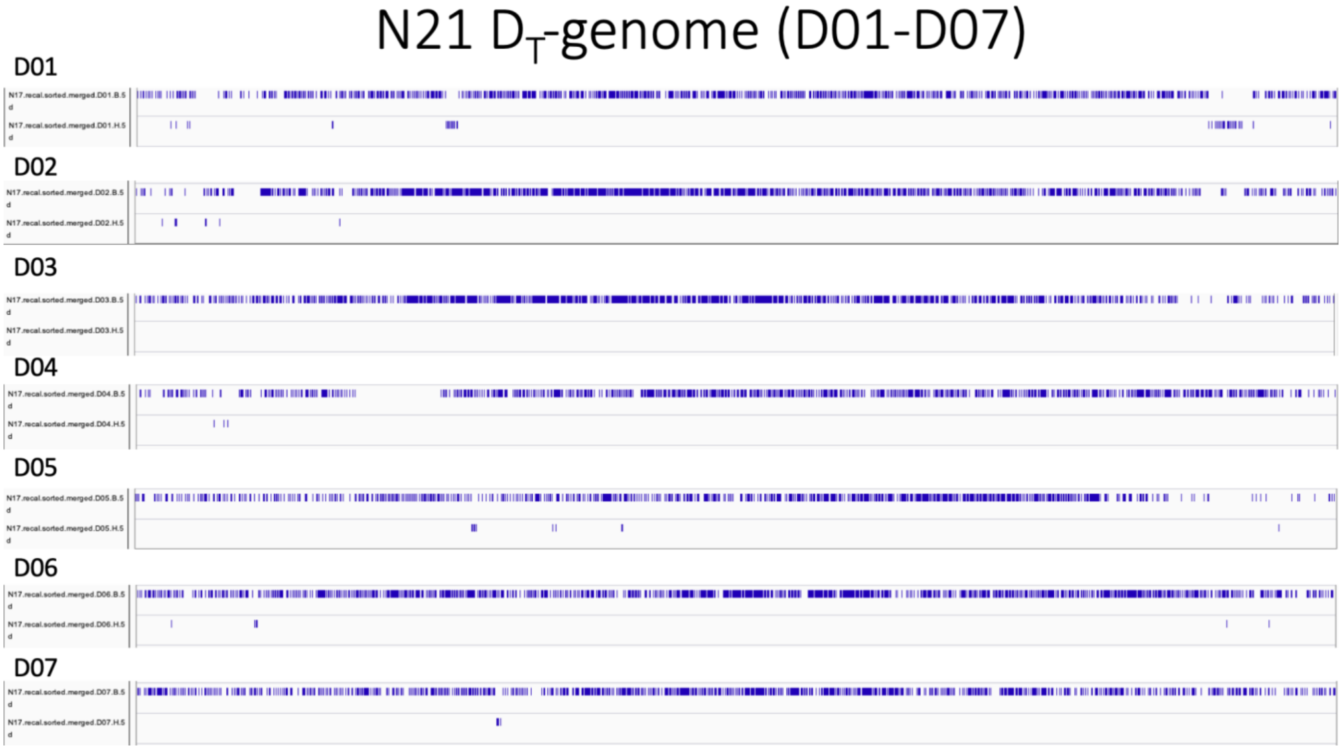

Supplementary Note 5, Fig. 11. Categorized region of chromosomes D01-D07 in *G. hirsutum* in the NIL N21 [23]. Each chromosome has two categorized tracks. The top track was categorized as ‘H’ for *G. hirsutum*, and the bottom track was categorized as ‘B’ for *G. barbadense*. No intentionally introgressed regions were detected. A region of introgression was also detected on D01, D02, D05, D06, and D07. These additional introgressions existed in the *G. hirsutum* recurrent parent used for near-isogenic line development. They are historical *G. barbadense* blocks common to nearly all accessions of *G. hirsutum* (see text).

N21 D_T-genome (D08-D13)

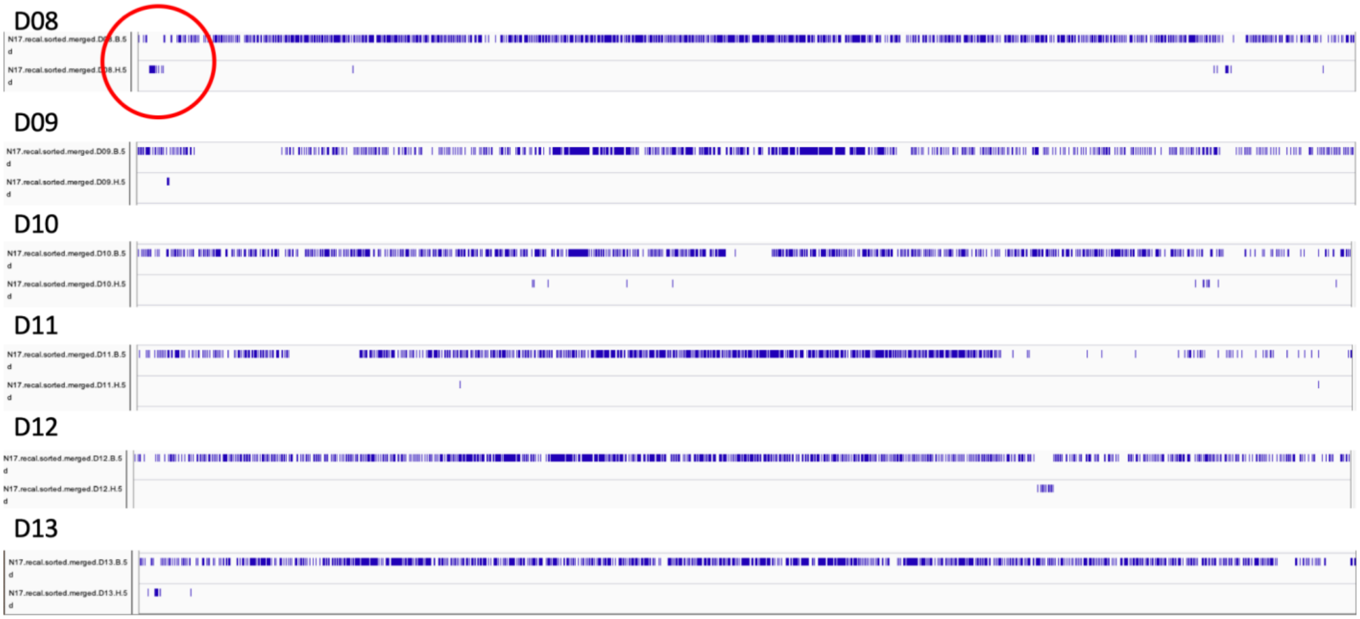

Supplementary Note 5, Fig. 12. Categorized region of chromosomes D08-D13 in *G. hirsutum* in the NIL N21 [23]. Each chromosome has two categorized tracks. The top track was categorized as ‘H’ for *G. hirsutum*, and the bottom track was categorized as ‘B’ for *G. barbadense*. An intentionally introgressed region was detected on D08 (note that the positions in the table of Wang et al., 2019 are the reverse complement of the reference sequence used in this study). A region of introgression was also detected on D12. These additional introgressions existed in the *G. hirsutum* recurrent parent used for near-isogenic line development. They are historical *G. barbadense* blocks common to nearly all accessions of *G. hirsutum* (see text).

Literature Cited

1. P. Tyagi, M.A. Gore, D.T. Bowman, B.T. Campbell, J.A. Udall, V. Kuraparthy, *Theor. Appl. Genet.* **2014**, **127**, 283.
2. L.L. Hinze, D.D. Fang, M.A. Gore, B.E. Scheffler, J.Z. Yu, J. Frelichowski, R.G. Percy, *Theor. Appl. Genet.* **2015**, **128**, 313.
3. M. Wang, L. Tu, M. Lin, Z. Lin, P. Wang, Q. Yang, Z. Ye, C. Shen, J. Li, L. Zhang, X. Zhou, X. Nie, Z. Li, K. Guo, Y. Ma, C. Huang, S. Jin, L. Zhu, X. Yang, L. Min, D. Yuan, Q. Zhang, K. Lindsey, X. Zhang, *Nat. Genet.* **2017**, **49**, 579.
4. L. Fang, Q. Wang, Y. Hu, Y. Jia, J. Chen, B. Liu, Z. Zhang, X. Guan, S. Chen, B. Zhou, G. Mei, J. Sun, Z. Pan, S. He, S. Xiao, W. Shi, W. Gong, J. Liu, J. Ma, C. Cai, X. Zhu, W. Guo, X. Du, T. Zhang, *Nat. Genet.* **2017**, **49**, 1089.
5. L. Fang, H. Gong, Y. Hu, C. Liu, B. Zhou, T. Huang, Y. Wang, S. Chen, D.D. Fang, X. Du, H. Chen, J. Chen, S. Wang, Q. Wang, Q. Wan, B. Liu, M. Pan, L. Chang, H. Wu, G. Mei, D. Xiang, X. Li, C. Cai, X. Zhu, Z.J. Chen, B. Han, X. Chen, W. Guo, T. Zhang, X. Huang, *Genome Biol.* **2017**, **18**, 33.
6. M. Wang, L. Tu, M. Lin, Z. Lin, P. Wang, Q. Yang, Z. Ye, C. Shen, J. Li, L. Zhang, X. Zhou, X. Nie, Z. Li, K. Guo, Y. Ma, C. Huang, S. Jin, L. Zhu, X. Yang, L. Min, D. Yuan, Q. Zhang, K. Lindsey, X. Zhang, *Nat. Genet.* **2017**, **49**, 579.
7. L. Fang, Q. Wang, Y. Hu, Y. Jia, J. Chen, B. Liu, Z. Zhang, X. Guan, S. Chen, B. Zhou, G. Mei, J. Sun, Z. Pan, S. He, S. Xiao, W. Shi, W. Gong, J. Liu, J. Ma, C. Cai, X. Zhu, W. Guo, X. Du, T. Zhang, *Nat. Genet.* **2017**, **49**, 1089.
8. L. Fang, H. Gong, Y. Hu, C. Liu, B. Zhou, T. Huang, Y. Wang, S. Chen, D.D. Fang, X. Du, H. Chen, J. Chen, S. Wang, Q. Wang, Q. Wan, B. Liu, M. Pan, L. Chang, H. Wu, G. Mei, D. Xiang, X. Li, C. Cai, X. Zhu, Z.J. Chen, B. Han, X. Chen, W. Guo, T. Zhang, X. Huang, *Genome Biol.* **2017**, **18**, 33.
9. B.S. Weir, C.C. Cockerham, *Evolution* **1984**.
10. P. Danecek, A. Auton, G. Abecasis, C.A. Albers, E. Banks, M.A. DePristo, R.E. Handsaker, G. Lunter, G.T. Marth, S.T. Sherry, G. McVean, R. Durbin, 1000 Genomes Project Analysis Group, *Bioinformatics* **2011**, **27**, 2156.
11. Y. Hu, J. Chen, L. Fang, Z. Zhang, W. Ma, Y. Niu, L. Ju, J. Deng, T. Zhao, J. Lian, K. Baruch, D. Fang, X. Liu, Y.-L. Ruan, M.-U. Rahman, J. Han, K. Wang, Q. Wang, H. Wu, G. Mei, Y. Zang, Z. Han, C. Xu, W. Shen, D. Yang, Z. Si, F. Dai, L. Zou, F. Huang, Y. Bai, Y. Zhang, A. Brodt, H. Ben-Hamo, X. Zhu, B. Zhou, X. Guan, S. Zhu, X. Chen, T. Zhang, *Nat. Genet.* **2019**, **51**, 739.
12. Y. Chen, Y. Chen, C. Shi, Z. Huang, Y. Zhang, S. Li, Y. Li, J. Ye, C. Yu, Z. Li, X. Zhang, J. Wang, H. Yang, L. Fang, Q. Chen, *Gigascience* **2018**, **7**, 1.
13. C.A. Saski, B.E. Scheffler, A.M. Hulse-Kemp, B. Liu, Q. Song, A. Ando, D.M. Stelly, J.A. Scheffler, J. Grimwood, D.C. Jones, D.G. Peterson, J. Schmutz, Z. Jeffery Chen, *Scientific Reports*. 2017.
14. A. Dobin, C.A. Davis, F. Schlesinger, J. Drenkow, C. Zaleski, S. Jha, P. Batut, M. Chaisson, T.R. Gingeras, *Bioinformatics* **2013**, **29**, 15.
15. C. Trapnell, A. Roberts, L. Goff, G. Pertea, D. Kim, D.R. Kelley, H. Pimentel, S.L. Salzberg, J.L. Rinn, L. Pachter, *Nat. Protoc.* **2012**, **7**, 562.
16. A. Jain, G. Tuteja, *Bioinformatics* **2019**, **35**, 1966.
17. Y. Bao, G. Hu, C.E. Grover, J. Conover, D. Yuan, J.F. Wendel, *Nature Communications* **2019**, **10**, 1.

18. T. Metsalu, J. Vilo, *Nucleic Acids Res.* **2015**, **43**, W566.
19. G. Yu, L.-G. Wang, Y. Han, Q.-Y. He, *OMICS* **2012**, **16**, 284.
20. J.T. Page, Z.S. Liechty, R.H. Alexander, K. Clemons, A.M. Hulse-Kemp, H. Ashrafi, A. Van Deynze, D.M. Stelly, J.A. Udall, *PLoS Genet.* **2016**, **12**, e1006012.
21. J.T. Page, Z.S. Liechty, M.D. Huynh, J.A. Udall, *BMC Res. Notes* **2014**, **7**, 829.
22. A.R. Quinlan, *Curr. Protoc. Bioinformatics* **2014**, **47**, 11.
23. M. Wang, L. Tu, D. Yuan, D. Zhu, C. Shen, J. Li, F. Liu, L. Pei, P. Wang, G. Zhao, Z. Ye, H. Huang, F. Yan, Y. Ma, L. Zhang, M. Liu, J. You, Y. Yang, Z. Liu, F. Huang, B. Li, P. Qiu, Q. Zhang, L. Zhu, S. Jin, X. Yang, L. Min, G. Li, L.-L. Chen, H. Zheng, K. Lindsey, Z. Lin, J.A. Udall, X. Zhang, *Nat. Genet.* **2019**, **51**, 224.
24. R Core Team, R: A language and environment for statistical computing; R Foundation for Statistical Computing., Vienna, Austria, 2017.
25. H. Wickham, Others, *J. Stat. Softw.* **2007**, **21**, 1.
