# Supplementary material for: Parallel and Intertwining Threads of Domestication in Allopolyploid Cotton
Source: Adv Sci (Weinh). 2021 Mar 15;8(10):2003634. doi: 10.1002/advs.202003634 (PMC8132148; doi:10.1002/advs.202003634)

Supplemental Note 1, Figure 1

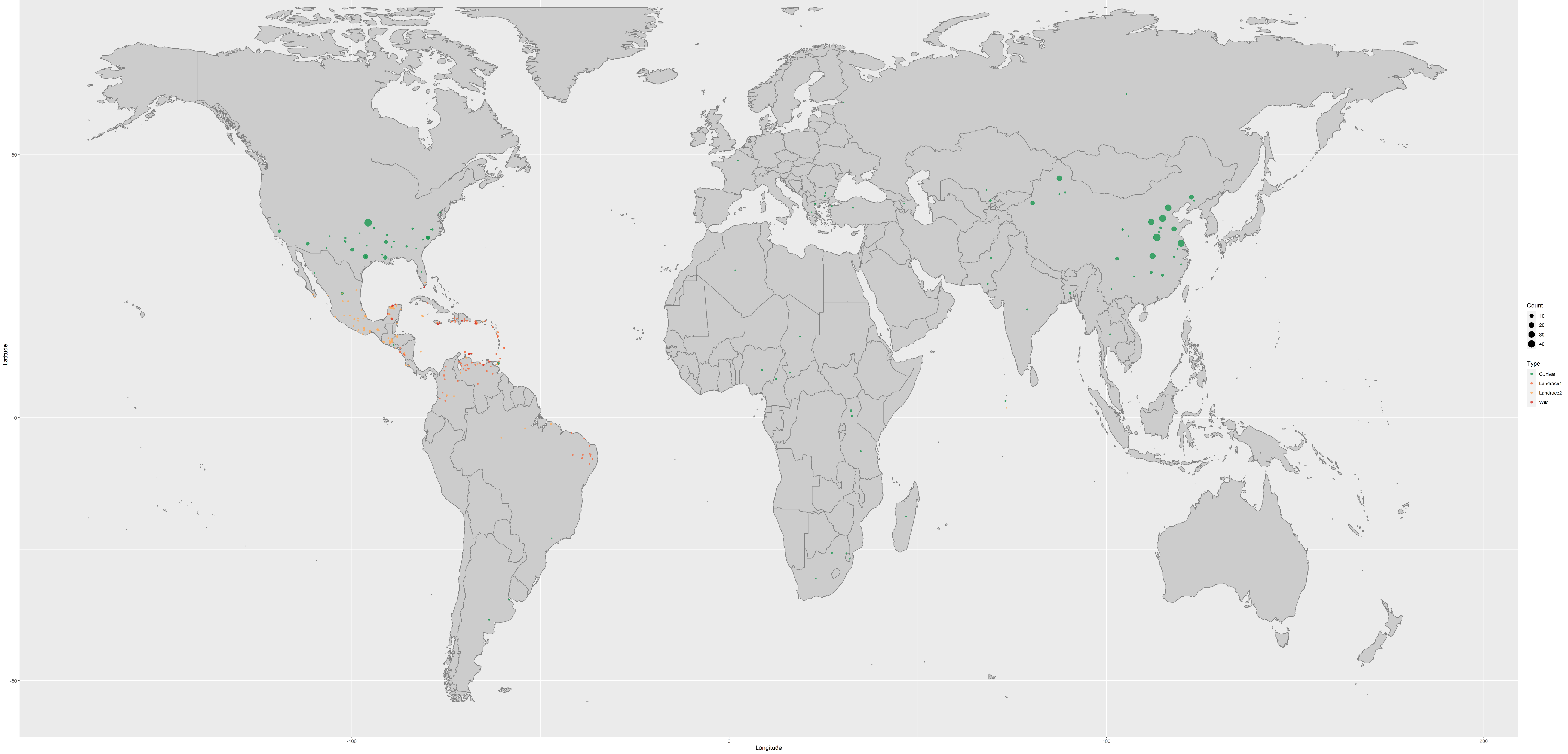

Supplemental Note 1, Figure 2

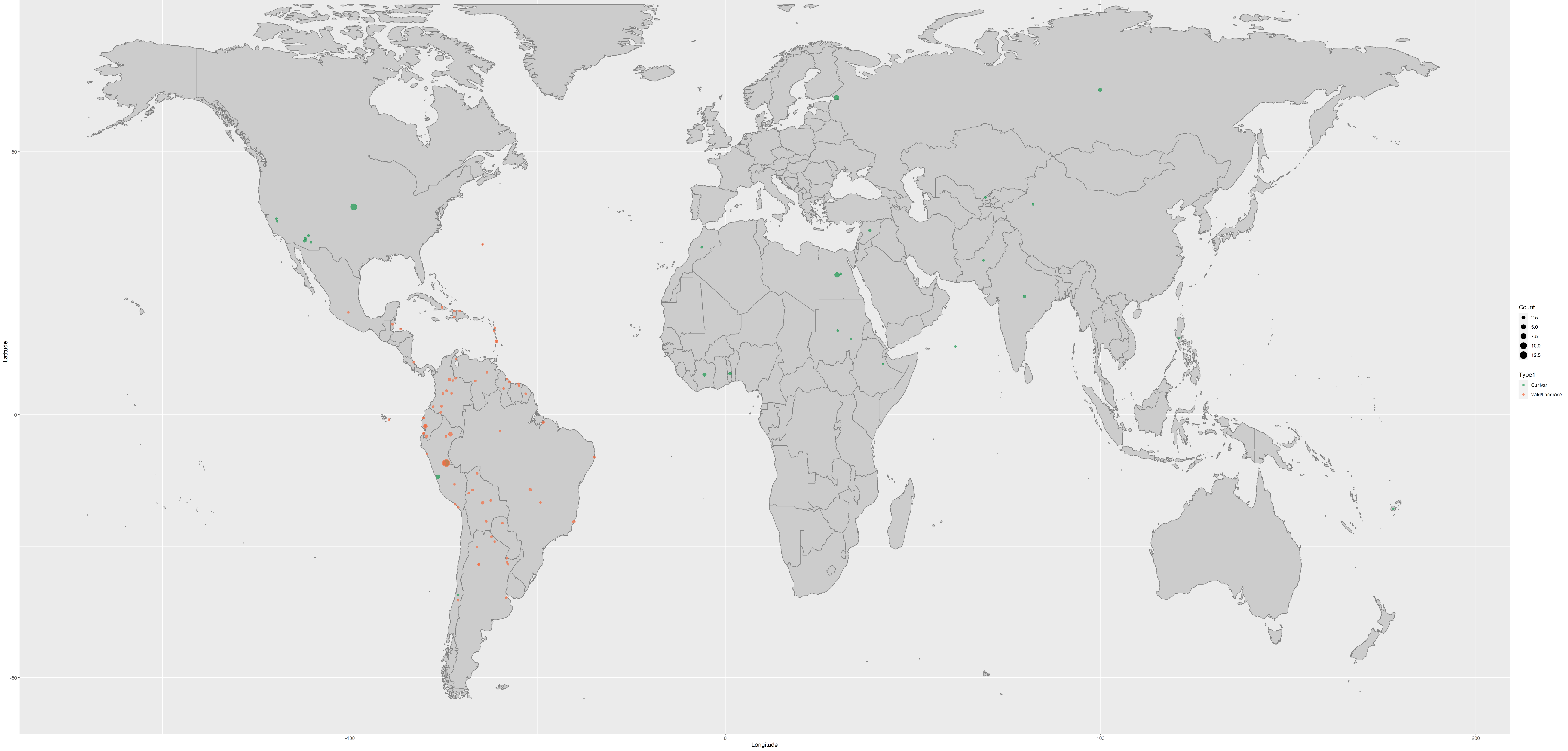

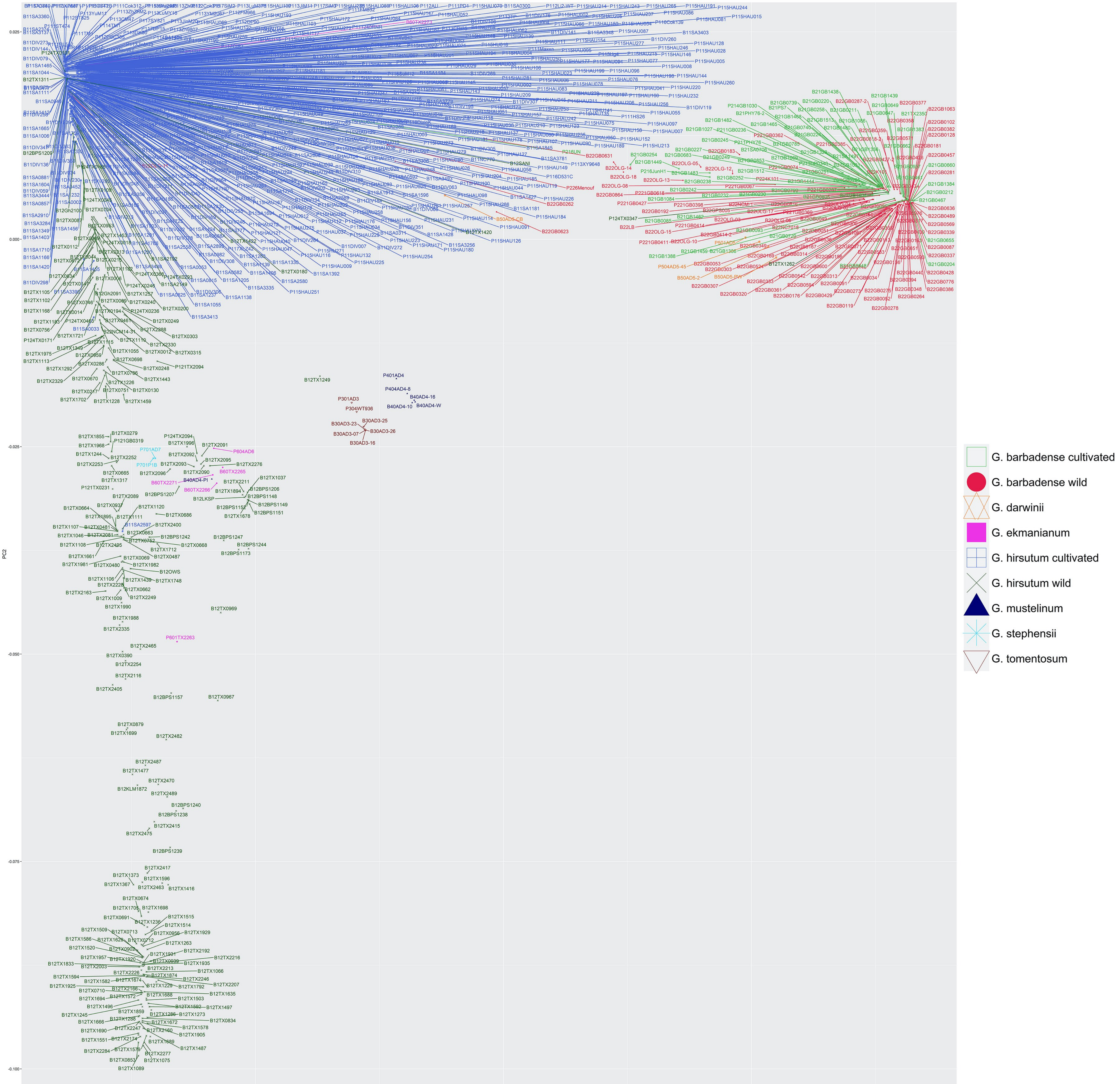

Supplemental Note 3, Figure 3

Supplemental Note 3, Figure 7

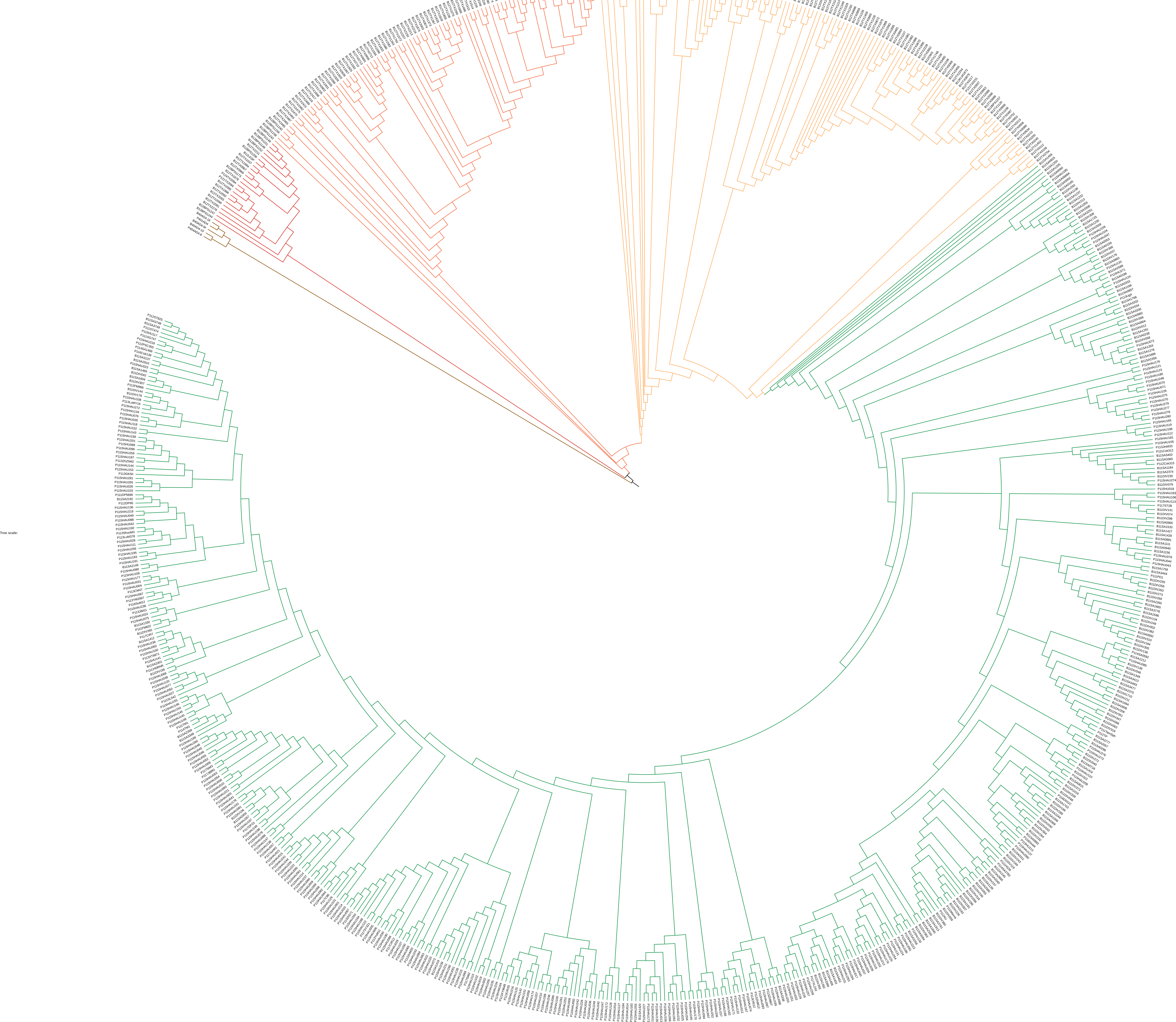

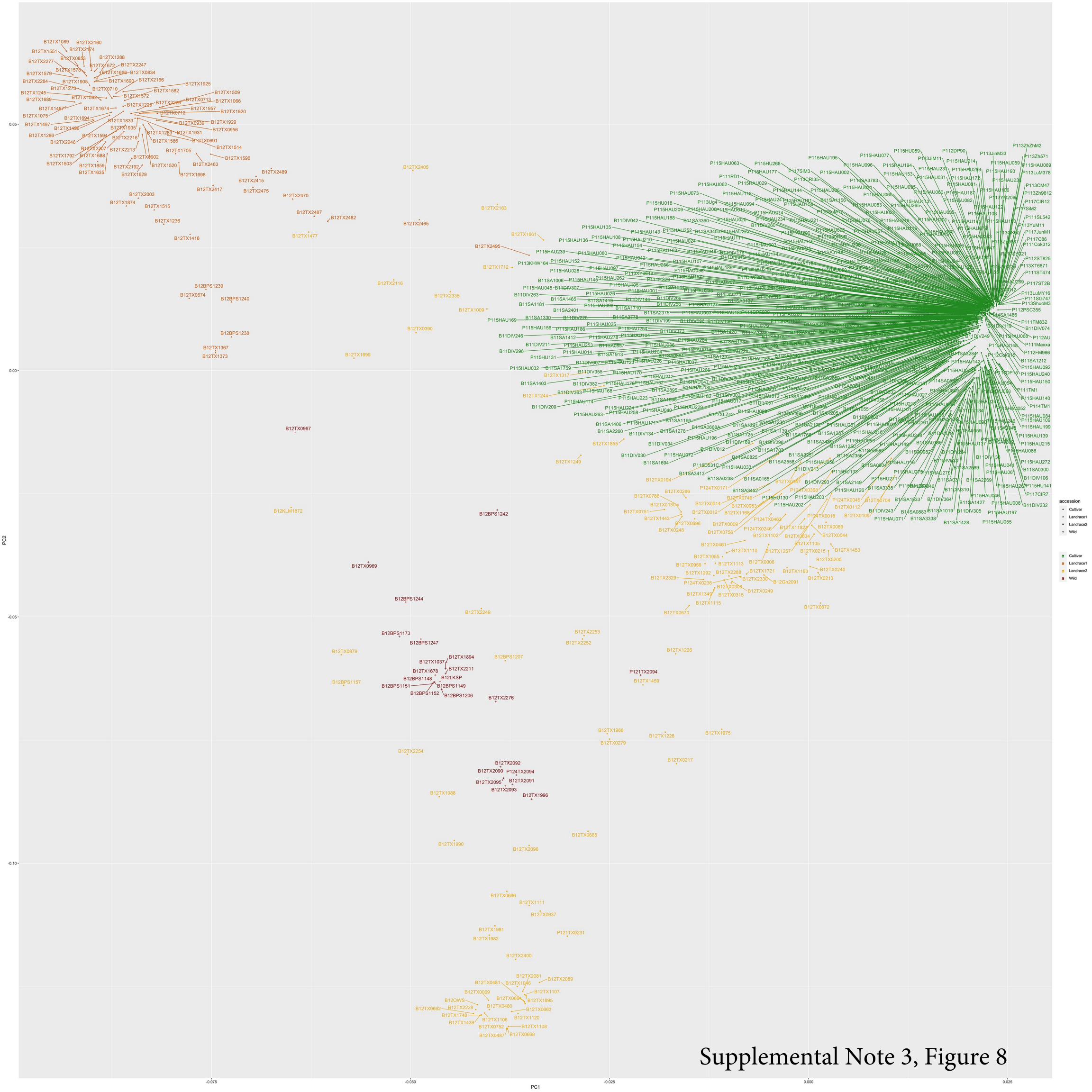

Supplemental Note 3, Figure 8

Tree scale:

Supplemental Note 3, Figure 9

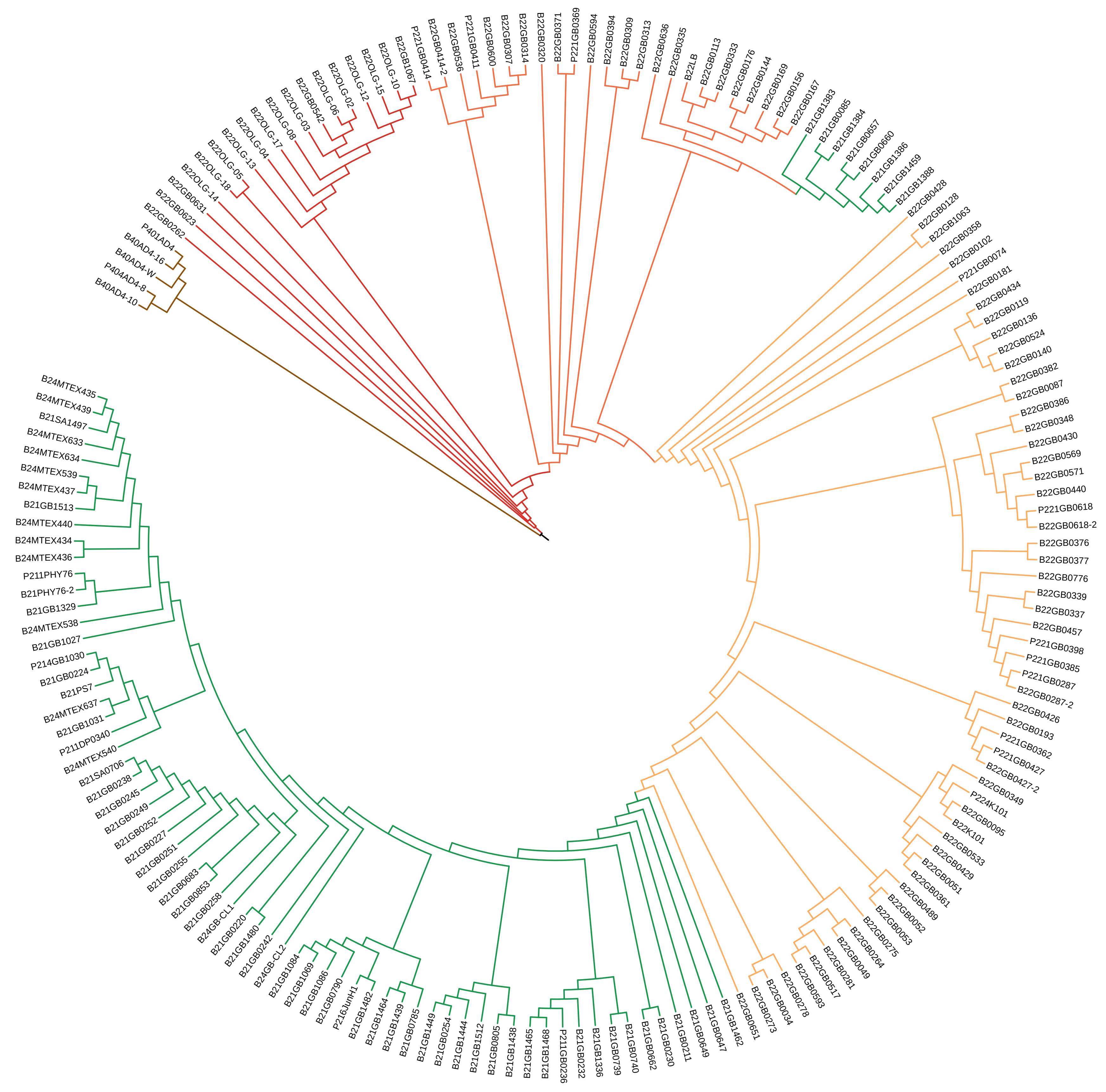

Supplemental Note 3, Figure 10

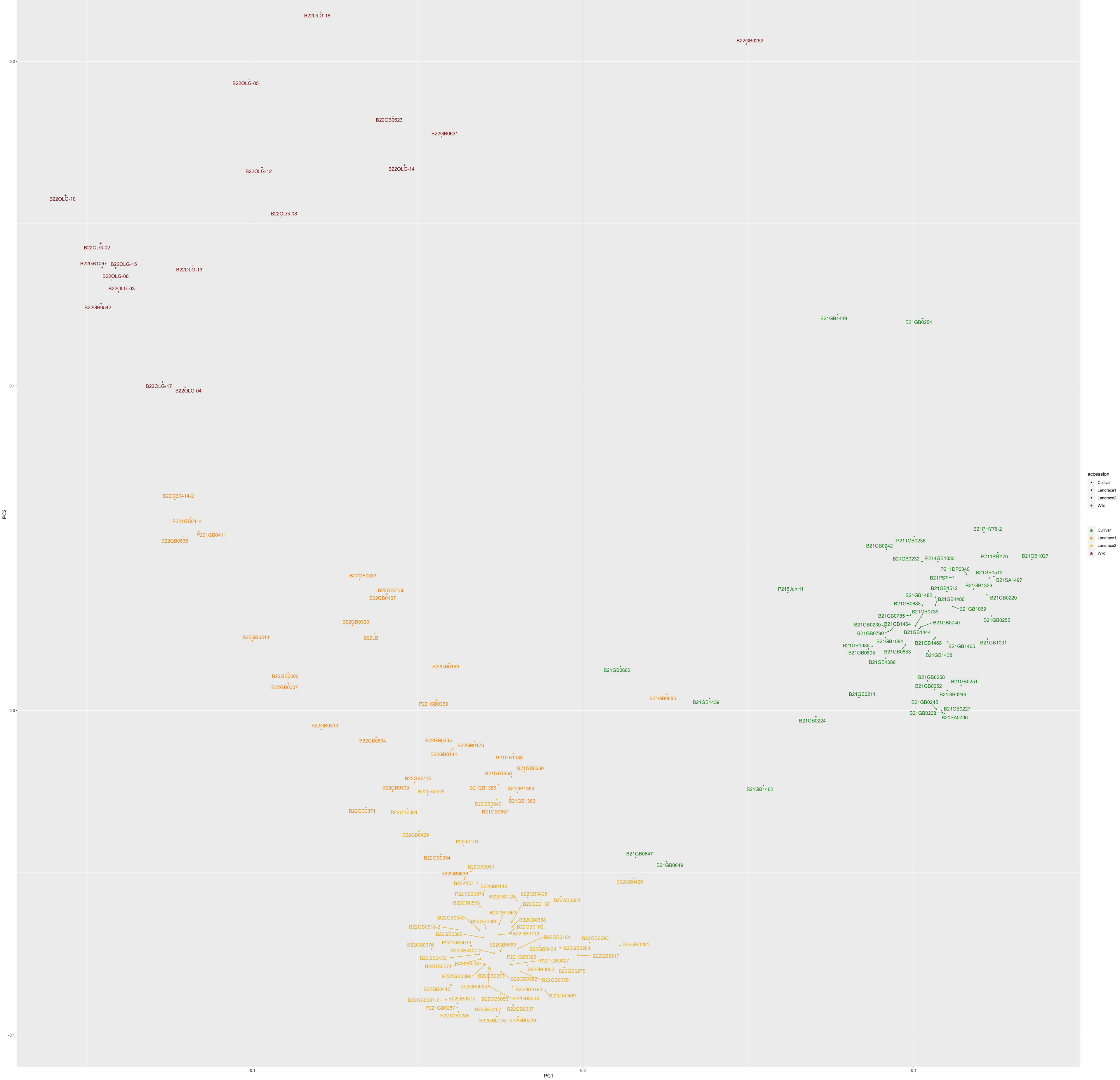

Supplement: Supplementary file 2 — Supporting Figures [file ADVS-8-2003634-s002.pdf]
